# Causality between sleep traits and the risk of frailty: a Mendelian randomization study

**DOI:** 10.3389/fpubh.2024.1381482

**Published:** 2024-05-09

**Authors:** Zhen Deng, Yifan Hu, Lincheng Duan, Ziding Buyang, Qian Huang, Xuedan Fu, Hong Luo, Tianshu Hou

**Affiliations:** ^1^Chengdu Integrated Traditional Chinese Medicine and Western Medicine Hospital, Chengdu, China; ^2^Chengdu University of Traditional Chinese Medicine, Chengdu, China

**Keywords:** sleep traits, frailty, Mendelian randomization, causal relationship, frailty index

## Abstract

**Background:**

Research based on observation has demonstrated a relationship between sleep traits and frailty; however, it remains uncertain if this correlation indicates causation. The purpose of this study was to look at the causal relationship that exists between frailty and sleep traits.

**Method:**

Using summaries from a genome-wide association study of self-reported sleep features and frailty index, we performed a bidirectional Mendelian randomization (MR) analysis. Examining the causal relationships between seven sleep-related traits and frailty was the goal. The major method used to calculate effect estimates was the inverse-variance weighted method, supplemented by the weighted median and MR-Egger approaches. The study investigated pleiotropy and heterogeneity using several methodologies, such as the MR-Egger intercept, the MR-PRESSO approach, and the Cochran’s Q test. We took multivariate Mendelian randomization and genetic correlations between related traits to enhance the confidence of the results. Furthermore, we used MRlap to correct for any estimation bias due to sample overlap.

**Results:**

Insomnia, napping during the day, and sleep apnea syndrome exhibited a positive connection with the frailty index in forward MR analysis. Conversely, there is a negative link between getting up in the morning, snoring and sleep duration with the frailty index. During the reverse MR analysis, the frailty index exhibited a positive correlation with insomnia, napping during the day, and sleep apnea syndrome, while demonstrating a negative correlation with sleep duration. There was no direct correlation between snoring, chronotype, and frailty. In MVMR analyses, the causal effect of sleep characteristics on frailty indices remained consistent after adjusting for potential confounders including BMI, smoking, and triglycerides.

**Conclusion:**

The findings of our investigation yield novel evidence that substantiates the notion of a bidirectional causal connection between sleep traits and frailty. Through the optimization of sleep, it is potentially feasible to hinder, postpone, or even reverse the state of frailty, and we proposed relevant interventions.

## Introduction

Common sleep issues, encompassing insomnia, insufficient sleep length, obstructive sleep apnea, and daytime napping, are increasingly prevalent among individuals due to changing lifestyles. Sleep is crucial for normal body growth, development, and memory consolidation. Over the past few decades, the global prevalence of sleep disorders has risen, with approximately half of older adults reporting sleep issues (1).

Frailty is a condition marked by heightened susceptibility to stress due to the gradual breakdown of multiple physiological systems (2, 3), which reduces quality of life and demands a lot of money for healthcare and caregiving (4–8). In older people living in the community, a recent systematic review and meta-analysis indicated that prefrailty ranged from 31.3 to 45.8% and frailty from 10.4 to 37.0% (9). The study shows that 10% of older adult individuals are frail, while 40% are pre-frail (10). Conditions such as fatigue, depression, and sleep–wake disorders can lead to frailty (11). Frailty can be prevented and recovered with risk-factor interventions (12, 13).

Increasing research links subjective sleep measurements to widespread frailty (11, 14–19). Objective sleep markers also show this relationship (5). A Chinese cohort study found a significant link between increased sleep duration and frailty (20). Longer and shorter sleep durations increase frailty risk, according to systematic reviews and meta-analyses (21). Other than that, older adult individuals who suffer from sleep disorders are more vulnerable to frailty (21–23). In addition, sleep disorders interact with frailty, which ultimately leads to a decrease in physical performance (24). However, confounding and selection biases present in conventional observational studies may affect the associations between sleep-related factors and frailty. The majority of the included studies are cross-sectional, which makes it difficult to determine a causal association between the amount of sleep and frailty. Second, these studies rely on subjects’ recall and self-report, which can lead to recall bias. Furthermore, studies looking into the connection between frailty and sleep disturbances have yielded inconsistent results (25–27). These studies ranged from 4.7 to 8 years of follow-up, respectively, and the number of people studied varied considerably. All of these reasons may have led to differences in the difficulty of their tracking and control. Therefore, it is imperative that the relationship between sleep problems and frailty be thoroughly explored.

Because large-scale randomized trials covering a variety of sleep problems are difficult to undertake, other study designs, like Mendelian randomization (MR), are required. To overcome the shortcomings of observational research, MR makes use of genetic data within an instrumental variable (IVs) framework (28). By eliminating confounding variables and reverse causality bias, MR can be used instead of standard observational multivariate regression, avoiding common problems with observational research (29). Accordingly, the aim of this study was to determine the causal link between sleep characteristics and frailty using a two-sample MR analysis.

## Methods

### Study design

**Figure 1:**
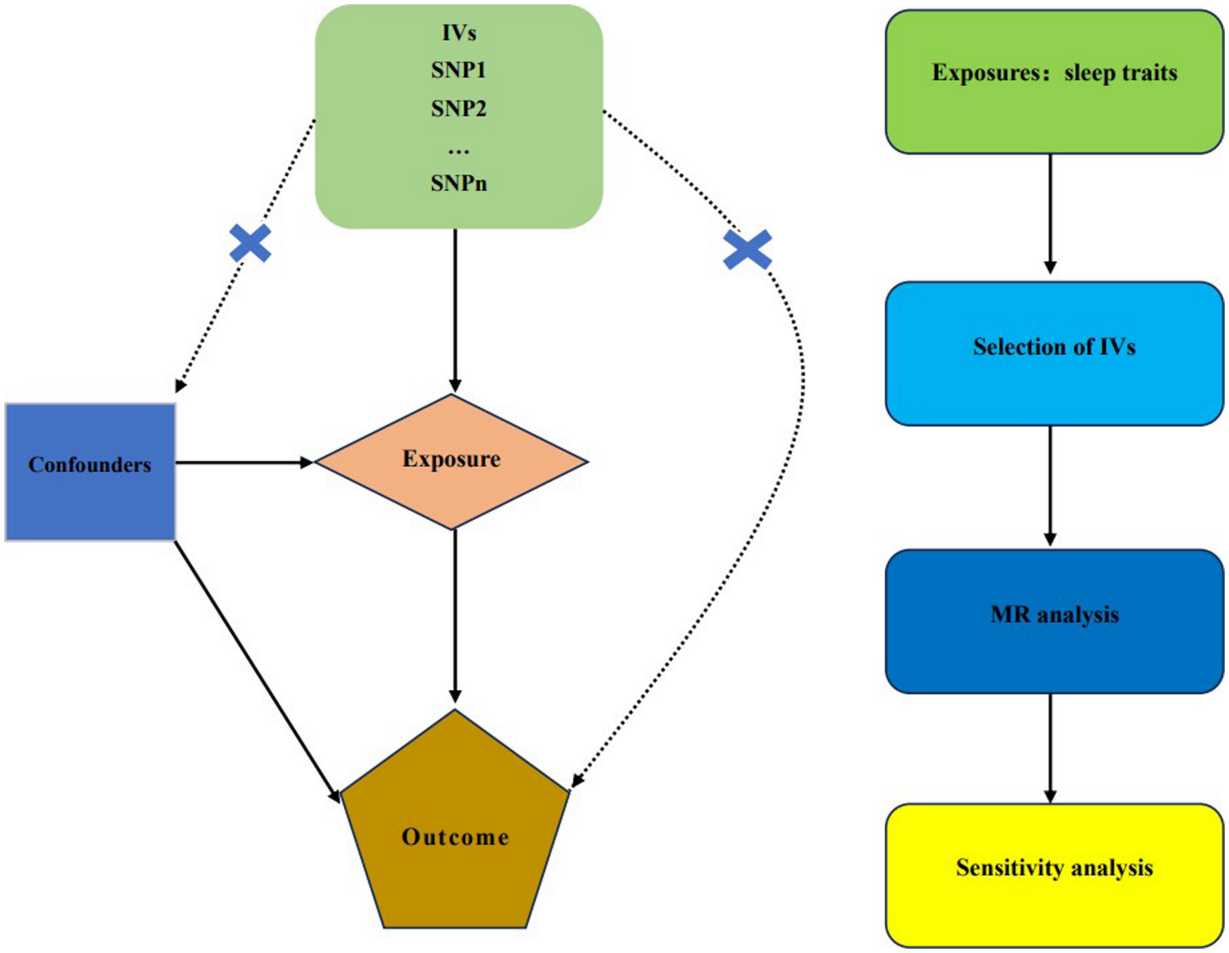
Our MR study’s study design. MR, Mendelian randomization; IVs, instrumental variables; SNP, single-nucleotide polymorphism.

Using the largest publicly available genome-wide association study (GWAS) datasets, we used a two-sample MR analysis to investigate the association between sleep traits and the risk of frailty index. Three presumptions are required to be met for MR research to guarantee successful causal reasoning: (1) genetic IVs that are highly connected to exposure; (2) genetic IVs are not connected to possible confounding factors; and (3) only certain genetic IVs affected by exposures, excluding other means (30). Figure 1 shows a design for a bidirectional MR.

### Data sources

#### Data sources for sleep phenotypes

Trouble falling or remaining asleep for the required amount of time is known as insomnia, a common sleep condition. A GWAS involving 462,341 samples revealed a genetic relationship with insomnia. The assessment of insomnia involved asking the following question: “Do you find it difficult to fall asleep at night or do you frequently wake up during the night?”

The UK Biobank data (*N* = 460,099) served as the basis for a GWAS that produced summary statistics on sleep duration. The question “In a 24-h period, how many hours do you sleep?” was used to gage sleep duration.

A GWAS involving 461,658 people yielded the genetic relationship for getting up in the morning.

A person’s circadian preference—the inclination to sleep earlier or later—defines their chronotype. Results from a GWAS of 413,343 people with European ancestry yielded genetic association estimates for chronotype.

The genetic correlation with napping during the day was obtained from a GWAS, which included a total of 462,400 samples.

The assessment of snoring involved inquiring, “Do your partner, a close relative, or a friend express dissatisfaction with your snoring?” Genetic association estimates for snoring were obtained from a GWAS conducted on a population of 430,438 individuals of European descent.

The definition of sleep apnea syndrome is “a disorder characterized by repeated breathing cessations during sleep that interfere with sleep maintenance and cause partial arousals.” 476,853 adults of European descent with published GWAS associations provided the genetic association data for sleep apnea syndrome.

#### Data sources for frailty

In this MR study, frailty was assessed using the frailty index (FI), which is determined by the accumulation of 44–49 self-reported health deficits over an individual’s life course. FI provides a common definition of frailty. A recent meta-analysis of GWAS on individuals of European heritage from the UK Biobank and Swedish TwinGene, comprising a total sample size of 175,226 individuals, provided summary statistics for FI. All the data are shown in Table 1.

**Table 1:** The study’s GWAS data source details.

Phenotype	Data source	Consortium	PMID	Sample size	Ancestry
Insomnia	IEU OpenGWAS	MRC-IEU	–	462,341	European
Getting up in morning	IEU OpenGWAS	MRC-IEU	–	461,658	European
Chronotype	IEU OpenGWAS	MRC-IEU	–	413,343	European
Nap during day	IEU OpenGWAS	MRC-IEU	–	462,400	European
Sleep duration	IEU OpenGWAS	MRC-IEU	-	460,099	European
Sleep apnea syndrome	IEU OpenGWAS	NA	34594039	476,853	European
Snoring	IEU OpenGWAS	MRC-IEU	–	430,438	European
Frailty index	IEU OpenGWAS	NA	34431594	175,226	European

#### Instrumental selection

Firstly, we looked through the linked GWAS pooled datasets and found SNPs that had a strong correlation (*p* < 5.00E−08) with sleep traits. To eliminate the possibility of linkage disequilibrium among these SNPs (*r*^2^ < 0.001, clump window >10,000 kb), one thousand genomes from a European population served as our reference collection. Additionally, palindromic SNPs were manually removed. The remaining SNPs were then used as instrument variables after these operations. Additionally, we evaluated each SNP’s statistical efficacy using the F-statistic (F = β^2^/se^2^) (31) and removed any that had poor statistical efficacy in order to reduce mild instrumental bias (*F* > 10). Also, we lowered the GWAS *p*-value cutoff to 5.00E−06 for sleep apnea syndrome to ensure an adequate number of SNPs for the MR analysis.

#### Univariable Mendelian randomization analysis

With the use of genome-wide significant IVs, the two-sample MR approach calculated the causal influence of exposure and risk variables on particular outcomes. To achieve this, we used a variety of complementary techniques, such as weighted medians, MR-Egger, and IVW. In our major analyses, we evaluated the causal relationship between sleep traits and frailty index using the IVW approach. When dealing with exposures with multiple IVs, we thought that the most effective way to estimate causal effects was to use the IVW method with multiplicative random effects. Therefore, our main method of analysis for MR was the IVW method (32). In the case of significant heterogeneity between IVs (Q_pval <0.05), the MR effect size was estimated using random-effect IVW. Otherwise, fixed-effect IVW was adopted (33).

#### MRlap analysis

We cannot rule out sample overlap because our summary statistics are only based on European populations. In order to reduce any bias that might have resulted from our inability to directly determine the sample overlap rate (34), we corrected the IVW results using the MRlap function. It is reasonable to have trust in the IVW-MR estimations If the disparity between the observed and adjusted effect is not significant (*p* > 0.05). Conversely, if we find a significant difference (*p* < 0.05), we should prioritize the adjusted effect, which remains unaffected by sample overlap.

#### Sensitivity analysis

In order to evaluate the resilience of the findings, multiple sensitivity analyses were carried out. First, the Cochran’s Q test was used to quantify the IVW’s heterogeneity (35), and funnel plots were used to display the results. Second, scatterplots were employed to present the outcomes of the MR-Egger intercept test, which was utilized to examine pleiotropy (36). The MR-PRESSO test is utilized not only for identifying outliers and estimating causal effects once the corresponding outliers have been removed but also for assessing the presence of horizontal pleiotropy (37). We also ran a leave-one-out analysis to see if any particular SNP affected the outcomes considerably. The entire analysis was performed using the packages “TwoSampleMR” (version 0.5.6), “MRPRESSO” (version 1.0), and “MVMR” in tandem with RStudio (version 4.2.2).

#### Multivariable Mendelian randomization

To test the second core hypothesis of MR and to mitigate the effects of variables that may distort UVMR results, MVMR was used to assess the relationship between exposure, confounders, and outcomes. We reviewed the literature to identify common risk factors associated with frailty, including smoking (38), physical activity (39), BMI (40), and triglycerides (41).

#### Linkage disequilibrium score (LDSC) regression analysis

For identifying complex human traits’ genetic frameworks, LDSC regression analysis is reliable and effective (42). It estimates disease heritability and examines genetic connections using GWAS summary data. In this study, we examined the genetic correlations of causative traits using GWAS summary data. The researchers constructed the LD reference panel from 1,000 Genomes^1^ with European LD scores for our investigation.

## Results

### Univariable MR results

#### Insomnia

**Figure 2:**
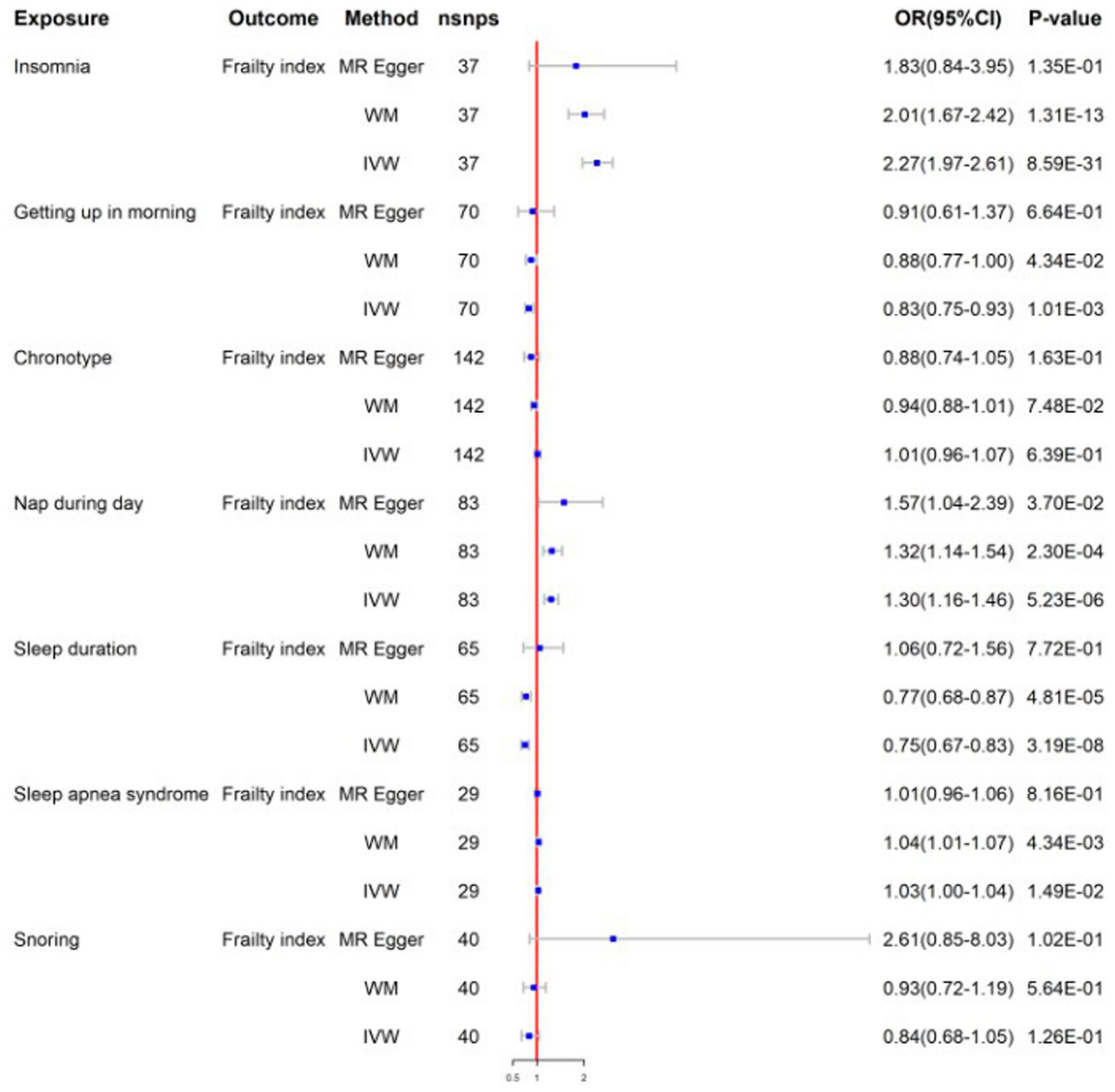
Mendelian randomization for sleep traits on frailty index; WM, weighted median; IVW, inverse variance weighted; nsnps, number of SNPs used in MR; OR, odds ratio; CI, confidence interval.

**Figure 3:**
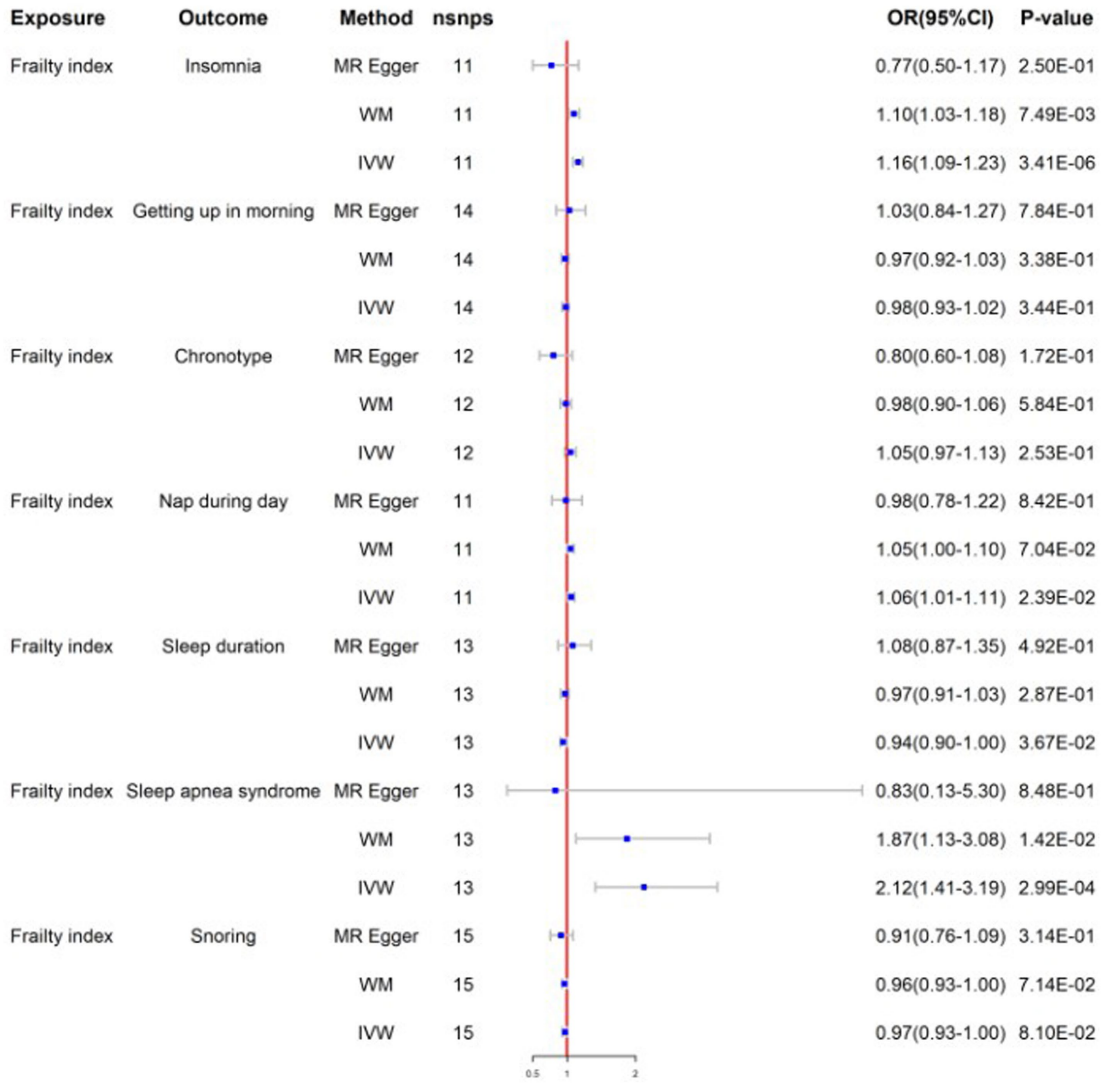
Mendelian randomization for frailty index on sleep traits; WM, weighted median; IVW, inverse variance weighted; nsnps, number of SNPs used in MR; OR, odds ratio; CI, confidence interval.

After screens and outlier elimination, we selected 37 SNPs substantially linked with insomnia for our MR analysis. IVW analysis showed that insomnia and FI are causally related (IVW, OR = 2.27, 95% CI = 1.97–2.61, *p* = 8.59E−13) (Figure 2), and other approaches supported this. No horizontal pleiotropy was detected in the MR-Egger regression test (*2301*). The results of the Cochrane Q test showed that there was heterogeneity (*Q* = 55.48, *p*<0.05). In reverse MR analysis, FI was also positively linked with insomnia (IVW: OR = 1.16, 95% CI = 1.09–1.23, *p* = 3.41E-06) (Figure 3). The sensitivity analysis of the reverse MR demonstrated heterogeneity and no pleiotropy. All MR results are in Supplementary Tables 1, 2.

#### Getting up in the morning

After screening, the MR analysis contained 109 SNPs. Getting up in the morning and FI were found to be negatively correlated by the IVW analysis results (IVW, OR = 0.83; 95% CI = 0.75–0.93, *p* = 1.01E-03) (Figure 2). No horizontal pleiotropy was found between them using the MR-Egger regression approach (*p*>0.05). To identify variability among SNPs, a heterogeneity test was run. The results remained heterogeneous even after the outliers were eliminated (*p* < 0.05). The reverse MR analysis did not provide significance (IVW, OR = 0.98; 95% CI = 0.93–1.02, *p* = 3.44E-01) (Figure 3). All MR results are in Supplementary Tables 3, 4.

#### Chronotype

The chronotype-FI relationship was studied using 142 SNPs. None of the MR estimates showed a chronotype-FI causal link (IVW, OR = 1.01, 95%CI = 0.96–1.07, *p* = 6.39E-01) (Figure 2). MR-Egger intercept testing indicated no horizontal pleiotropy (*P*>0.05). The chronotypeexhibited significant heterogeneity (*Q* = 302, *p* < 0.05), even after removing outlier SNPs. In the reverse study, FI had no genetic effect on chronotype risk (IVW, OR = 1.05, 95%CI =0.97–1.13, *p* = 2.53E-01) (Figure 3). All MR results are in Supplementary Tables 5, 6.

#### Napping during the day

We chose 83 SNPs as IVs and found that daytime nap and FI were strongly linked (IVW: OR = 1.30, 95%CI = 1.16–1.46, *p* = 5.23E-06) (Figure 2). The heterogeneity analysis revealed that SNPs are associated with additional exposure factors, and the pleiotropy analysis revealed no horizontal pleiotropy. We utilized MR-PRESSO to remove outliers, which left the MR data positively correlated but the heterogeneity intact. The reverse MR analysis also yielded good findings (IVW: OR = 1.06, 95%CI = 1.01–1.11, *p* = 2.39E-02) (Figure 3). The sensitivity examination showed heterogeneity without pleiotropy. All MR results were in Supplementary Tables 7, 8.

#### Sleep duration

We obtained 65 SNPs associated with sleep duration. Sleep duration was found to have a negative causal relationship with FI based on the results of a two-sample MR study (IVW: OR = 0.75, 95%CI = 0.67–0.83, *p* = 3.19E-08) (Figure 2). No horizontal pleiotropy in sleep duration was identified through the application of the MR-Egger regression test, but the results are heterogeneous. In the inverse MR analysis, we obtained positive results without heterogeneity or pleiotropy (IVW: OR = 0.94, 95%CI = 0.90–1.00, *p* = 3.67E-02) (Figure 3). Bidirectional MR analyses both suggested a bidirectional negative correlation causality between sleep duration and FI. All MR results are in Supplementary Tables 9, 10.

#### Sleep apnea syndrome

Following stringent criteria for SNP exclusion, we employed 29 SNPs to diagnose sleep apnea syndrome. In particular, our research shows a significant correlation between FI and sleep apnea syndrome (IVW: OR = 1.03, 95%CI = 1.00–1.05, *p* = 1.49E-02) (Figure 2). Moreover, utilizing the MR-Egger intercept and Cochran’s Q test in our analysis, we were unable to find any evidence of heterogeneity or pleiotropy of effects. The reverse MR analysis produced similar findings (IVW: OR = 2.12, 95%CI = 1.41–3.19, *p* = 2.99E-04) (Figure 3). Similar to reverse MR analyses, sensitivity analyses failed to identify heterogeneity or pleiotropy. All MR results were in Supplementary Tables 11, 12.

#### Snoring

There was insufficient evidence of statistical significance in these MR results, which showed that snoring was associated with FI (IVW: OR = 0.84, 95%CI = 0.68–1.05, *p* = 1.26E-01) (Figure 2). The findings of the reverse MR are the same (IVW: OR = 0.97, 95%CI = 0.93–1.00, *p* = 8.10E-02) (Figure 3). All MR results were in Supplementary Tables 13, 14.

The results of all sensitivity analyses can be seen in Table 2. Additionally, we used scatter plots, funnel plots, leave-one-out plots, and forest plots (Supplementary materials) to illustrate the study results.

**Table 2:** Sensitivity analysis of the associations between sleep traits and frailty index.

Exposures	Outcomes	Heterogeneity test				Pleiotropy test	
		MR-Egger		IVW		MR-Egger intercept	
		*Q*	pval	*Q*	pval	Intercept	*p*
Insomnia	Frailty index	54.99	1.70E-02	55.48	2.00E-02	2.34E-03	5.79E-01
Getting up in morning	Frailty index	142.63	3.21E-07	143.08	4.17E-07	−1.18E-03	6.43E-01
Chronotype	Frailty index	296.36	2.92E-13	302.04	9.19E-14	2.43E-03	1.03E-01
Nap during day	Frailty index	141.99	3.31E-05	143.45	3.20E-05	−1.84E-03	3.64E-01
Sleep duration	Frailty index	112.04	1.42E-04	118.11	4.52E-05	−4.32E-03	6.95E-02
Sleep apnea syndrome	Frailty index	31.30	2.59E-01	32.05	2.72E-01	1.93E-03	4.27E-01
Snoring	Frailty index	65.88	3.34E-03	8.42	8.12E-04	−8.25E-03	5.18E-02
Frailty index	Insomnia	13.60	1.37E-01	19.19	3.79E-02	8.93E-03	8.66E-02
Frailty index	Getting up in morning	17.18	1.43E-01	17.54	1.76E-01	−1.21E-03	6.24E-01
Frailty index	Chronotype	17.11	7.19E-02	22.78	1.90E-02	6.43E-03	9.88E-02
Frailty index	Nap during day	21.36	1.12E-02	22.64	1.22E-02	1.98E-03	4.81E-01
Frailty index	Sleep duration	18.03	8.09E-02	20.60	5.65E-02	−3.20E-03	2.36E-01
Frailty index	Sleep apnea syndrome	15.21	1.73E-01	16.64	1.64E-01	2.09E-02	3.31E-01
Frailty index	Snoring	34.57	9.85E-04	35.80	1.12E-03	1.39E-03	5.09E-01

### Multivariable MR results

Multivariate MR analyses adjusted for confounders were consistent with the results of univariate MR analyses (Table 3). This indicates that our findings have independent causal consequences.

**Table 3:** Multivariable MR results for the causal association of seven sleep traits with FI.

Exposure	Adjustment	Outcome	Beta	OR (95%CI)	*p*
Insomnia	BMI, physical activity, triglycerides, current tobacco smoking	Frailty index	0.64	1.89(1.64–2.19)	6.78E-18
Getting up in morning	BMI, physical activity, triglycerides, current tobacco smoking	Frailty index	−0.20	0.82(0.72–0.93)	1.40E-03
Chronotype	BMI, physical activity, triglycerides, current tobacco smoking	Frailty index	0.07	1.08(1.00–1.16)	5.50E-02
Nap during day	BMI, physical activity, triglycerides, current tobacco smoking	Frailty index	0.25	1.29(1.12–1.48)	3.01E-04
Sleep duration	BMI, physical activity, triglycerides, current tobacco smoking	Frailty index	−0.20	0.81(0.72–0.93)	2.04E-03
Sleep apnea syndrome	BMI, physical activity, triglycerides, current tobacco smoking	Frailty index	0.04	1.04(1.01–1.07)	9.65E-03
Snoring	BMI, physical activity, triglycerides, current tobacco smoking	Frailty index	0.53	1.70(1.39–2.07)	1.54E-07

### MRlap analysis

Table 4 presents the MRlap analysis’s findings. The results of MRlap suggest that the MR results of snoring on frailty are affected by sample overlap, which biases these results and makes them unreliable. We therefore used the corrected results. The remaining data corrected by MRlap were consistent with the results obtained by primary MR analysis, which confirms that the IVW method is robust.

**Table 4:** Results of MRlap analysis.

Exposure	Outcome	Corrected_effect	Corrected_effect_*p*	Difference_*p*
Insomnia	Frailty index	6.43E-01	5.71E-21	1.75E-05
Getting up in morning		−1.09E-01	3.32E-02	2.94E-02
Chronotype		−5.65E-03	8.50E-01	6.05E-03
Nap during day		7.94E-02	5.28E-02	5.11E-02
Sleep duration		−2.19E-01	2.29E-04	6.62E-02
Sleep apnea syndrome		4.59E-01	2.21E-01	2.79E-01
Snoring		−1.94E-01	2.13E-03	4.36E-02
Frailty index	Insomnia	2.24E-01	3.74E-03	1.76E-01
	Getting up in morning	−1.01E-01	3.39E-02	8.23E-01
	Chronotype	7.25E-02	3.99E-01	6.75E-01
	Nap during day	7.89E-02	3.07E-01	8.89E-01
	Sleep duration	−1.09E-01	1.05E-01	3.13E-01
	Sleep apnea syndrome	9.87E-02	1.67E-02	4.28E-02
	Snoring	−5.81E-02	3.14E-01	5.86E-01

Corrected_effect, corrected causal effect estimate; Corrected_effect_*p*, corrected causal effect *p*-value; Difference_*p*, difference *p*-value between observed and corrected effects.

### Genetic connection between causal traits

All sleep characteristics showed no genetic correlation except for chronotype (Rg = −0.03, *p* = 1.10E-02). All LDSC results were in Supplementary Table 15.

## Discussion

We examined the impact of seven sleep characteristics—chronotype, insomnia, daytime naps, length of sleep, morning awakenings, sleep apnea syndrome, and snoring—on FI in this MR investigation. To the best of our knowledge, this is the initial investigation that explores the causal connection between sleep traits and frailty.

We deduced from our MR research that insomnia, napping during the day, and sleep apnea syndrome increased the risk of frailty. Awakening in the morning, snoring and sleep duration decreased the risk of frailty. In reverse MR analysis, frailty is related to an increased risk of insomnia, napping during the day and sleep apnea syndrome. And it reduced the risk of sleep duration. There was no confirmed causal link between chronotype and frailty. After adjusting for other confounders, the MVMR study was consistent with the results of the UVMR study, suggesting independent causal effects between these traits. According to the LDSC regression, we only discovered a significant genetic association between chronotype and FI.

In recent years, an increasing body of observational research has indicated that sleep disorders are a risk factor for frailty progression. Sleep difficulties can indicate a variety of diseases, including poor health, co-morbidities including cardiovascular illness (43), depressive symptoms (44), cognitive dysfunction (45), and functional limitations (46), all of which disrupt sleep and raise the chance of frailty.

The mechanisms behind the correlation between sleep problems and frailty are not well understood at this time. Possible mechanisms that may be involved include the existence of persistent inflammation, endocrine system disorders (e.g., cortisol dysregulation, testosterone disorders), etc. There is a reciprocal association between sleep problems and frailty. Sleep disorders impede the development process, resulting in muscle loss and eventual frailty. Conversely, frailty can also disrupt sleep owing to increased inflammation and other bodily secretions (14).

Chronic inflammation is the primary mechanism that modern researchers believe is linked to the development of sleep problems and frailty. Inflammation is one of the important pathophysiological changes that may be closely associated with frailty (47).

Sleep initiation and promotion have a high inflammatory component. Sleep disturbances have been linked to an increase in inflammatory markers such as IL-6 (48–51). Pro-inflammatory cytokines can alter important metabolic processes, which can lead to frailty indirectly or directly by increasing proteolysis (52). Previous research has shown a link between frailty and higher inflammatory markers such as IL-6, CRP, fibrinogen, and factor VIII, independent of other chronic disease states (53, 54). IL-1β has been linked to increased growth hormone-releasing hormone and improved non-rapid-eye-movement sleep in humans (55). The NHANES study found inflammation as a potential relationship between sleep problems and frailty, and anti-inflammatory diets can reduce the negative effects of poor sleep quality on frailty (56).

Low and lengthy sleep durations may increase the incidence of sarcopenia, according to a recent epidemiological study (57) sarcopenia reduces muscle atrophy, power, and coordination, which can cause functional limits and mobility issues (58) sarcopenia is critical to frailty (59). Frailty and sarcopenia share age-related body composition, inflammation, and endocrine variables as their main causes (60). Reduced insulin sensitivity, testosterone, growth hormone, and IGF-1 levels limit protein synthesis. In frail or sarcopenic older persons, obesity-derived intracellular lipotoxicity causes inflammation, oxidative stress, and insulin resistance (39). Sleep disturbances can affect sarcopenia and frailty through multiple routes. Thus, older people should prioritize high-quality, long-term sleep to prevent, manage, and treat frailty and reduce its effects. Abnormalities in biochemical processes, such as reduced endogenous testosterone levels (61, 62) and poorer renal function (63, 64), have been associated with sleep issues in men. Any or all of these alterations could explain why men with sleep difficulties have a higher risk of frailty. Sleep deprivation has been linked to lower testosterone levels, chronic inflammation, increased oxidative stress, and imbalanced growth hormone release (65, 66).

Women who experience sleep problems have reduced deep sleep, which increases cortisol secretion and decreases growth hormone release, which has a substantial impact on hormonal and metabolic functioning (65, 67). Longer durations of higher melatonin and cortisol levels, together with a drop in body temperature (68), are associated with biologically longer nights, which may weaken the immune system (69). Another reason for this could be that women typically have greater levels of interleukin-6 and C-reactive protein (CRP), two substances that serve as indicators in the genesis of frailty (70).

By comprehending the correlation between sleep disturbances and frailty, it is possible to proactively identify risk factors and apply appropriate interventions. For instance, older adults with sleep difficulties might receive regular evaluation and treatment to decrease the likelihood of frailty. Aside from addressing sleep difficulties, it is important to also focus on managing frailty, which involves providing nutritional assistance, engaging in exercise rehabilitation, and offering psychological support. Furthermore, implementing sleep education initiatives specifically targeted at older people can significantly influence their frailty status. The majority of medical services provided to older adult individuals who are physically weak do not include the evaluation or treatment of sleep disturbances. Considering the significance of geriatrics in relation to frailty, it is advisable for older people with insomnia to undergo a thorough geriatrics assessment. It may be more pertinent to assess whether treatments that improve sleep quality in older adult people have a positive impact on frailty outcomes.

The benefits of our study are: First, this is the first investigation into the genetic relationship between sleep characteristics and the likelihood of frailty. Biases might still exist even though some previous cross-sectional researches have corrected for confounding variables. Second, we employed genetic data from a substantial sample size, bolstering the dependability of our findings and mitigating the impact of confounders. MR analysis allows for quick and cost-effective causal relationship screening due to the vast array of known genetic relationships that are utilized to identify appropriate genetic instrumental variables. Finally, since genes are unlikely to be environmental confounders, the MR analysis uses them as IVs. Reverse causation has no effect on the genotype-disease relationships since genotypes are dispersed at random prior to exposure during pregnancy.

However, this study has some limitations. First, most observational studies of sleep problems and frailty involve older individuals. We could not analyze our findings for each age group of sleep or frailty patients because there is no enough GWAS database. Second, some of our data have overlapping problems due to database restrictions. So we evaluated the results using LDSC regression analysis, but found no genetic correlation except for the chronotype. Additionally, we employed MRlap to rectify estimation bias caused by sample overlap. Third, our study is European-only. Thus, more research is needed to discover if our work can be applied to other populations. Further, our study used self-reported sleep features rather than objective assessments, which may have introduced bias. Finally, our study did not examine the mediators between sleep characteristics and frailty, such as depression, inflammatory factors, and sarcopenia, which could help us understand the relationship. Future studies should consider these factors. Besides the foregoing, other factors may influence our research and cause deviation, requiring larger MR investigations or RCTs.

## Conclusion

To summarize, the MR analysis offers substantial support for the hypothesis that sleep characteristics and frailty are causally related. According to our research, getting up early and appropriate sleep duration may be preventive factors against frailty, which could lower the occurrence of frailty. Frailty may be predisposed to sleep apnea syndrome, insomnia, and naps during the day. Improving these elements could prevent us from becoming feeble. Our research offers fresh perspectives on the possible mechanisms underlying the occurrence and progression of frailty.

The study has clinical significance since sleep complaints can be used to identify people who are at risk for frailty. Additionally, by addressing sleep disorders appropriately, it may be possible to prevent, delay, or even reverse frailty.

## Data availability statement

The original contributions presented in the study are included in the article/Supplementary material, further inquiries can be directed to the corresponding author.

## Author contributions

ZD: Conceptualization, Data curation, Visualization, Writing – original draft, Writing – review & editing. YH: Data curation, Formal analysis, Writing – original draft. LD: Conceptualization, Data curation, Methodology, Visualization, Writing – original draft. ZB: Data curation, Formal analysis, Software, Writing – original draft. QH: Methodology, Resources, Writing – original draft. XF: Data curation, Formal analysis, Writing – original draft. HL: Methodology, Software, Writing – original draft. TH: Funding acquisition, Project administration, Supervision, Validation, Visualization, Writing – review & editing.

## Footnotes

^1^ https://github.com/bulik/ldsc
