# Supplementary figures and images for: Causality between sleep traits and the risk of frailty: a Mendelian randomization study
Source: Front Public Health. 2024 May 9;12:1381482. doi: 10.3389/fpubh.2024.1381482 (PMC11112029; doi:10.3389/fpubh.2024.1381482)

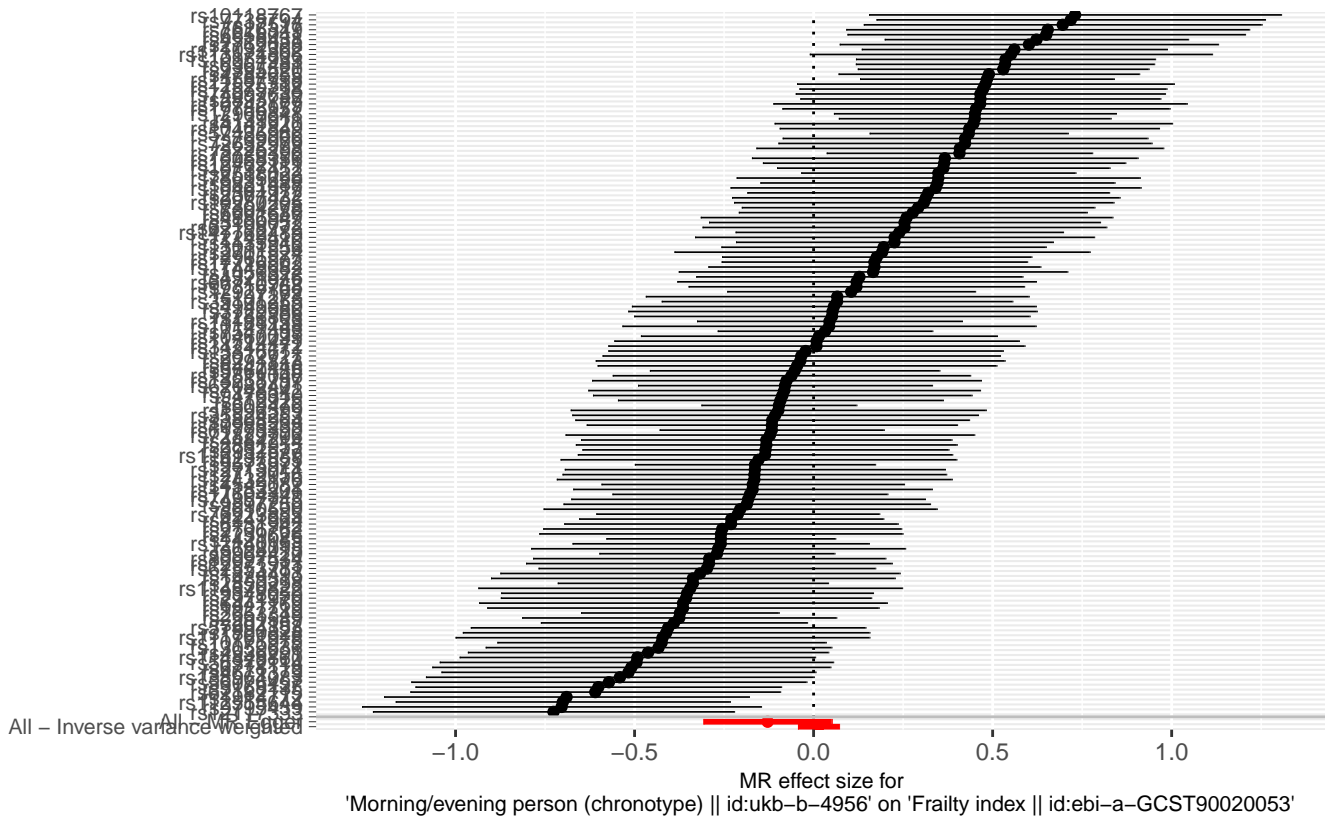

Supplement: Supplementary file 1 [file Data_Sheet_1.ZIP › Supplementary Materials/Chronotype/forward/Chronotype forward forest.pdf]

# MR Method

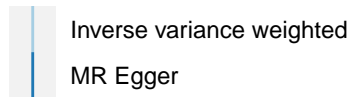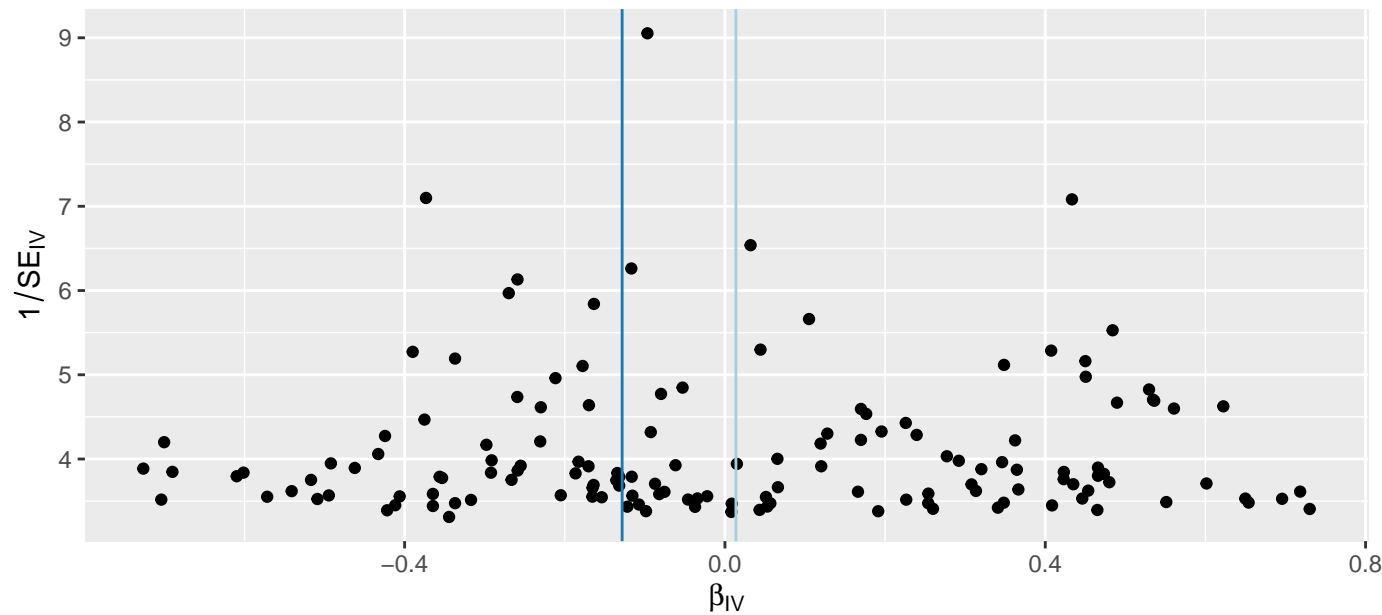

Supplement: Supplementary file 1 [file Data_Sheet_1.ZIP › Supplementary Materials/Chronotype/forward/Chronotype forward funnel.pdf]

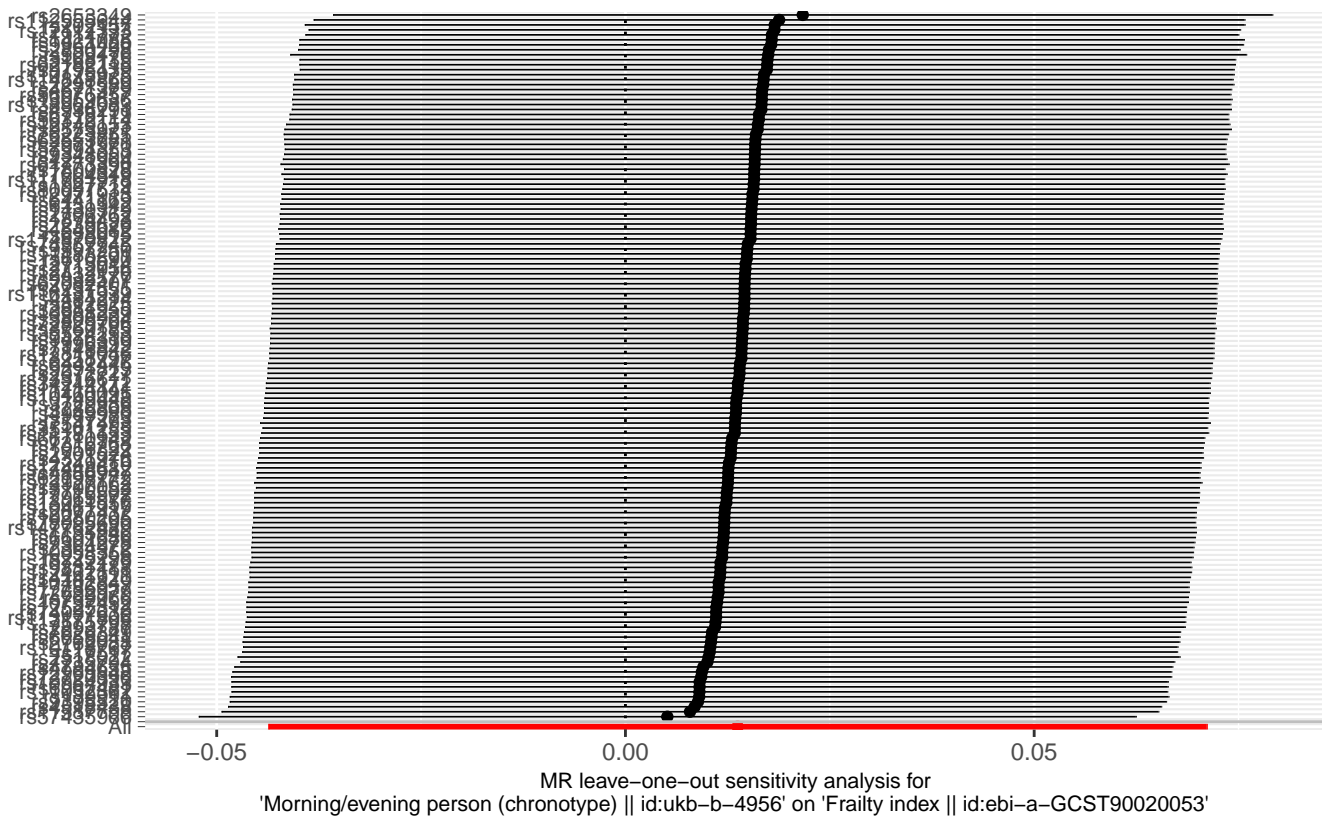

Supplement: Supplementary file 1 [file Data_Sheet_1.ZIP › Supplementary Materials/Chronotype/forward/Chronotype forward leave-one-out.pdf]

NP effect on Frailty index || id:ebi-a-GCST90020053

### MR Test

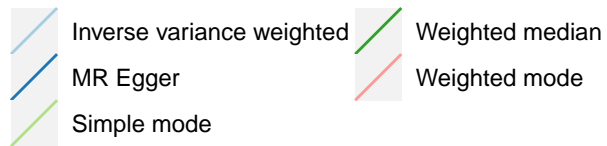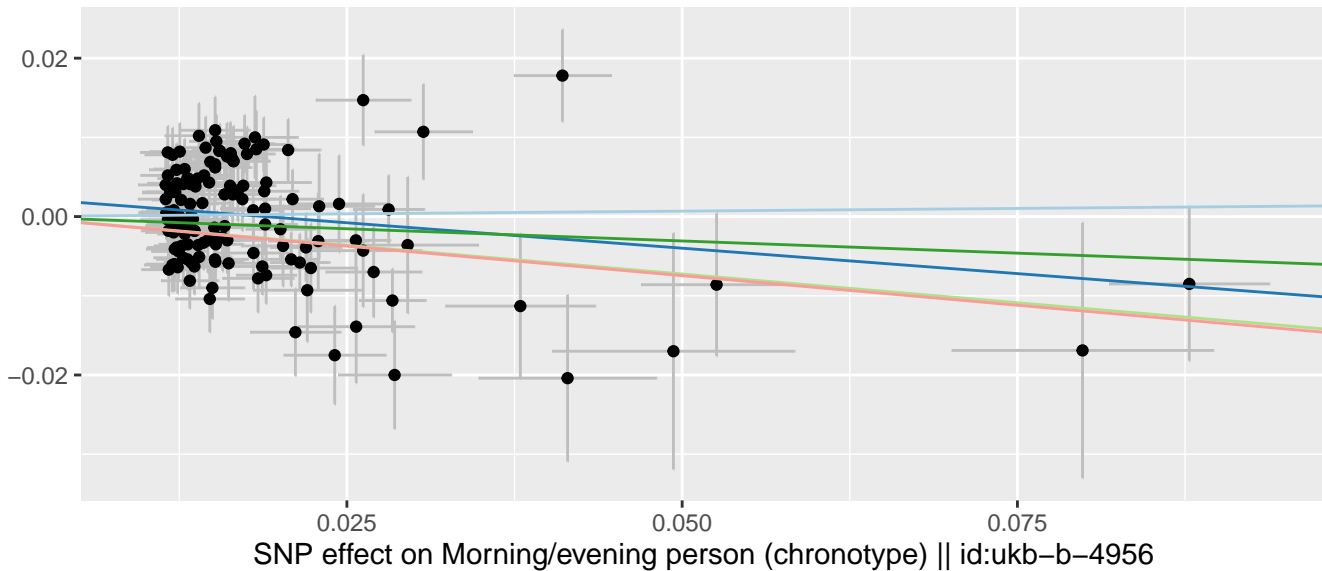

Supplement: Supplementary file 1 [file Data_Sheet_1.ZIP › Supplementary Materials/Chronotype/forward/Chronotype forward scatter.pdf]

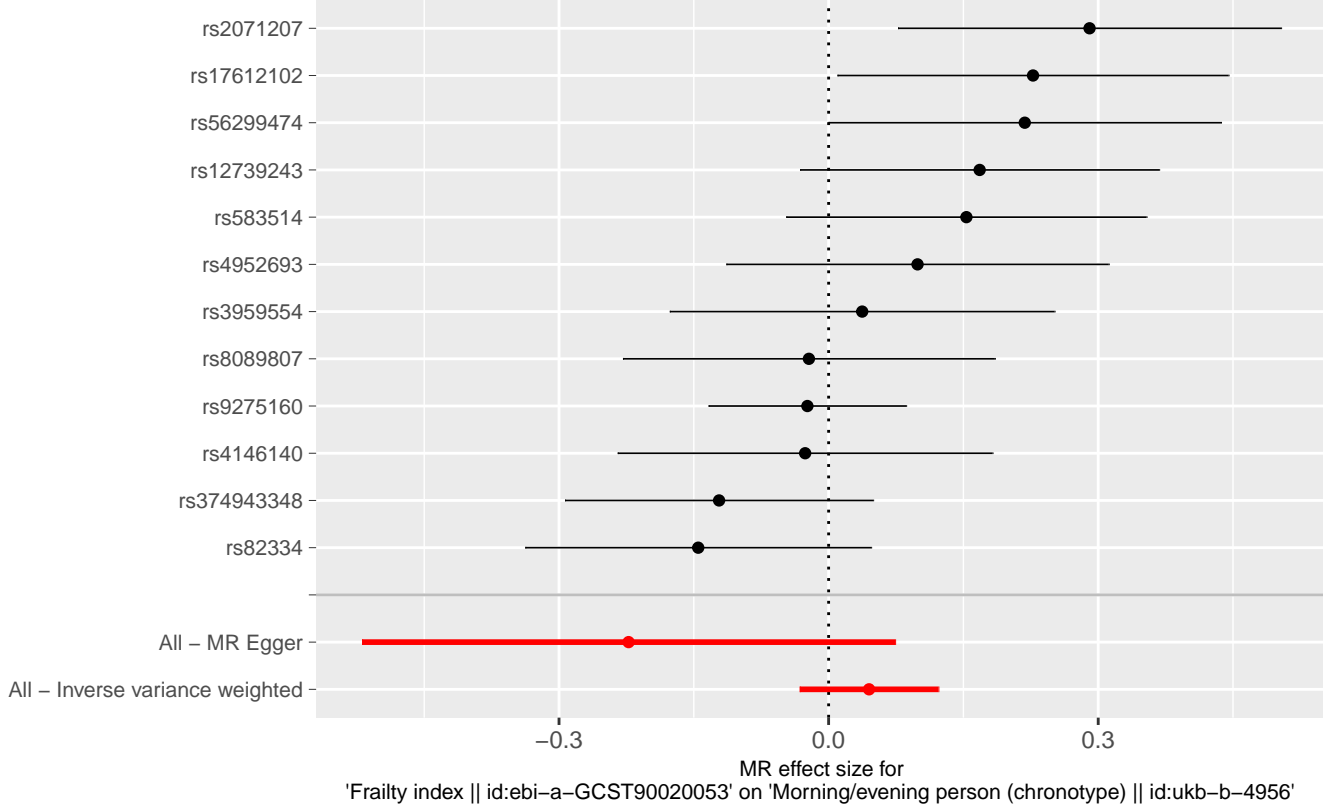

Supplement: Supplementary file 1 [file Data_Sheet_1.ZIP › Supplementary Materials/Chronotype/reverse/Chronotype reverse forest.pdf]

# MR Method

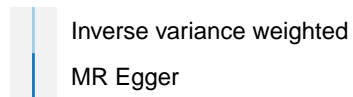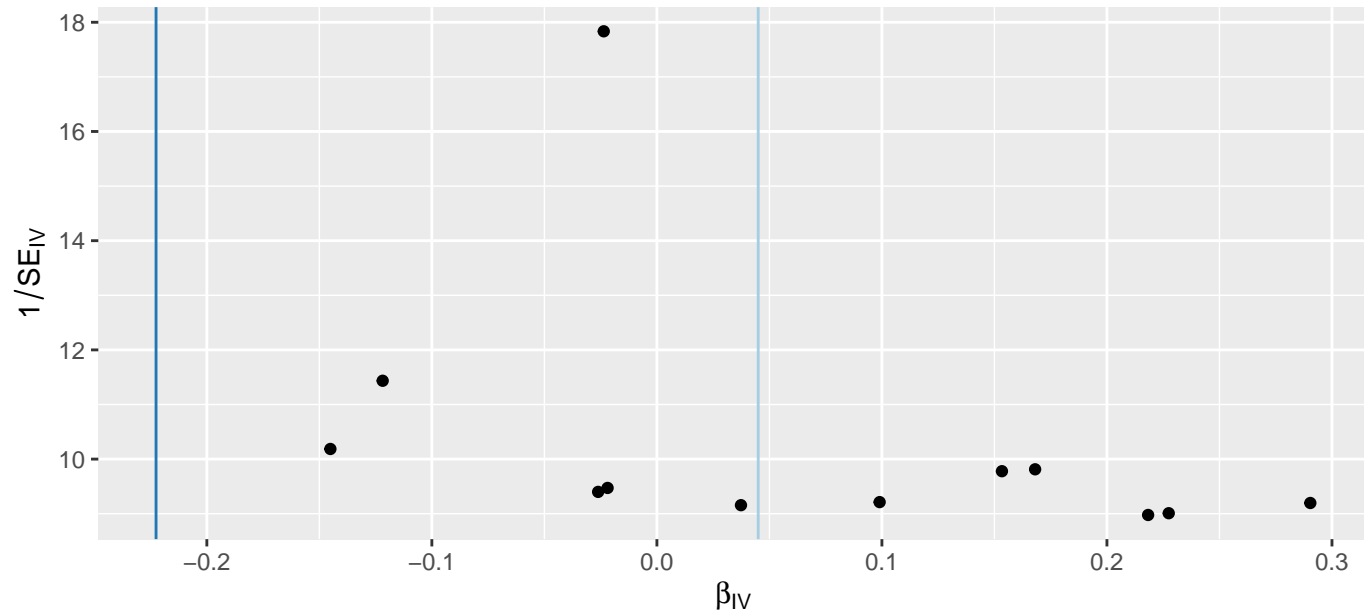

Supplement: Supplementary file 1 [file Data_Sheet_1.ZIP › Supplementary Materials/Chronotype/reverse/Chronotype reverse funnel.pdf]

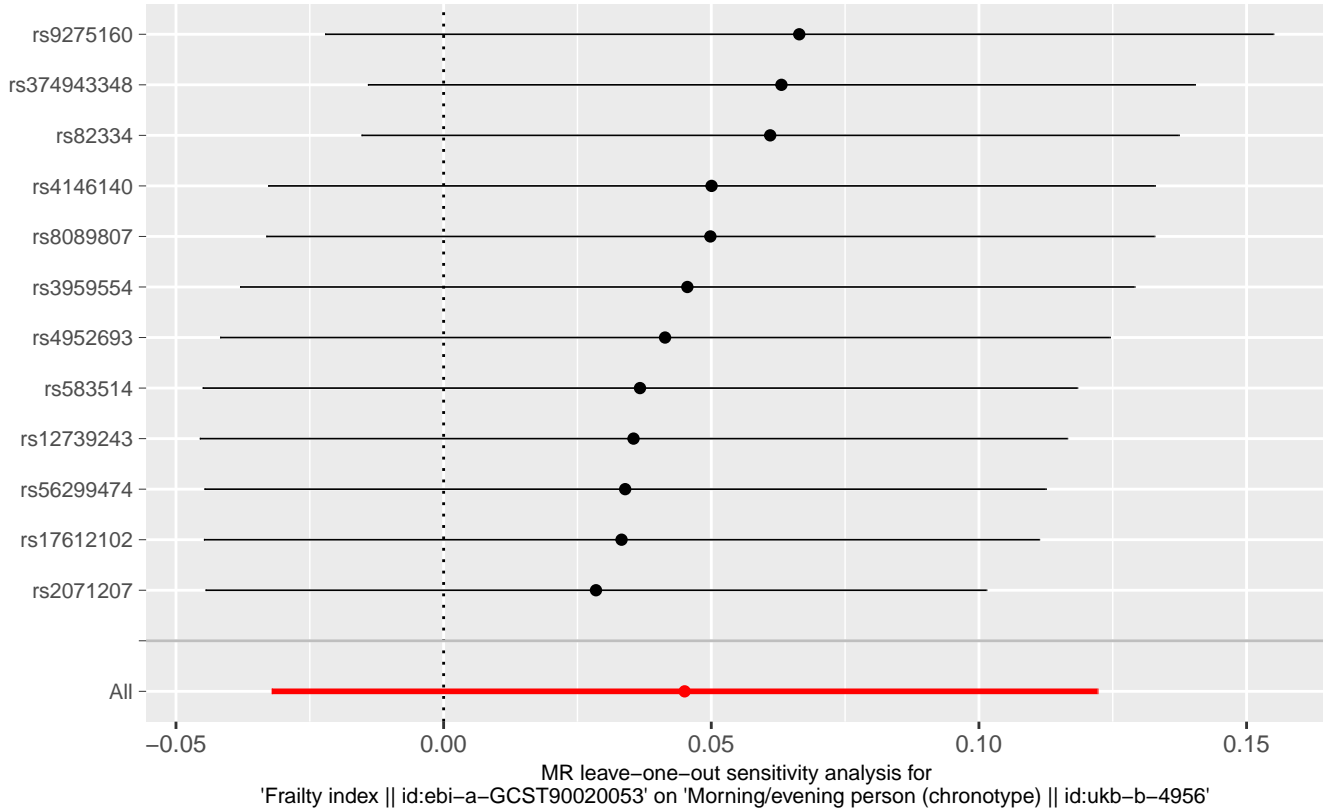

Supplement: Supplementary file 1 [file Data_Sheet_1.ZIP › Supplementary Materials/Chronotype/reverse/Chronotype reverse leave-one-out.pdf]

Effect on Morning/evening person (chronotype) || id:ukb-b-4956

### MR Test

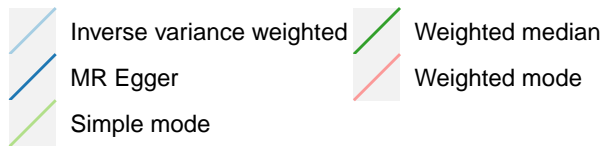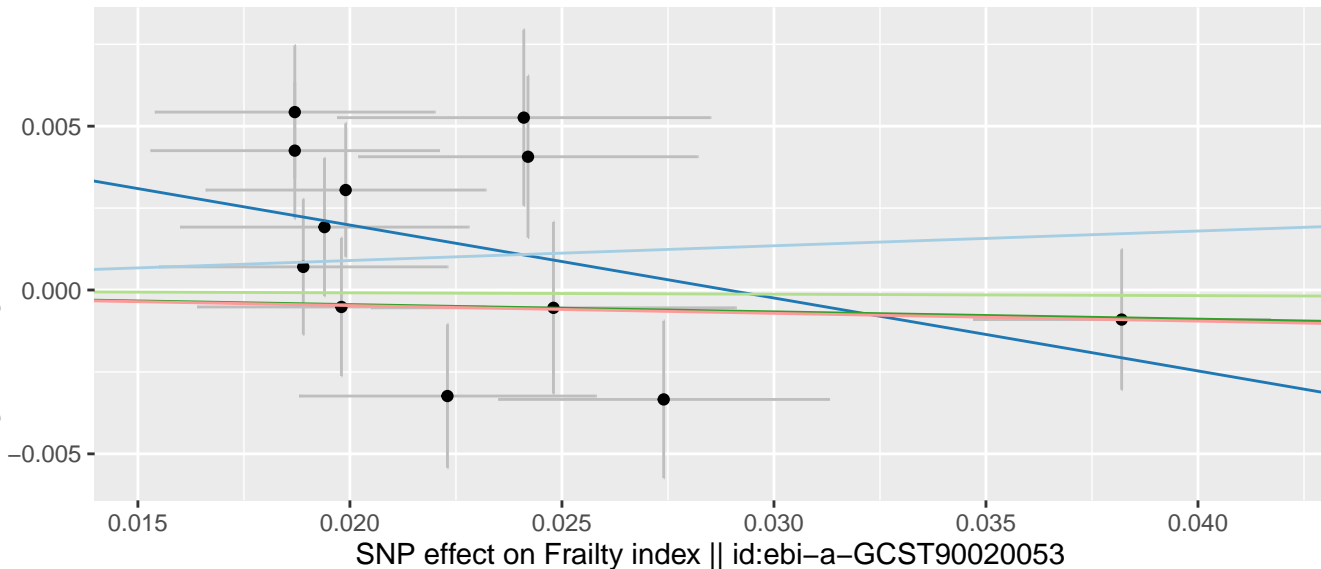

Supplement: Supplementary file 1 [file Data_Sheet_1.ZIP › Supplementary Materials/Chronotype/reverse/Chronotype reverse scatter.pdf]

All – Inverse variance weighted

All – MR Egger

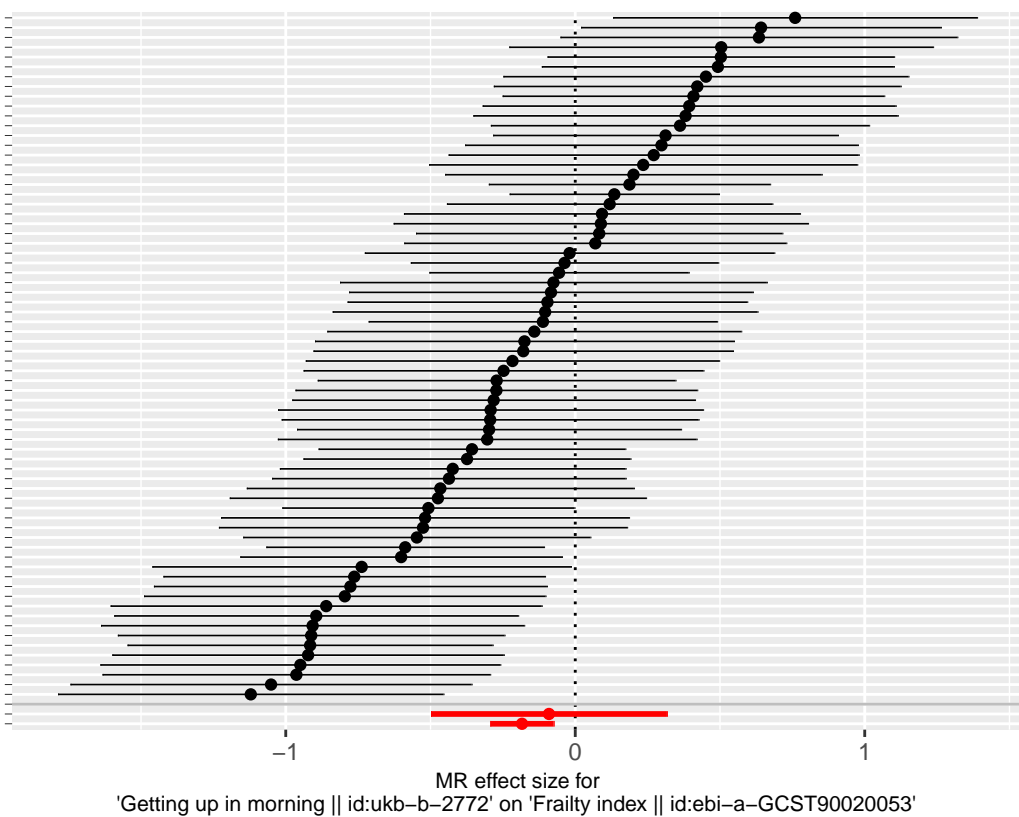

Supplement: Supplementary file 1 [file Data_Sheet_1.ZIP › Supplementary Materials/Getting up in morning/forward/Getting up in morning forward forest.pdf]

# MR Method

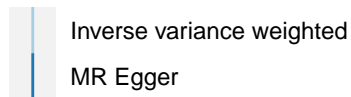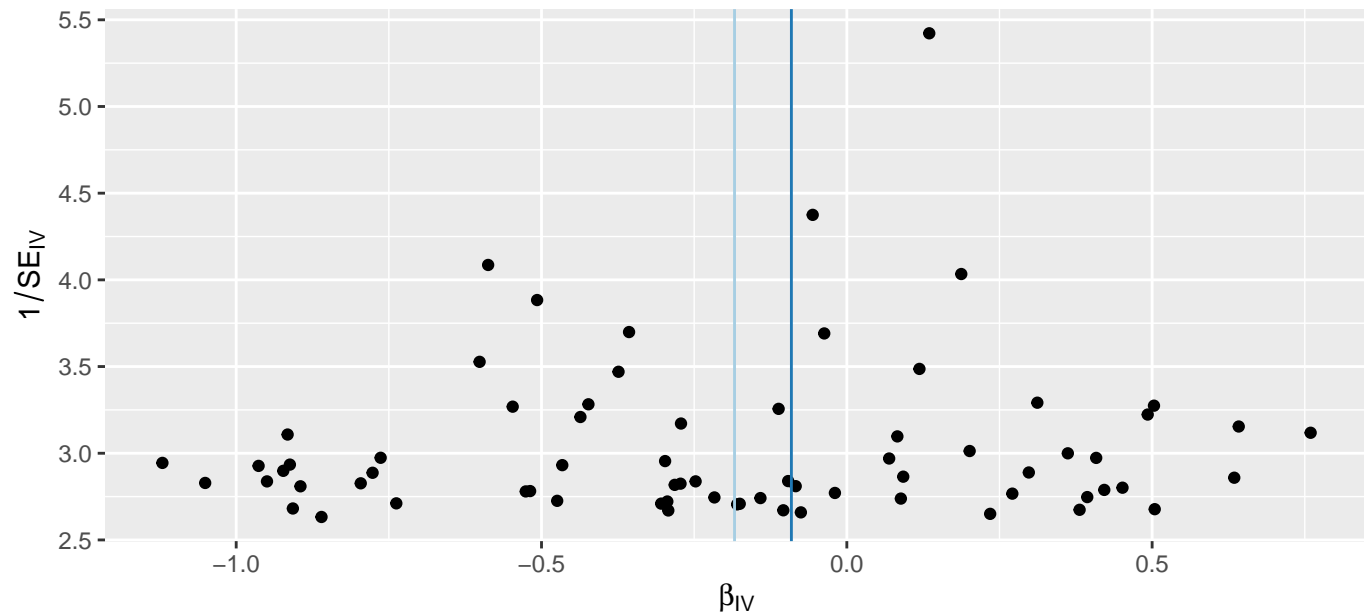

Supplement: Supplementary file 1 [file Data_Sheet_1.ZIP › Supplementary Materials/Getting up in morning/forward/Getting up in morning forward funnel.pdf]

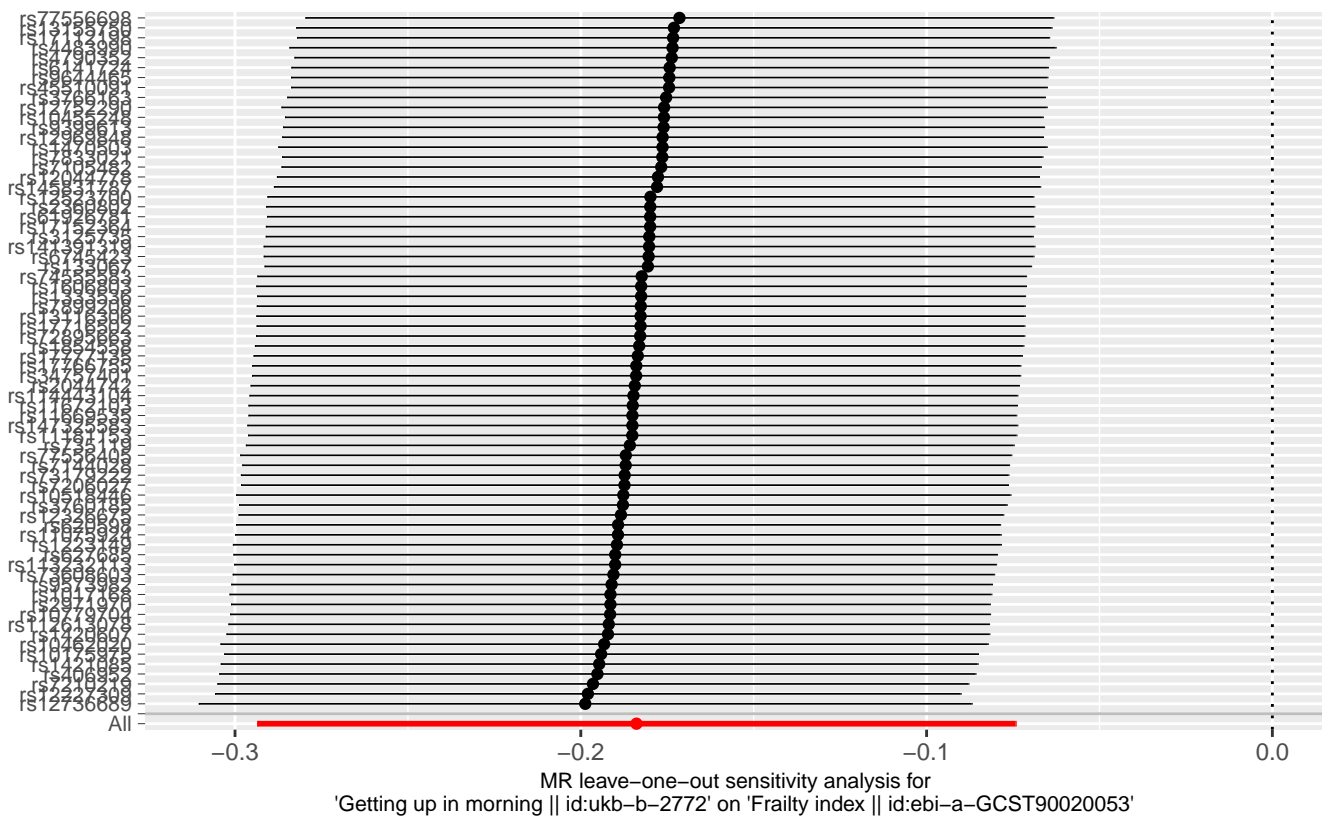

Supplement: Supplementary file 1 [file Data_Sheet_1.ZIP › Supplementary Materials/Getting up in morning/forward/Getting up in morning forward leave-one-out.pdf]

NP effect on Frailty index || id:ebi-a-GCST90020053

### MR Test

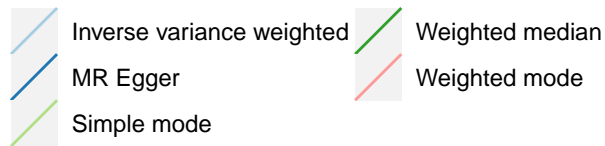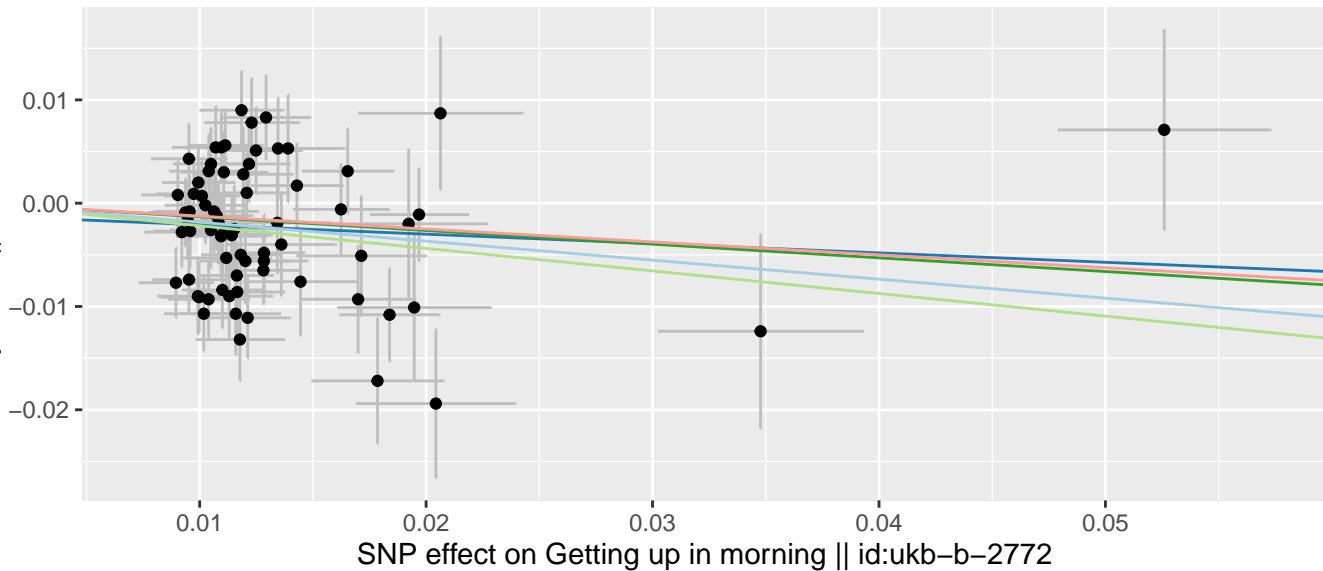

Supplement: Supplementary file 1 [file Data_Sheet_1.ZIP › Supplementary Materials/Getting up in morning/forward/Getting up in morning forward scatter.pdf]

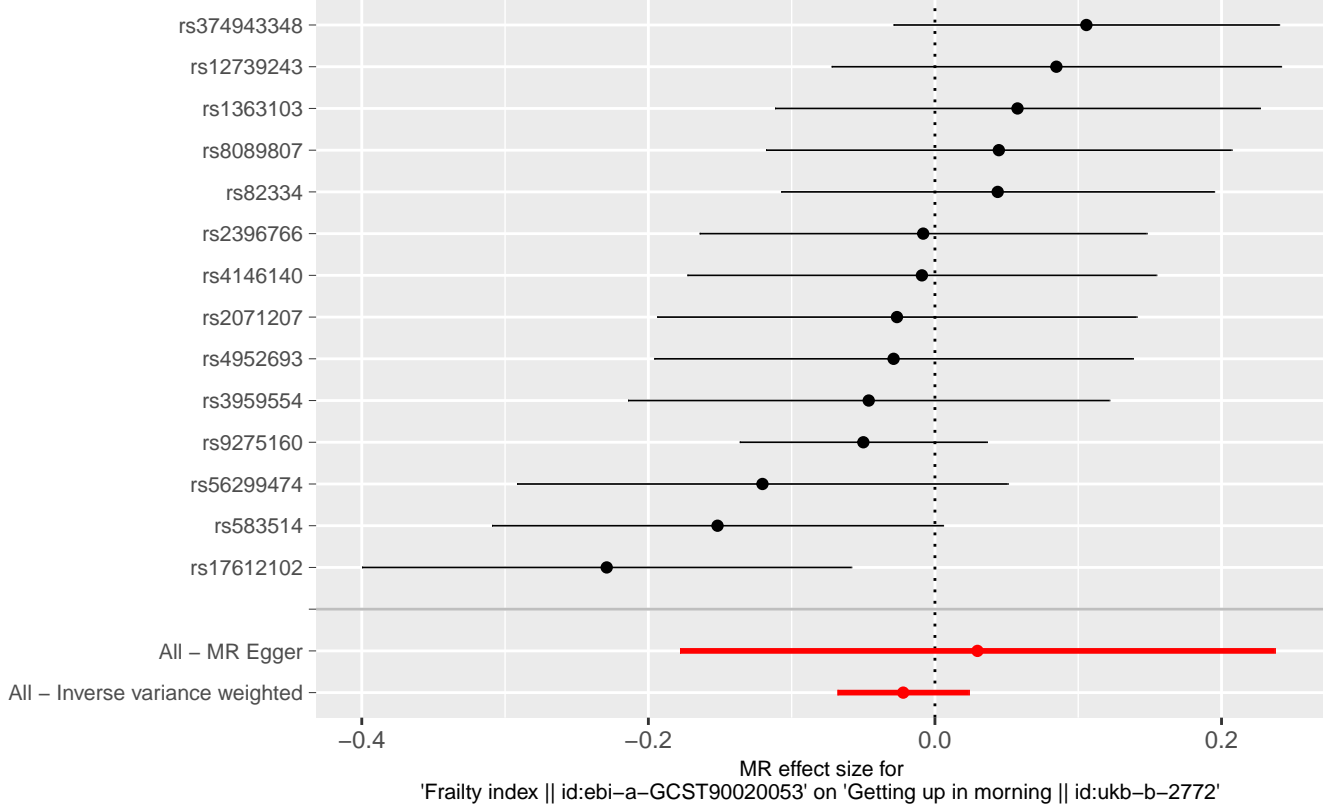

Supplement: Supplementary file 1 [file Data_Sheet_1.ZIP › Supplementary Materials/Getting up in morning/reverse/Getting up in morning reverse forest.pdf]

# MR Method

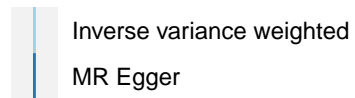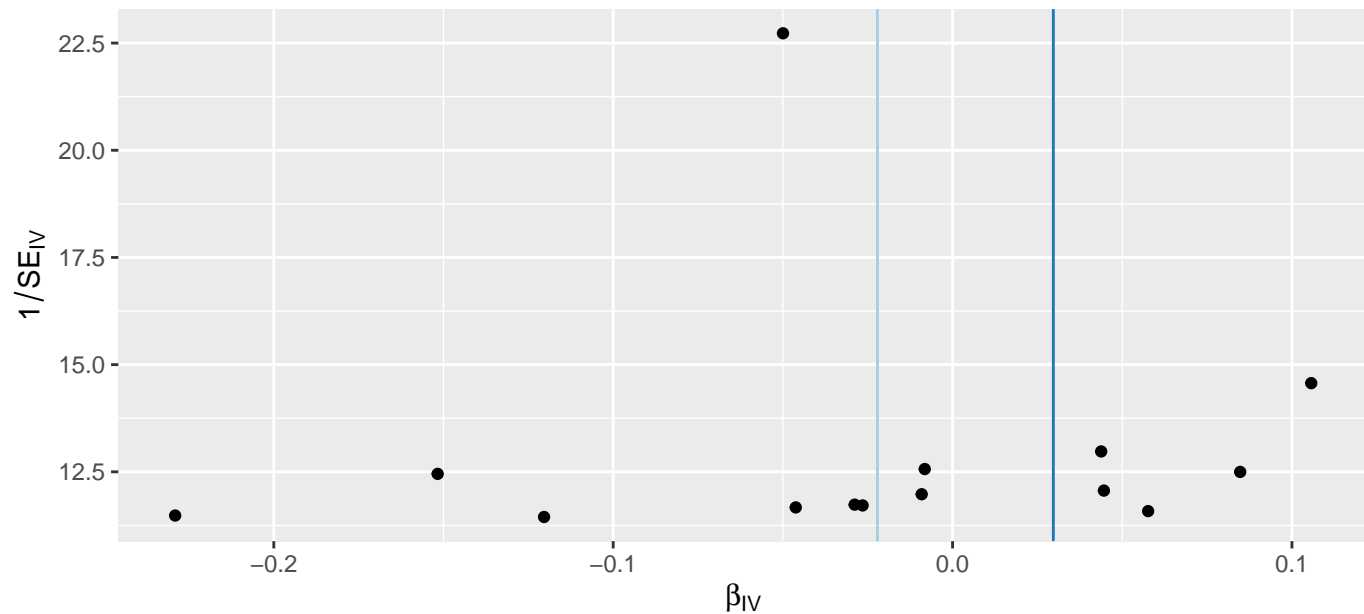

Supplement: Supplementary file 1 [file Data_Sheet_1.ZIP › Supplementary Materials/Getting up in morning/reverse/Getting up in morning reverse funnel.pdf]

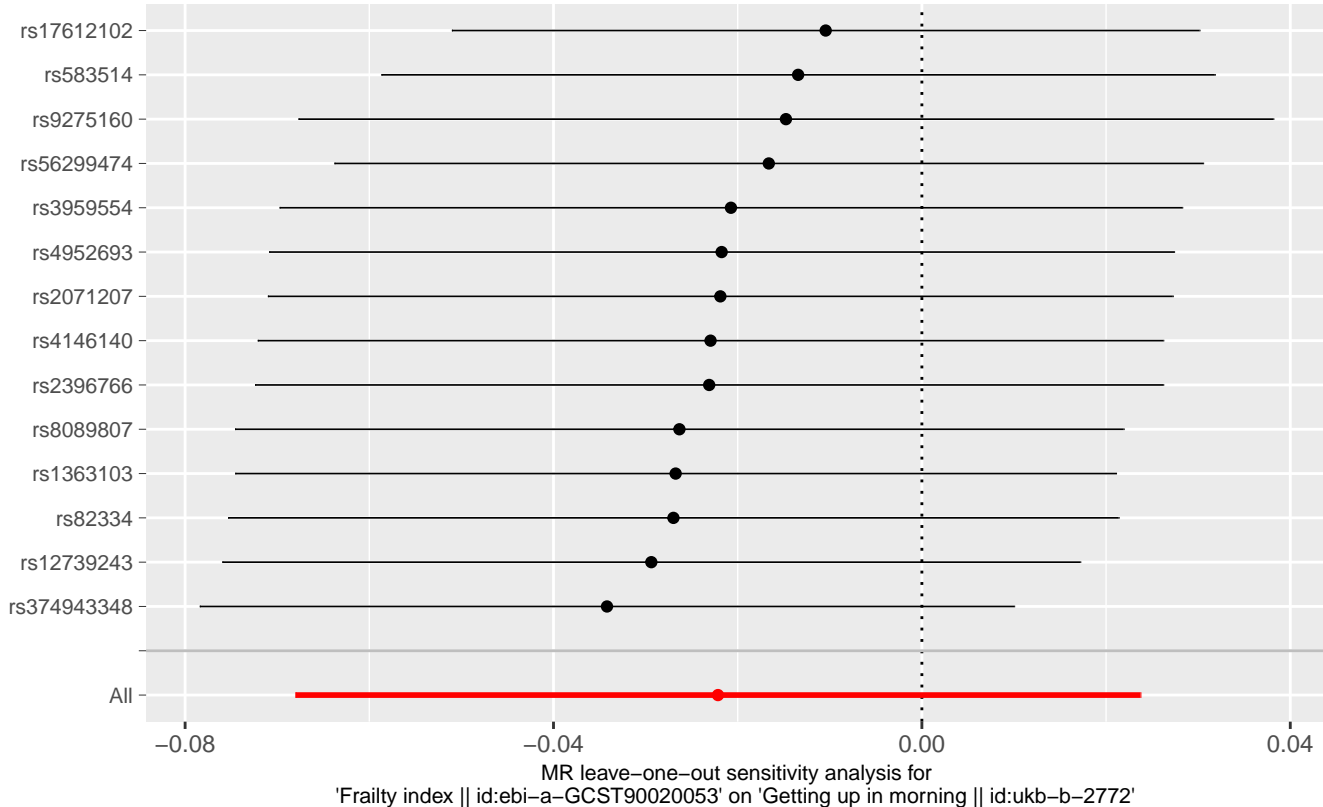

Supplement: Supplementary file 1 [file Data_Sheet_1.ZIP › Supplementary Materials/Getting up in morning/reverse/Getting up in morning reverse leave-one-out.pdf]

NP effect on Getting up in morning || id:ukb-b-2772

### MR Test

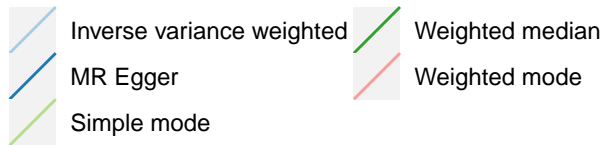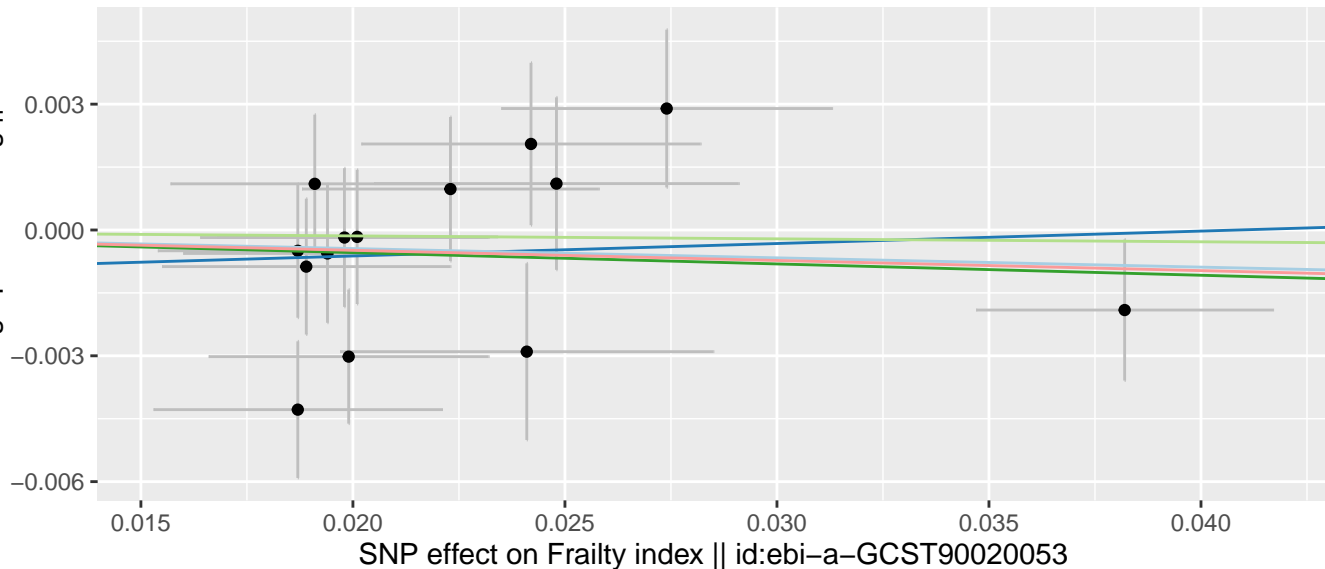

Supplement: Supplementary file 1 [file Data_Sheet_1.ZIP › Supplementary Materials/Getting up in morning/reverse/Getting up in morning reverse scatter.pdf]

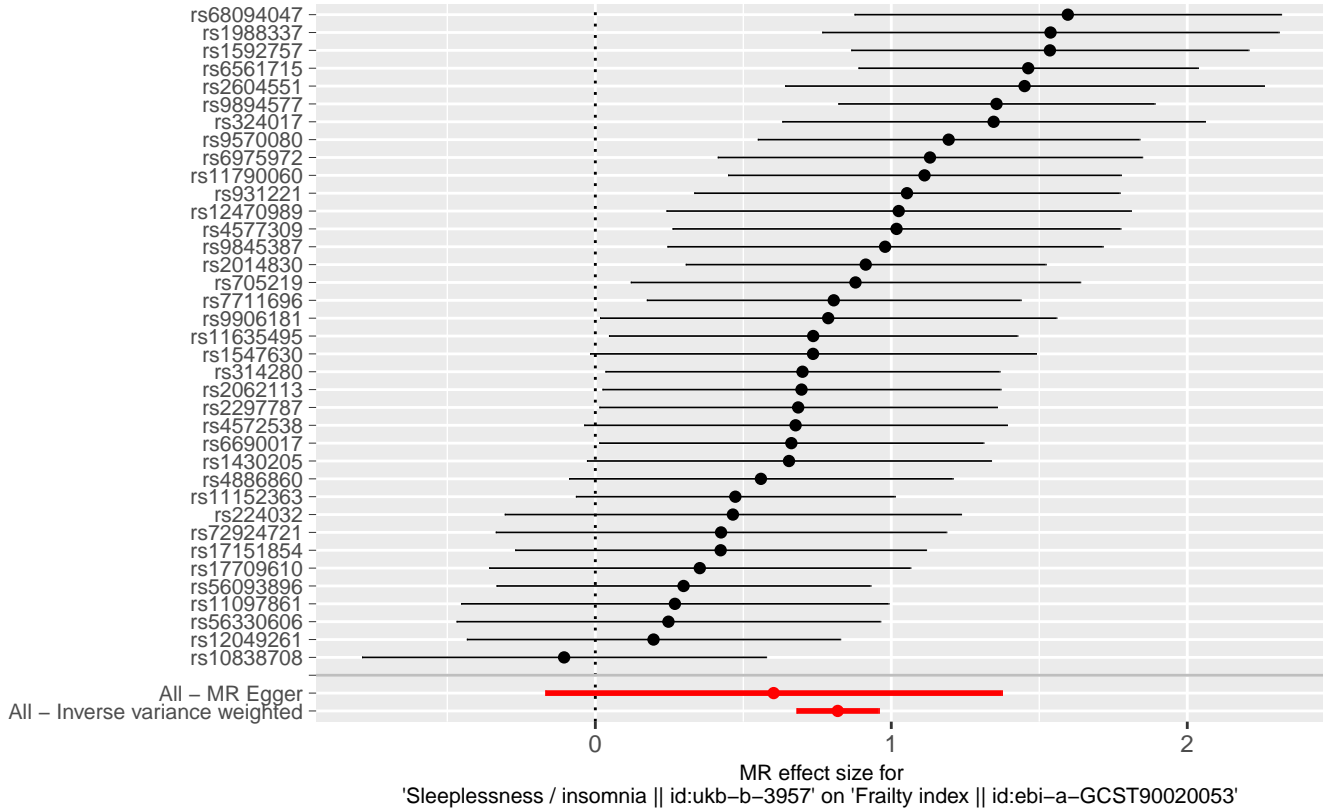

Supplement: Supplementary file 1 [file Data_Sheet_1.ZIP › Supplementary Materials/Insomnia/forward/Insomnia forward forest.pdf]

# MR Method

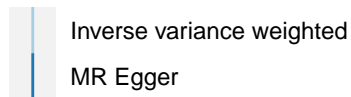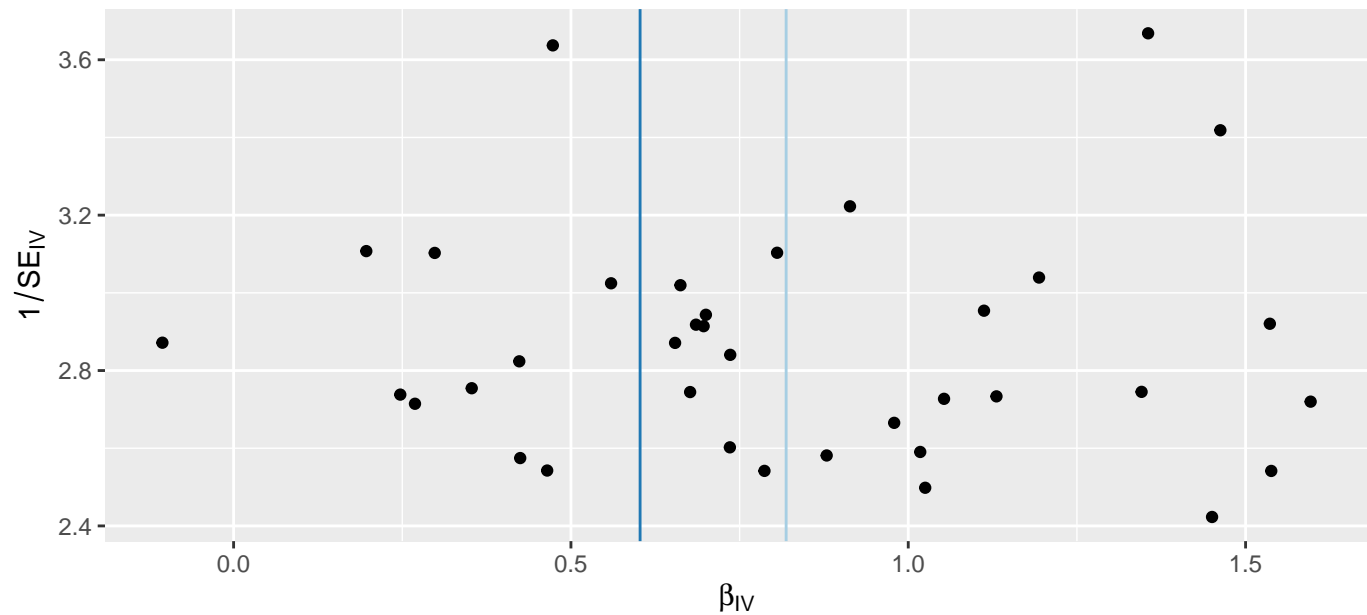

Supplement: Supplementary file 1 [file Data_Sheet_1.ZIP › Supplementary Materials/Insomnia/forward/Insomnia forward funnel.pdf]

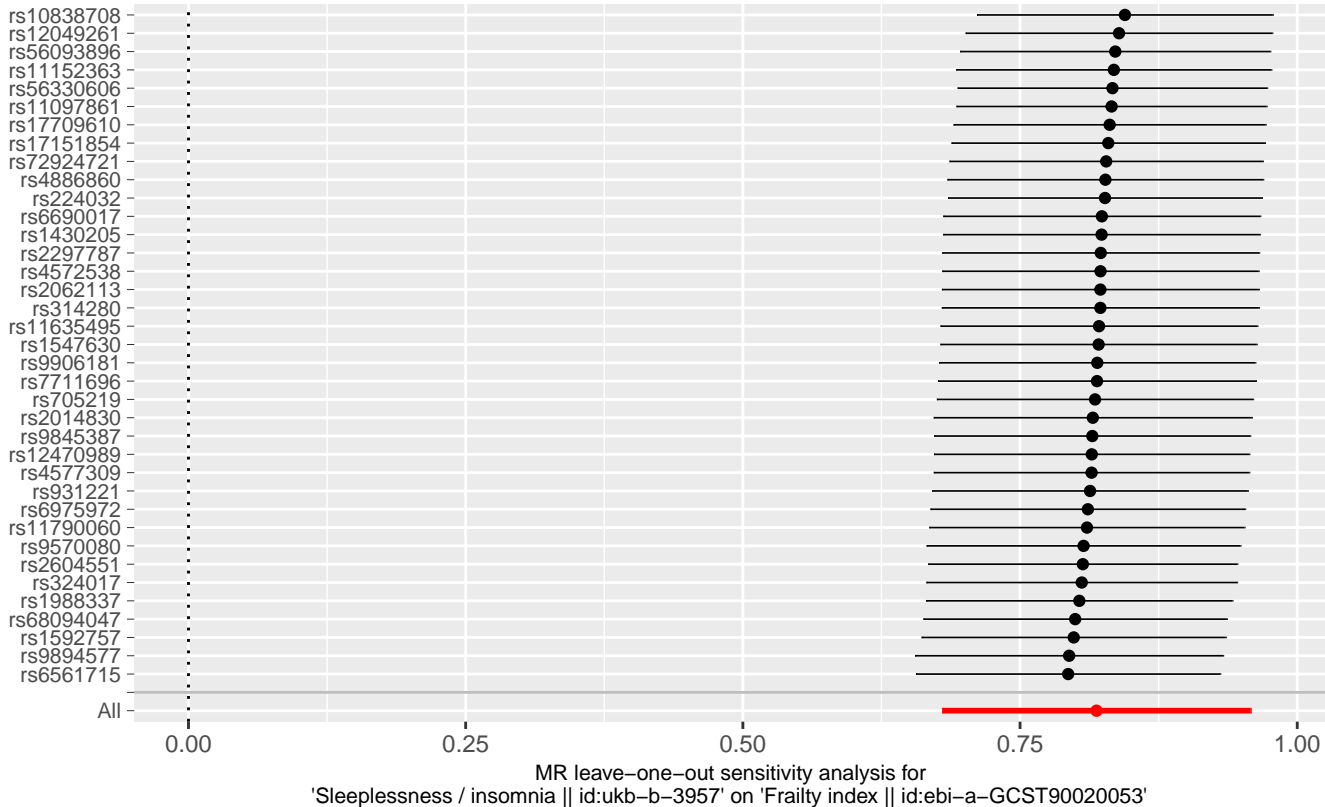

Supplement: Supplementary file 1 [file Data_Sheet_1.ZIP › Supplementary Materials/Insomnia/forward/Insomnia forward leave-one-out.pdf]

NP effect on Frailty index || id:ebi-a-GCST90020053

### MR Test

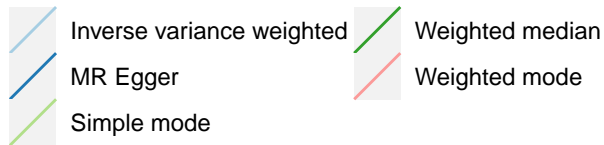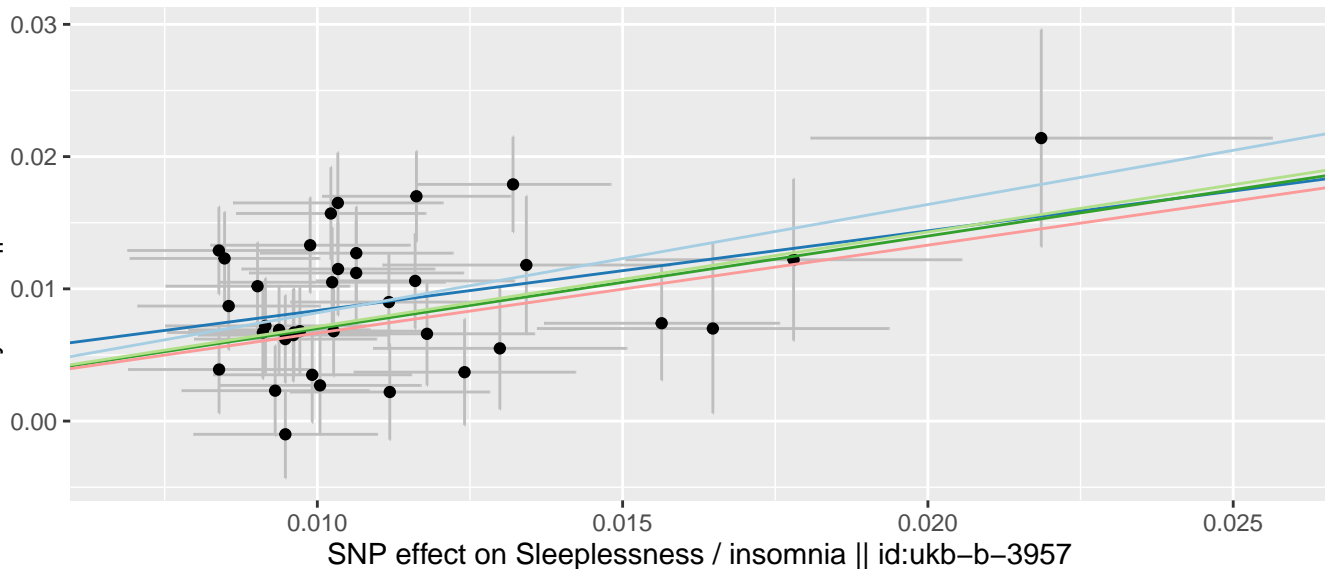

Supplement: Supplementary file 1 [file Data_Sheet_1.ZIP › Supplementary Materials/Insomnia/forward/scatter.pdf]

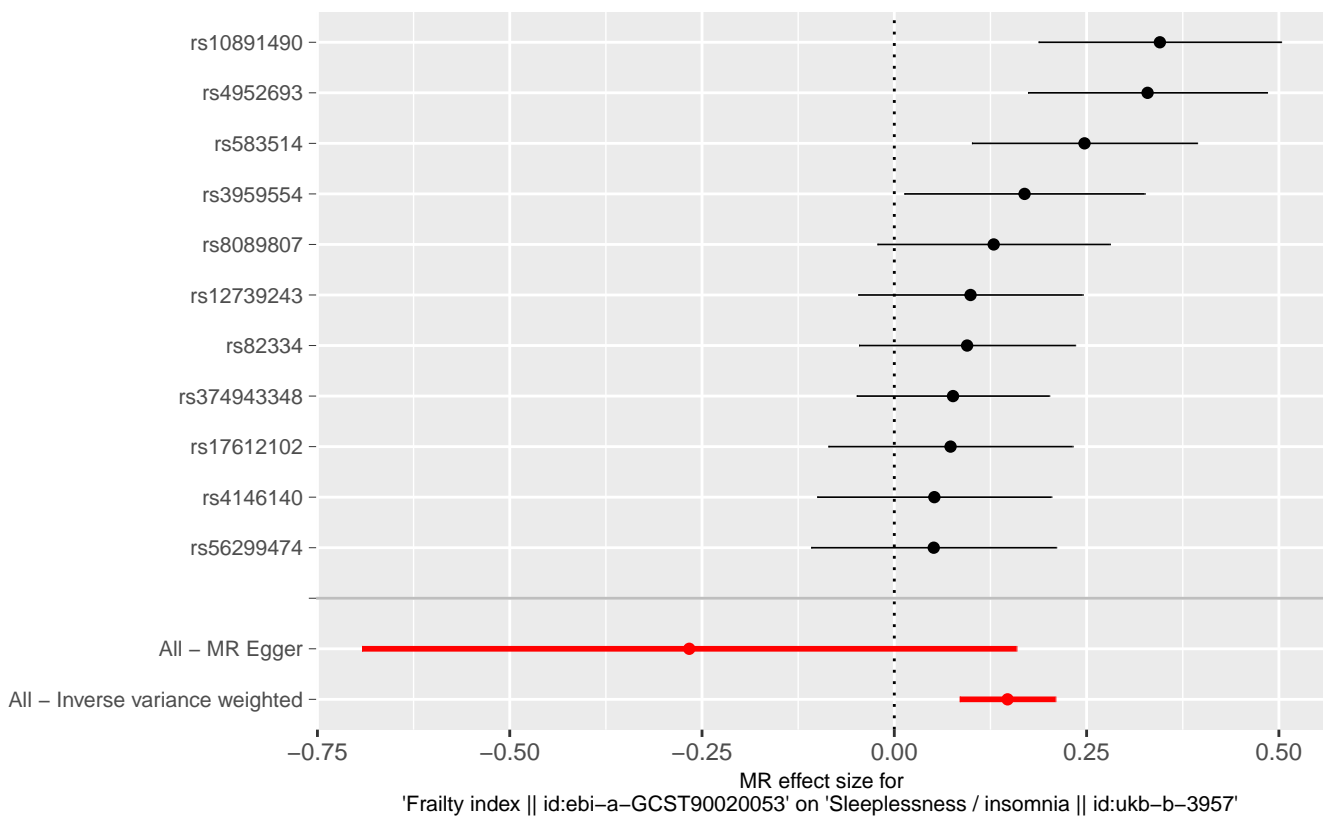

Supplement: Supplementary file 1 [file Data_Sheet_1.ZIP › Supplementary Materials/Insomnia/reverse/Insomnia reverse forest.pdf]

# MR Method

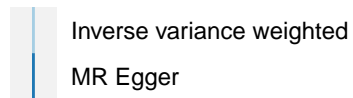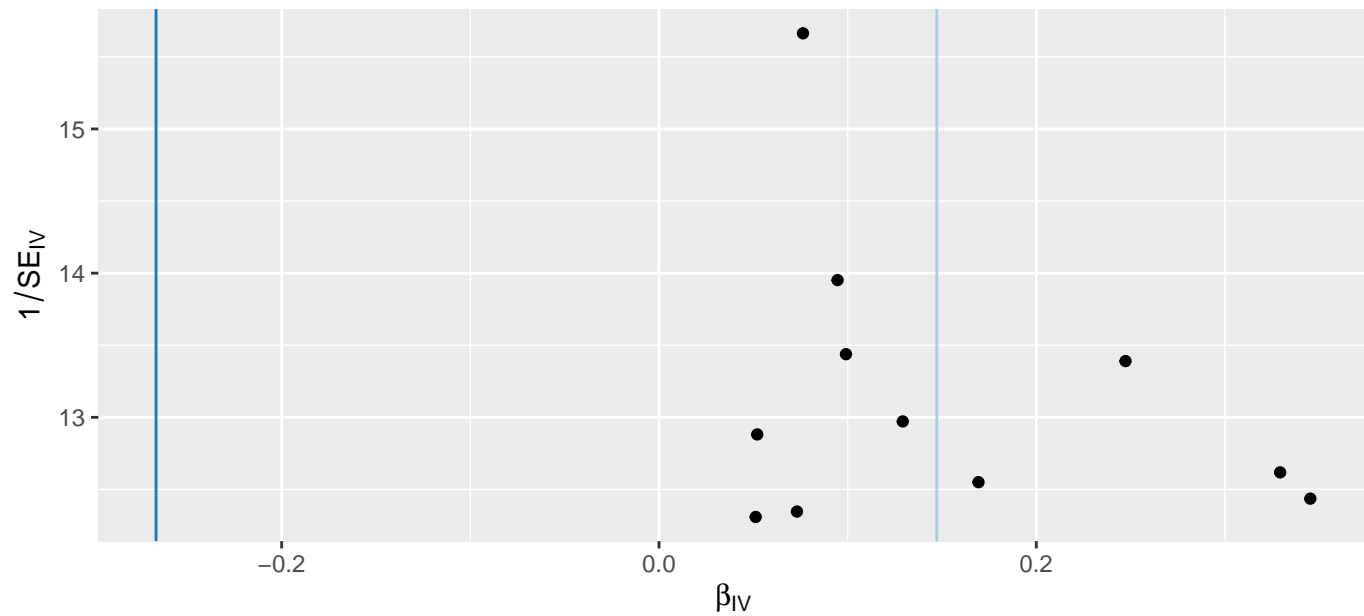

Supplement: Supplementary file 1 [file Data_Sheet_1.ZIP › Supplementary Materials/Insomnia/reverse/Insomnia reverse funnel.pdf]

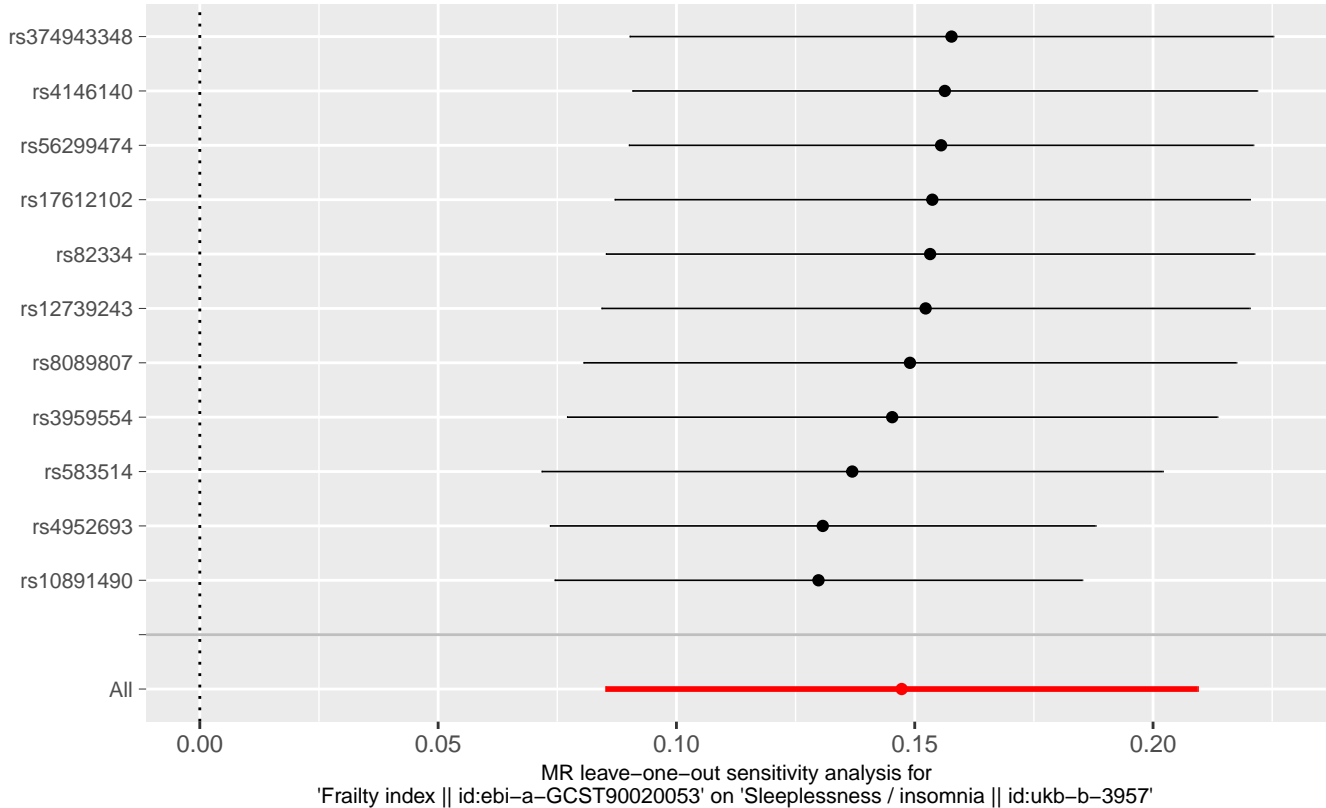

Supplement: Supplementary file 1 [file Data_Sheet_1.ZIP › Supplementary Materials/Insomnia/reverse/Insomnia reverse leave-one-out.pdf]

P effect on Sleeplessness / insomnia || id:ukb-b-3957

### MR Test

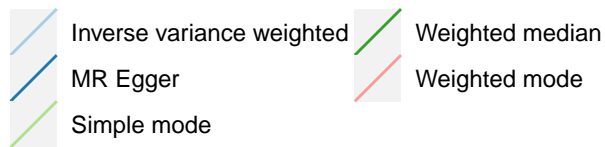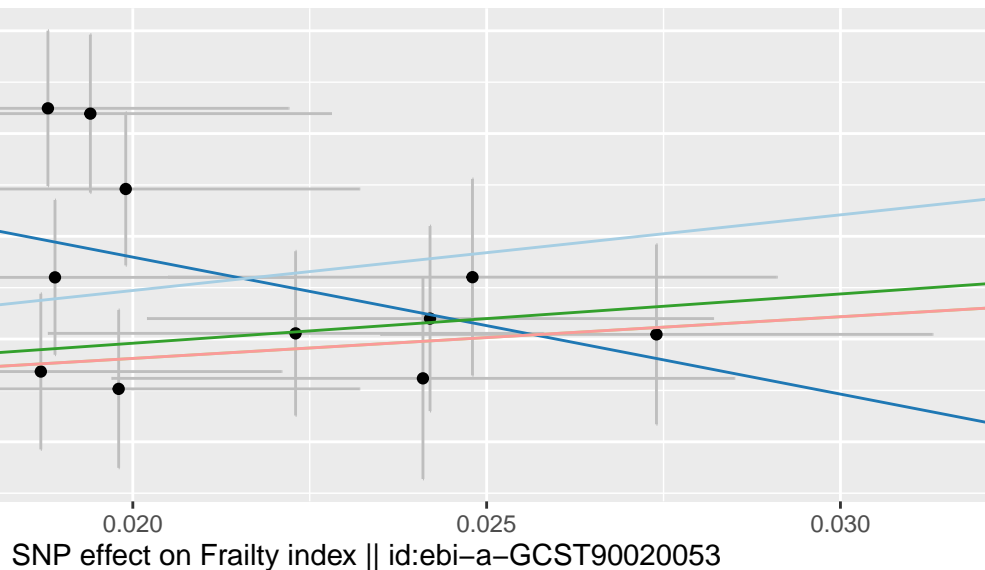

Supplement: Supplementary file 1 [file Data_Sheet_1.ZIP › Supplementary Materials/Insomnia/reverse/Insomnia reverse scatter.pdf]

All – Inverse variance weighted

MR Egger

MR Egger

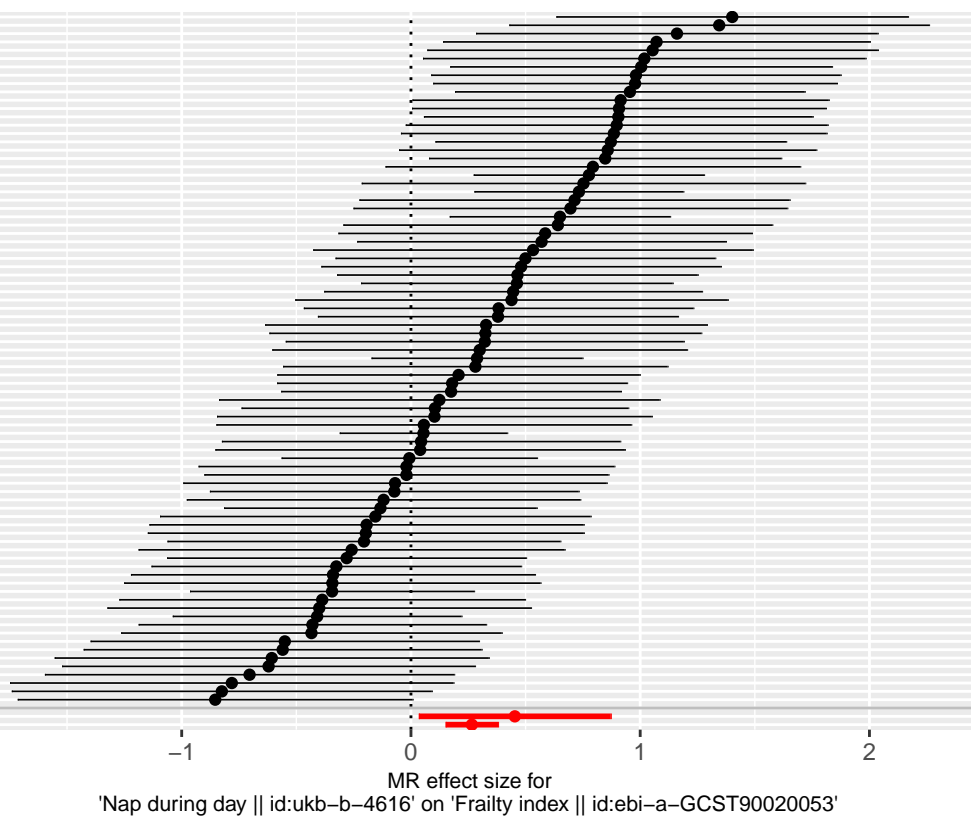

Supplement: Supplementary file 1 [file Data_Sheet_1.ZIP › Supplementary Materials/Nap during day/forward/Nap during day forward forest.pdf]

# MR Method

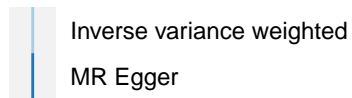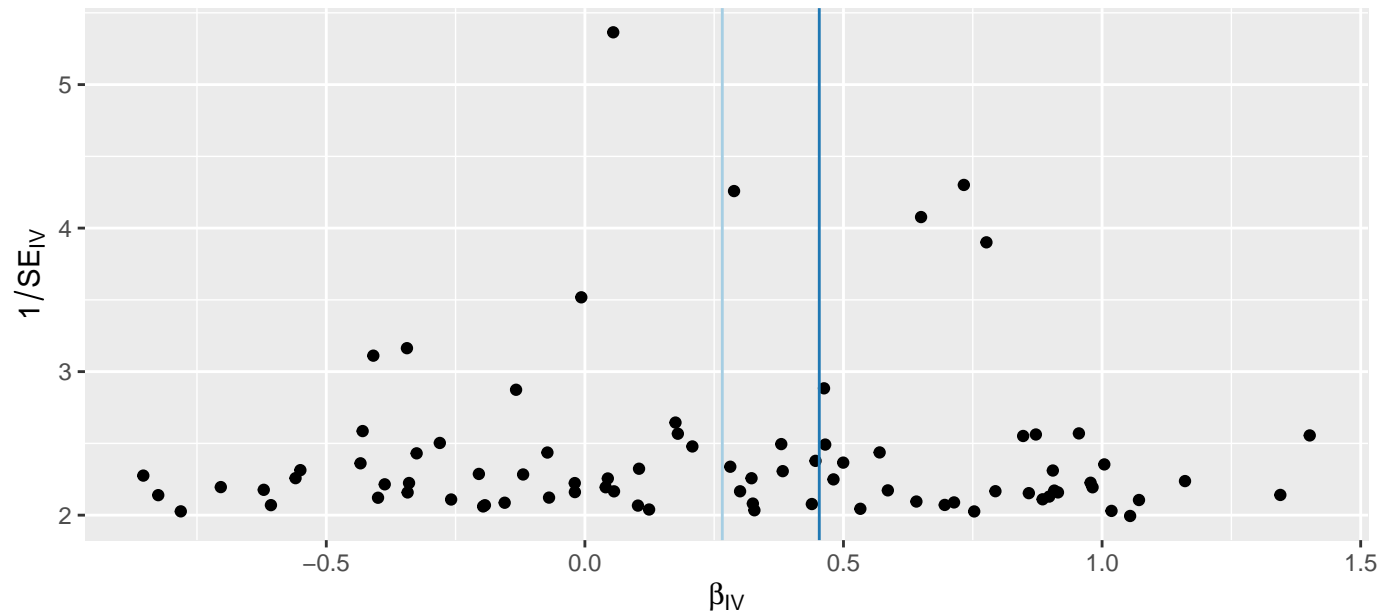

Supplement: Supplementary file 1 [file Data_Sheet_1.ZIP › Supplementary Materials/Nap during day/forward/Nap during day forward funnel.pdf]

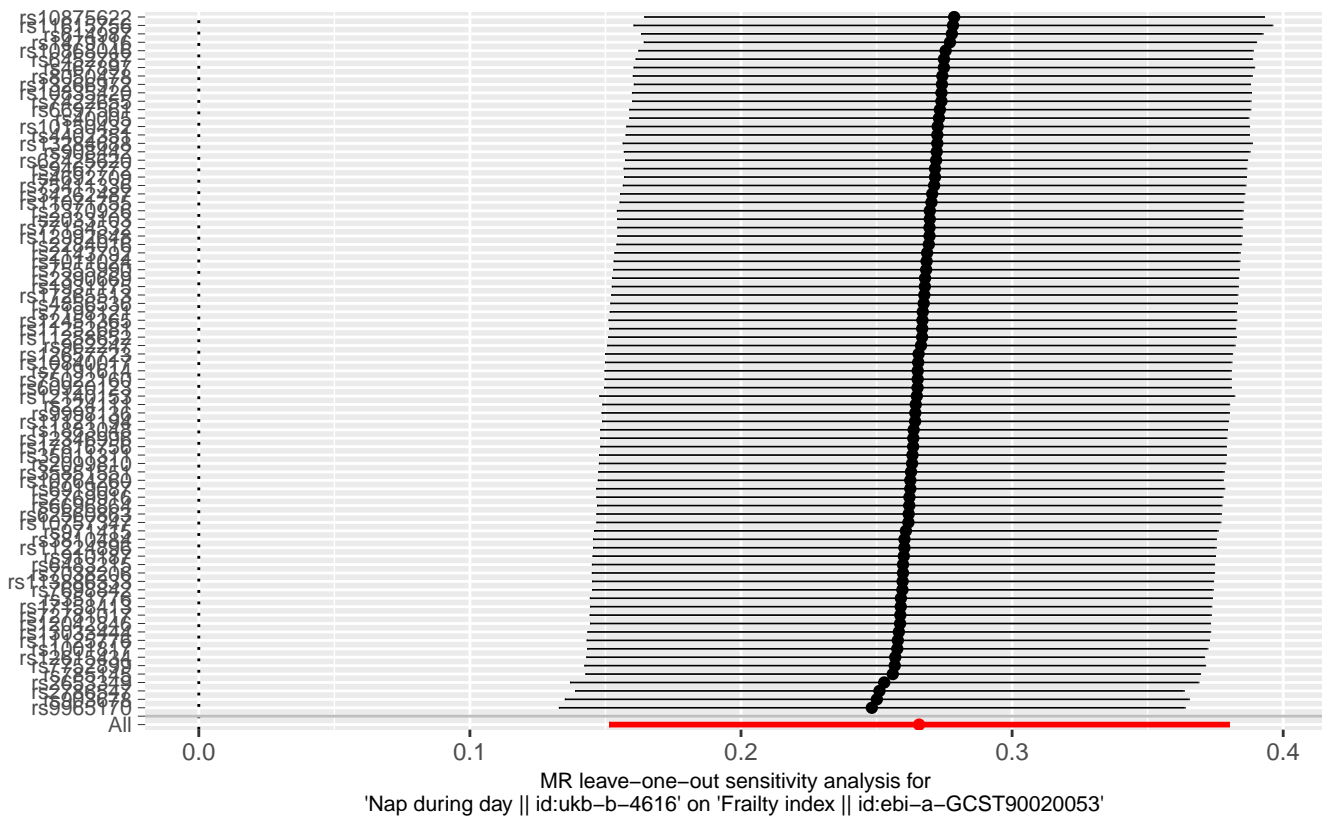

Supplement: Supplementary file 1 [file Data_Sheet_1.ZIP › Supplementary Materials/Nap during day/forward/Nap during day forward leave-one-out.pdf]

NIP effect on Frailty index || id:ebi-a-GCST90020053

### MR Test

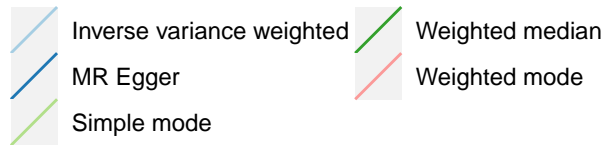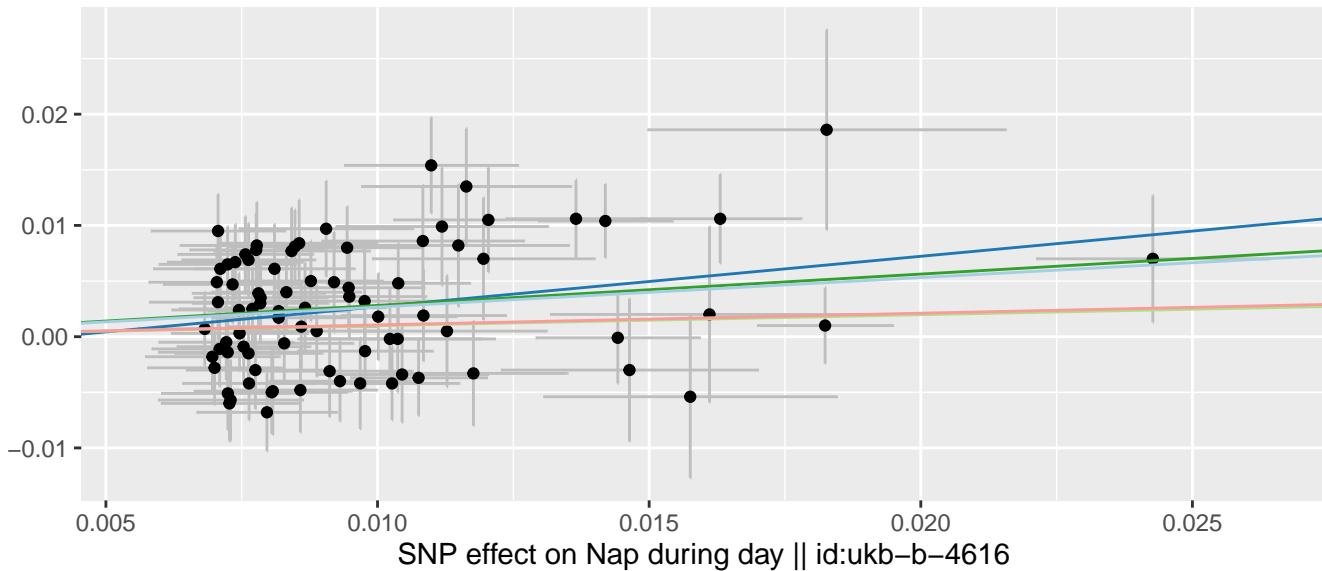

Supplement: Supplementary file 1 [file Data_Sheet_1.ZIP › Supplementary Materials/Nap during day/forward/Nap during day forward scatter.pdf]

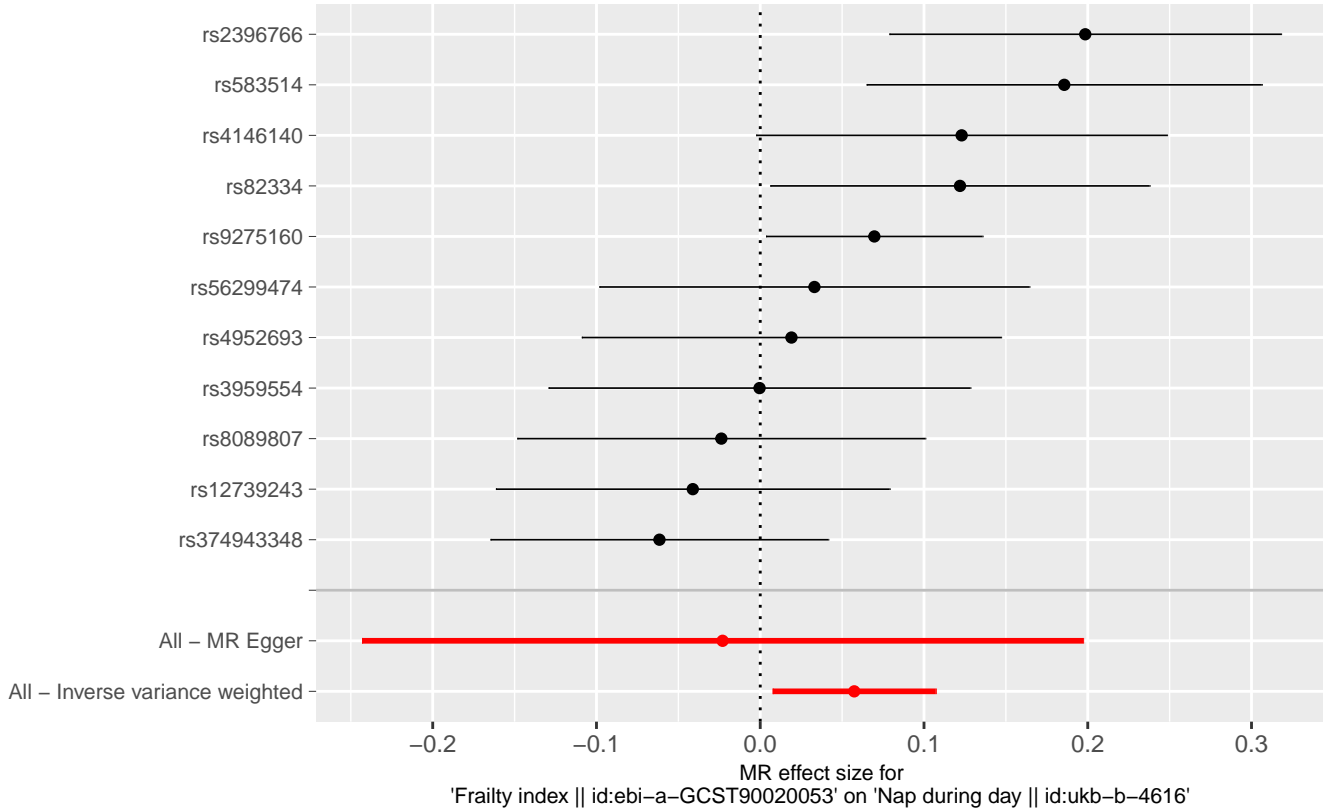

Supplement: Supplementary file 1 [file Data_Sheet_1.ZIP › Supplementary Materials/Nap during day/reverse/Nap during day reverse forest.pdf]

# MR Method

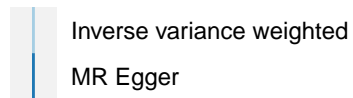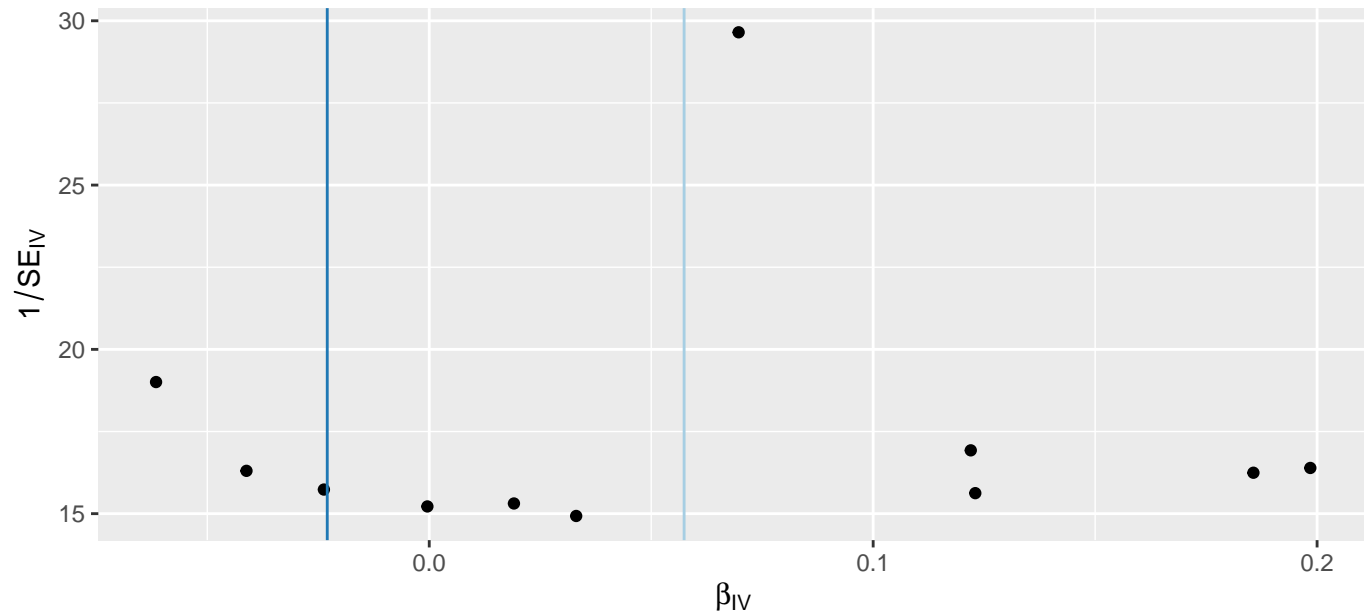

Supplement: Supplementary file 1 [file Data_Sheet_1.ZIP › Supplementary Materials/Nap during day/reverse/Nap during day reverse funnel.pdf]

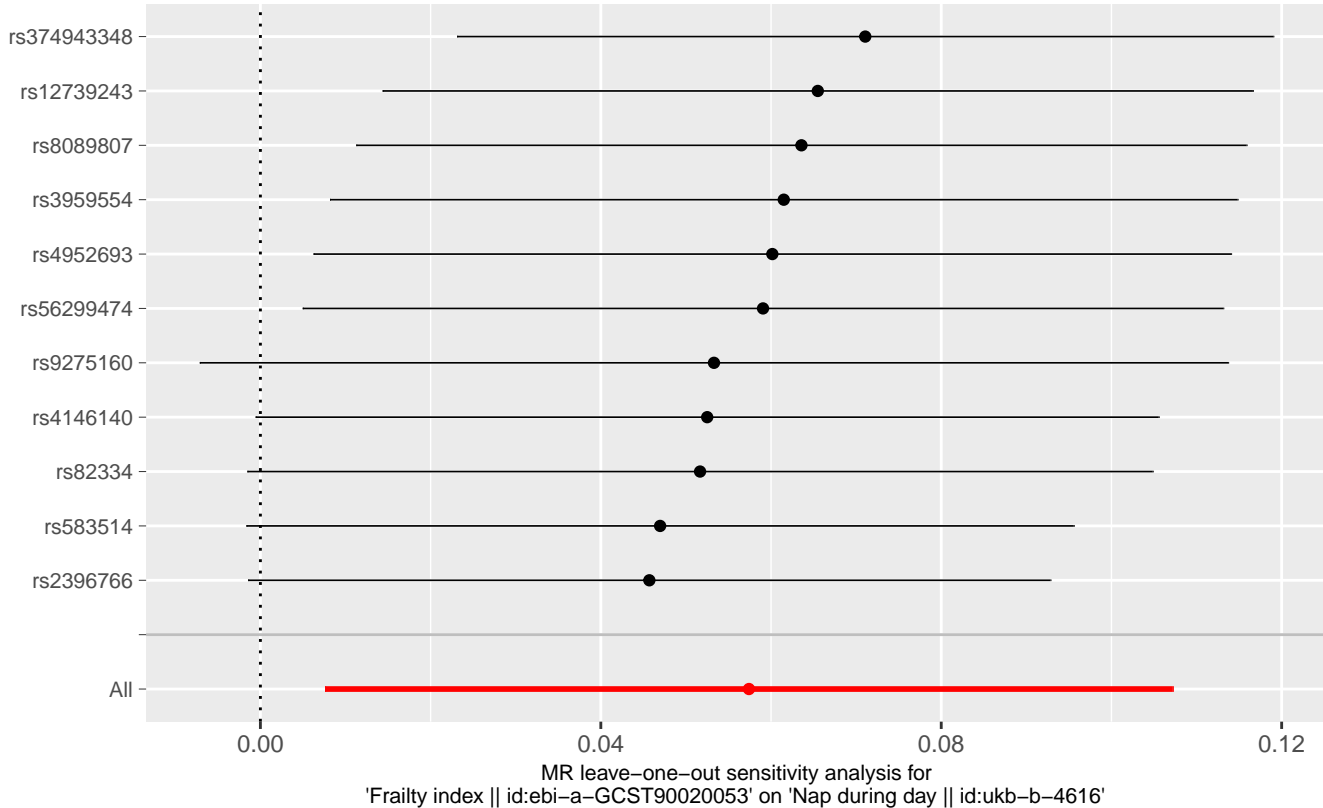

Supplement: Supplementary file 1 [file Data_Sheet_1.ZIP › Supplementary Materials/Nap during day/reverse/Nap during day reverse leave-one-out.pdf]

# MR Test

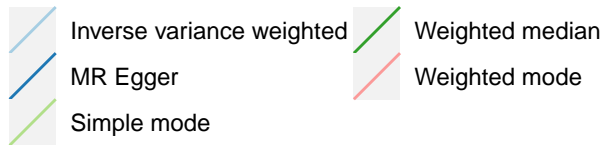

SNP effect on Nap during day || id:ukb-b-4616

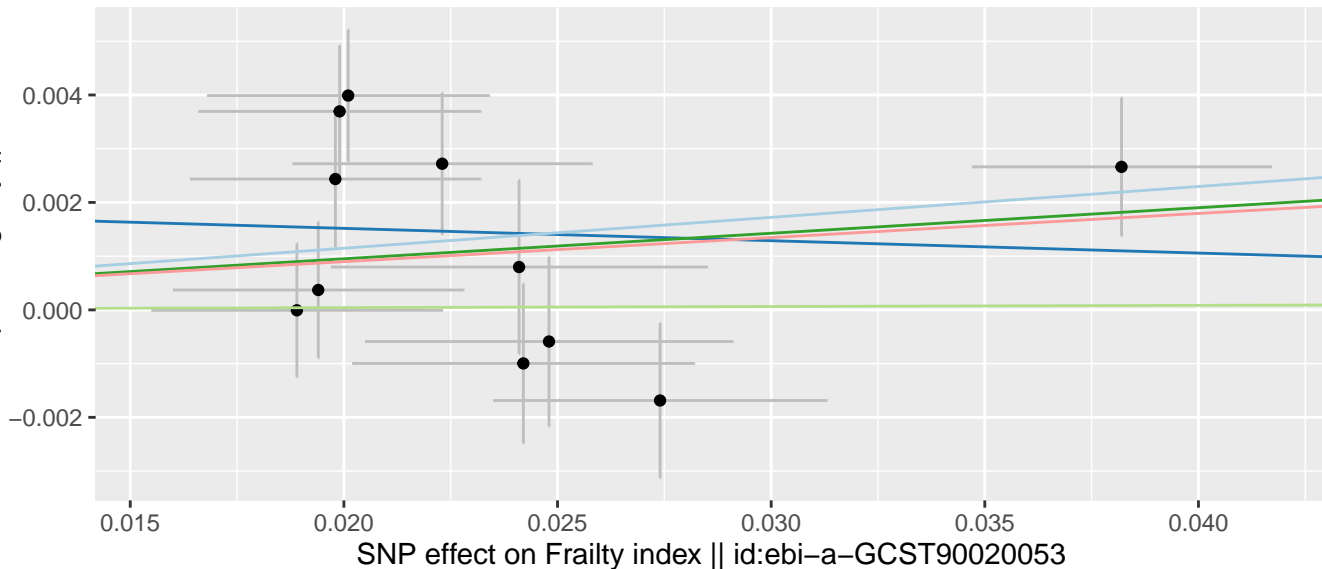

Supplement: Supplementary file 1 [file Data_Sheet_1.ZIP › Supplementary Materials/Nap during day/reverse/Nap during day reverse scatter.pdf]

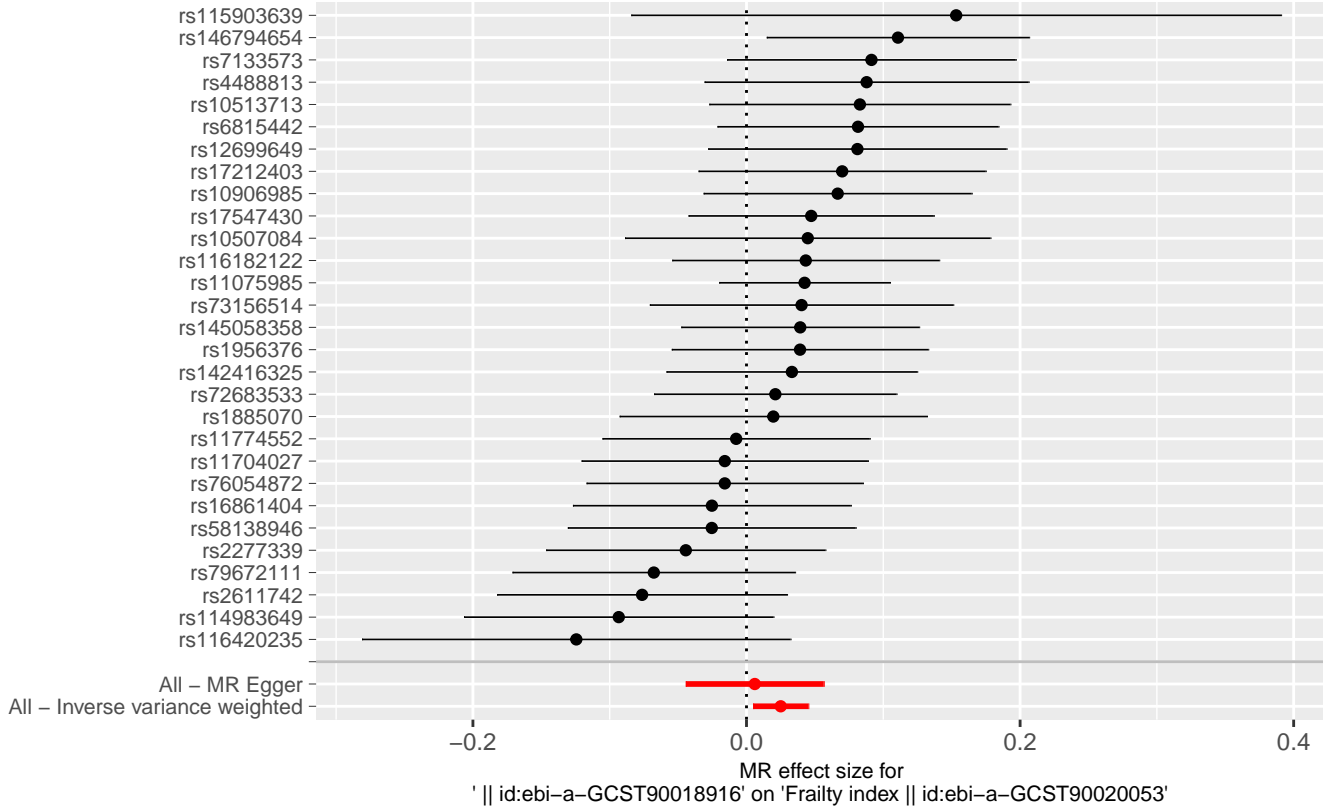

Supplement: Supplementary file 1 [file Data_Sheet_1.ZIP › Supplementary Materials/Sleep apnea syndrome/forward/Sleep apnea syndrome forward forest.pdf]

# MR Method

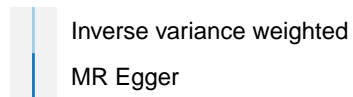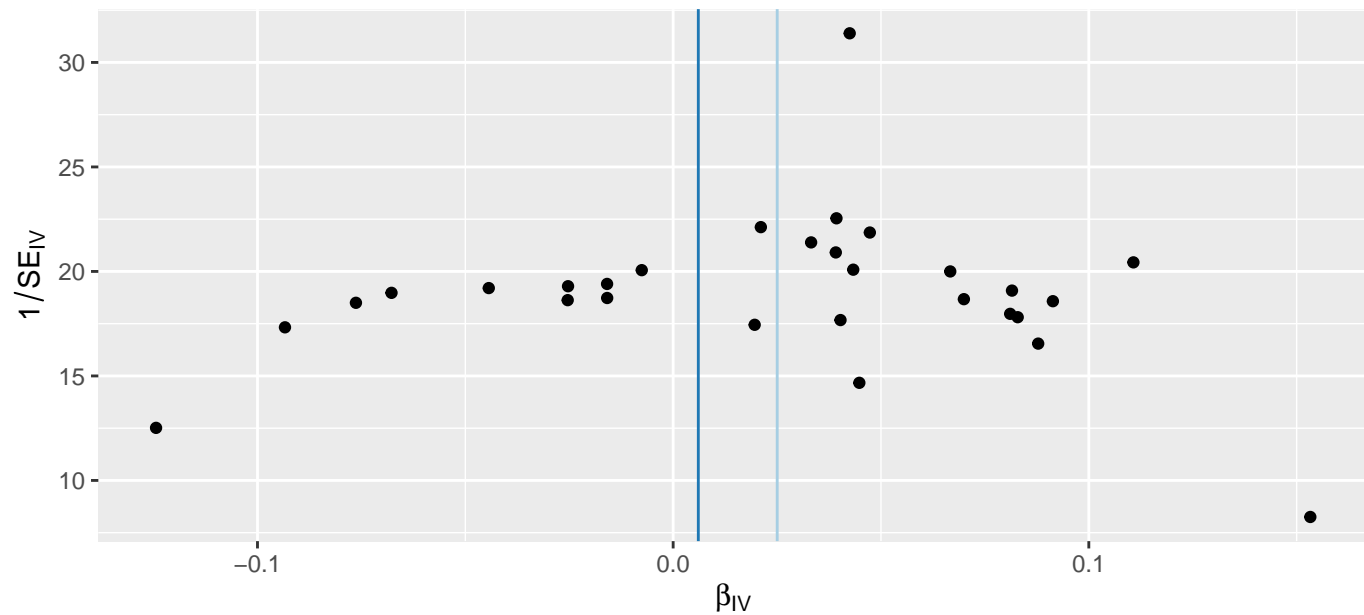

Supplement: Supplementary file 1 [file Data_Sheet_1.ZIP › Supplementary Materials/Sleep apnea syndrome/forward/Sleep apnea syndrome forward funnel.pdf]

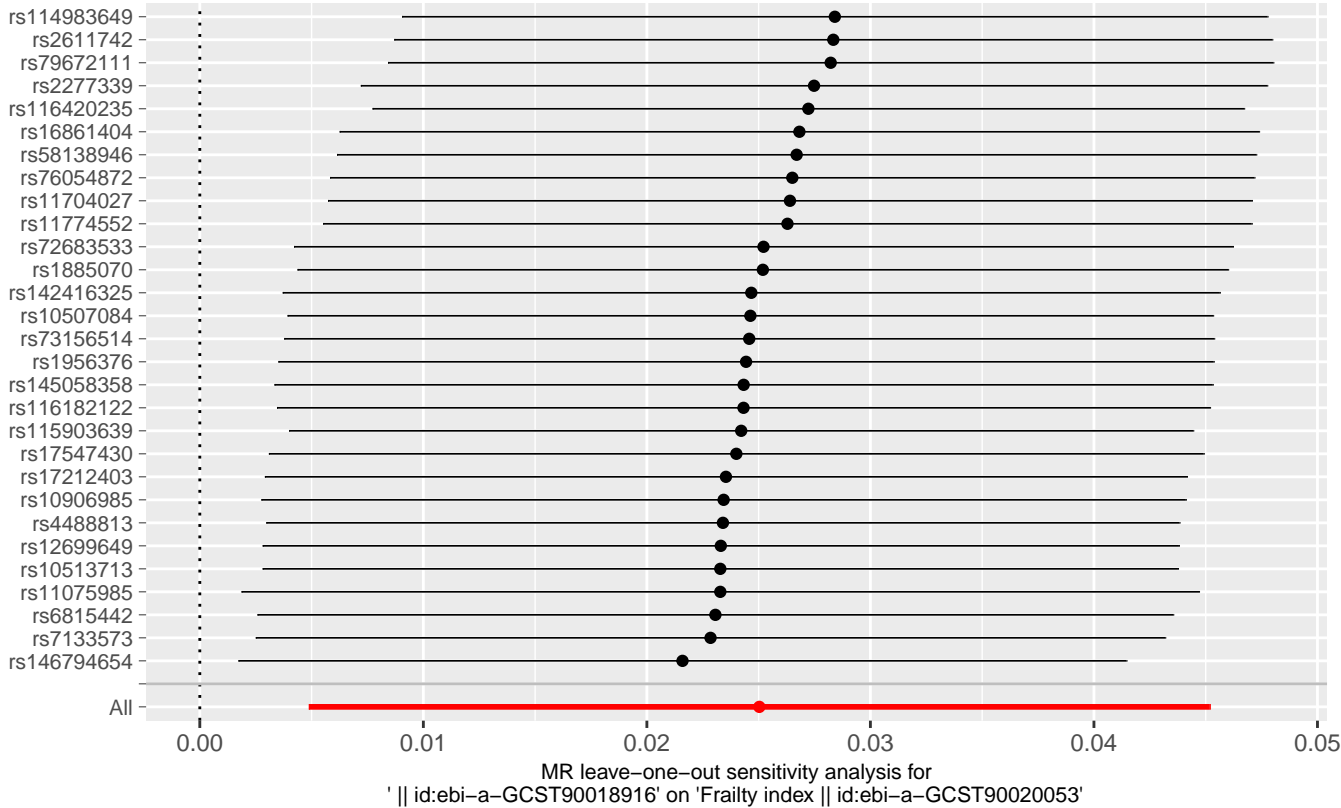

Supplement: Supplementary file 1 [file Data_Sheet_1.ZIP › Supplementary Materials/Sleep apnea syndrome/forward/Sleep apnea syndrome forward leave-one-out.pdf]

NIP effect on Frailty index || id:ebi-a-GCST90020053

### MR Test

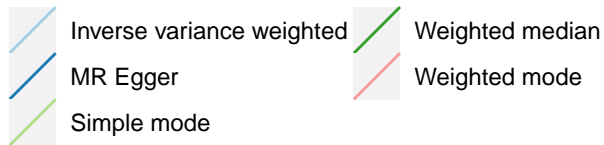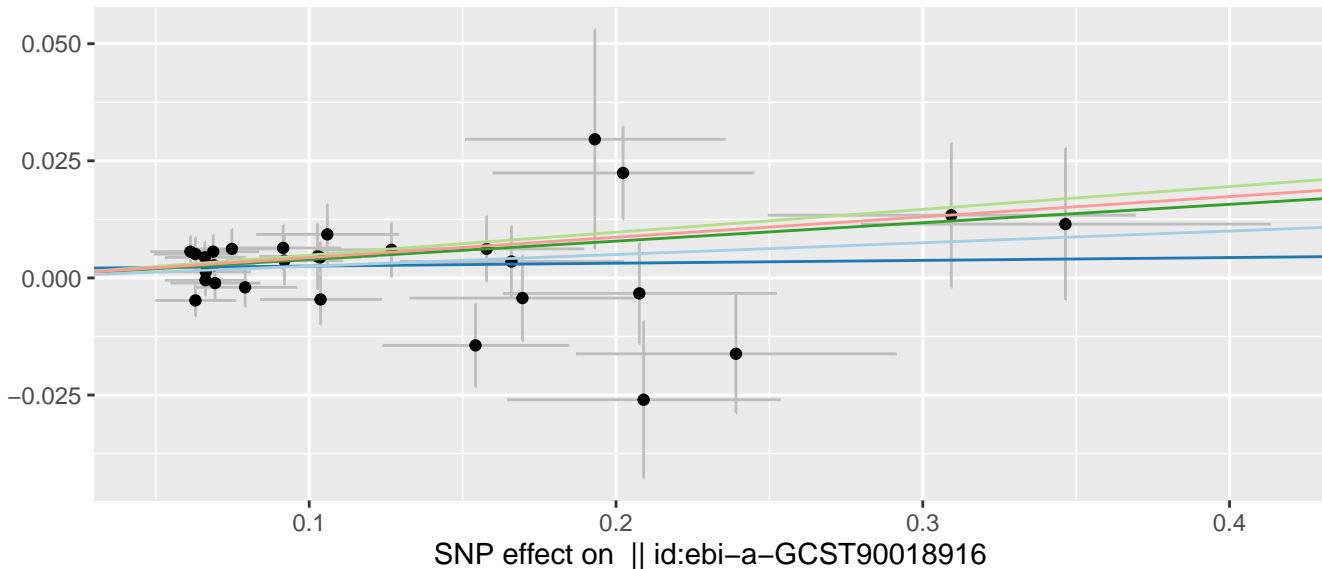

Supplement: Supplementary file 1 [file Data_Sheet_1.ZIP › Supplementary Materials/Sleep apnea syndrome/forward/Sleep apnea syndrome forward scatter.pdf]

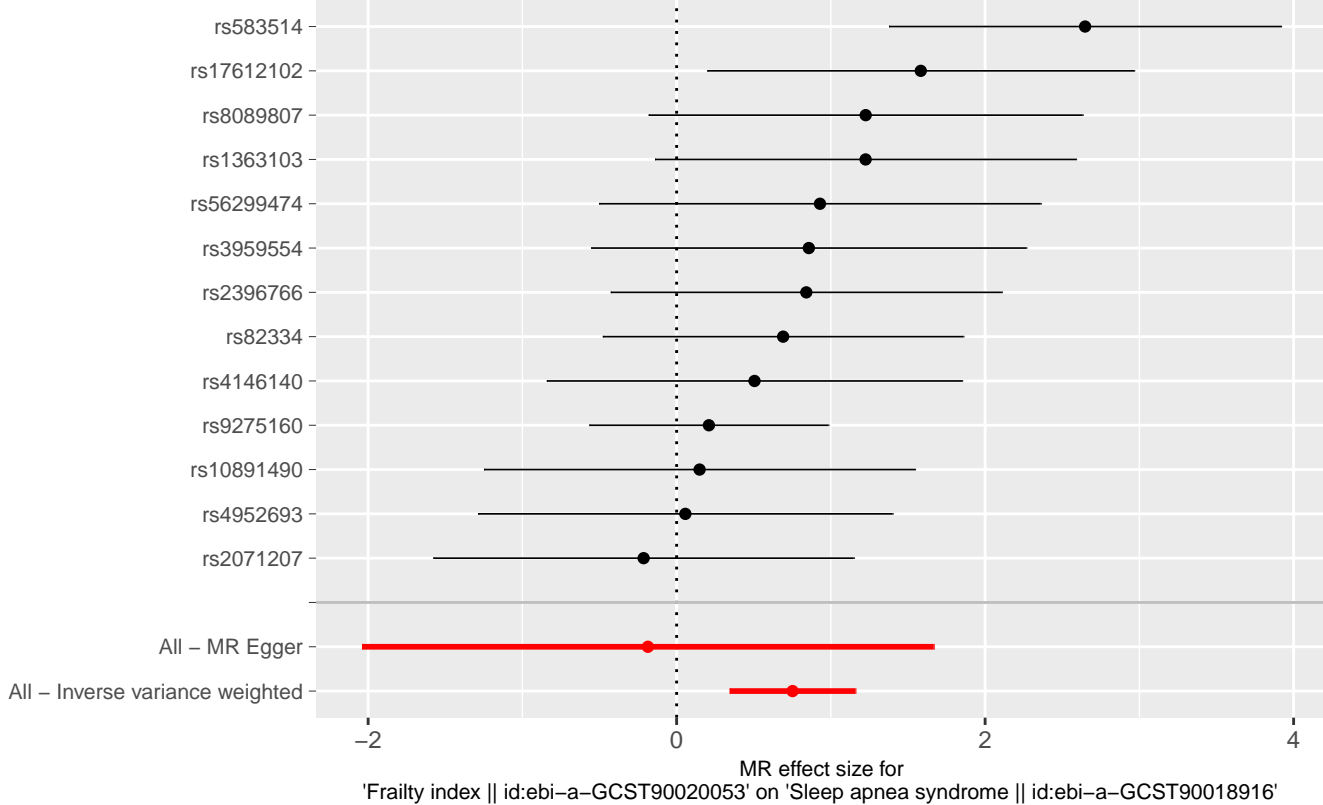

Supplement: Supplementary file 1 [file Data_Sheet_1.ZIP › Supplementary Materials/Sleep apnea syndrome/reverse/Sleep apnea syndrome reverse forest.pdf]

# MR Method

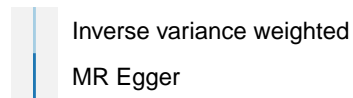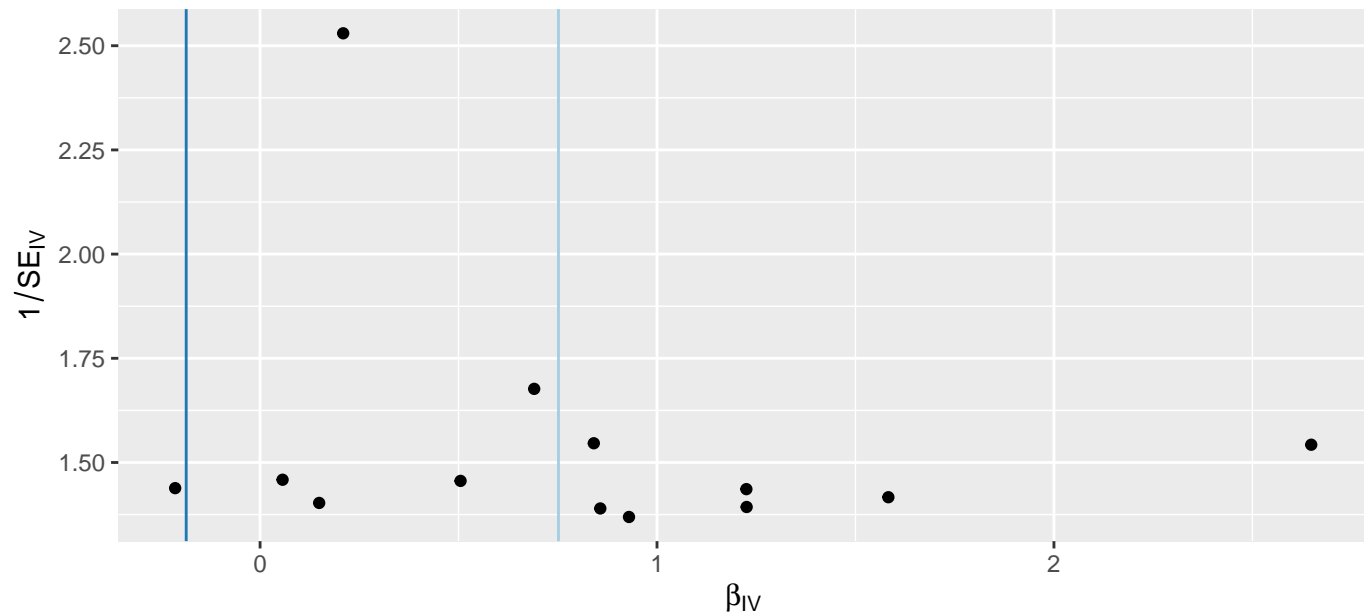

Supplement: Supplementary file 1 [file Data_Sheet_1.ZIP › Supplementary Materials/Sleep apnea syndrome/reverse/Sleep apnea syndrome reverse funnel.pdf]

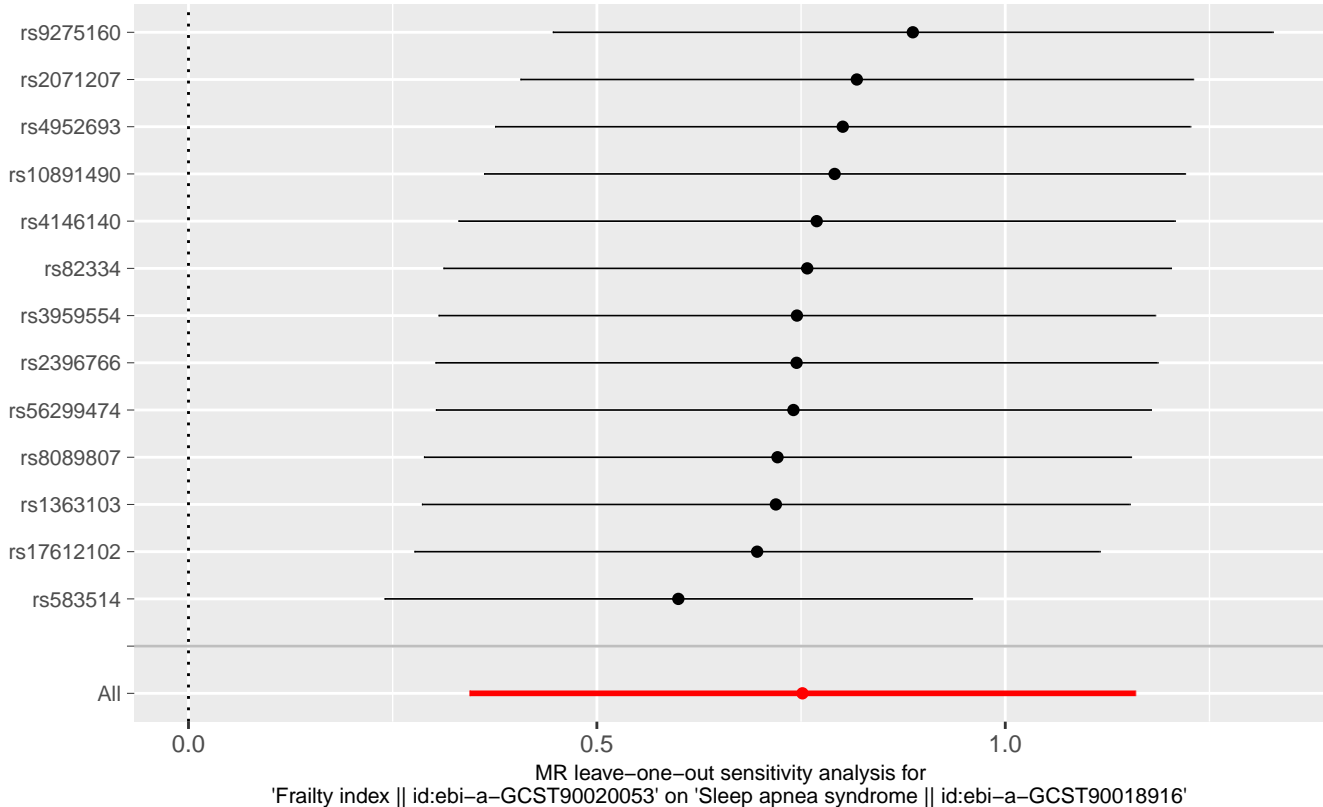

Supplement: Supplementary file 1 [file Data_Sheet_1.ZIP › Supplementary Materials/Sleep apnea syndrome/reverse/Sleep apnea syndrome reverse leave-one-out.pdf]

effect on Sleep apnea syndrome || id:ebi-a-GCST90018916

### MR Test

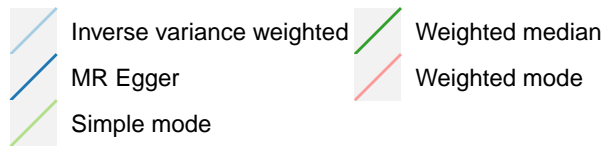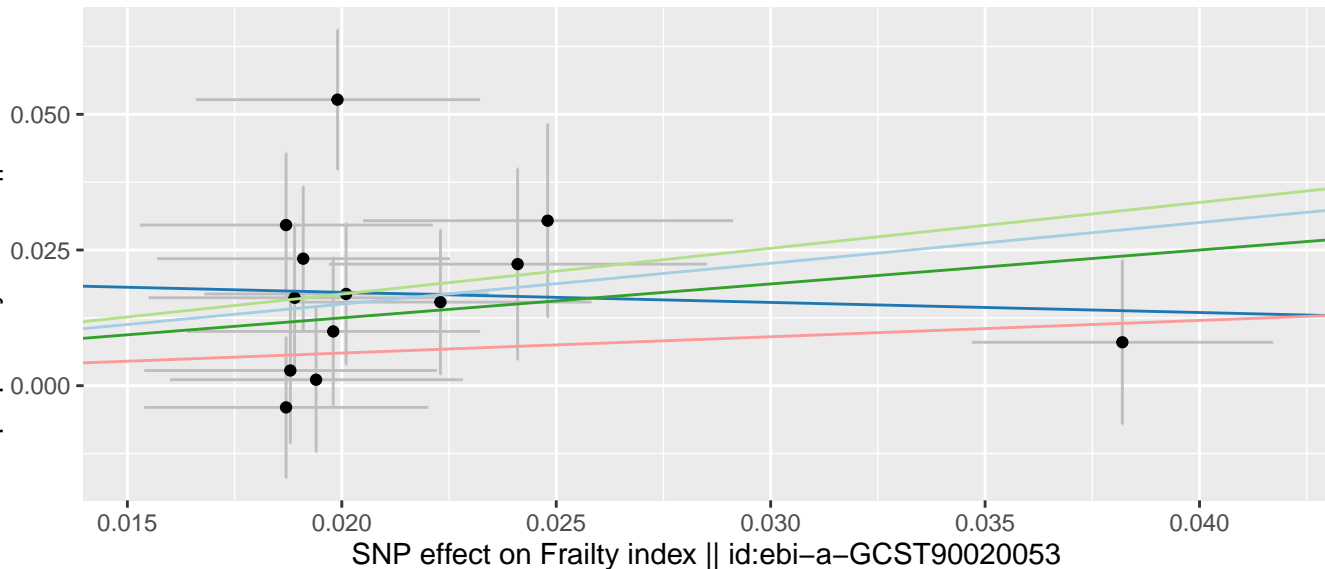

Supplement: Supplementary file 1 [file Data_Sheet_1.ZIP › Supplementary Materials/Sleep apnea syndrome/reverse/Sleep apnea syndrome reverse scatter.pdf]

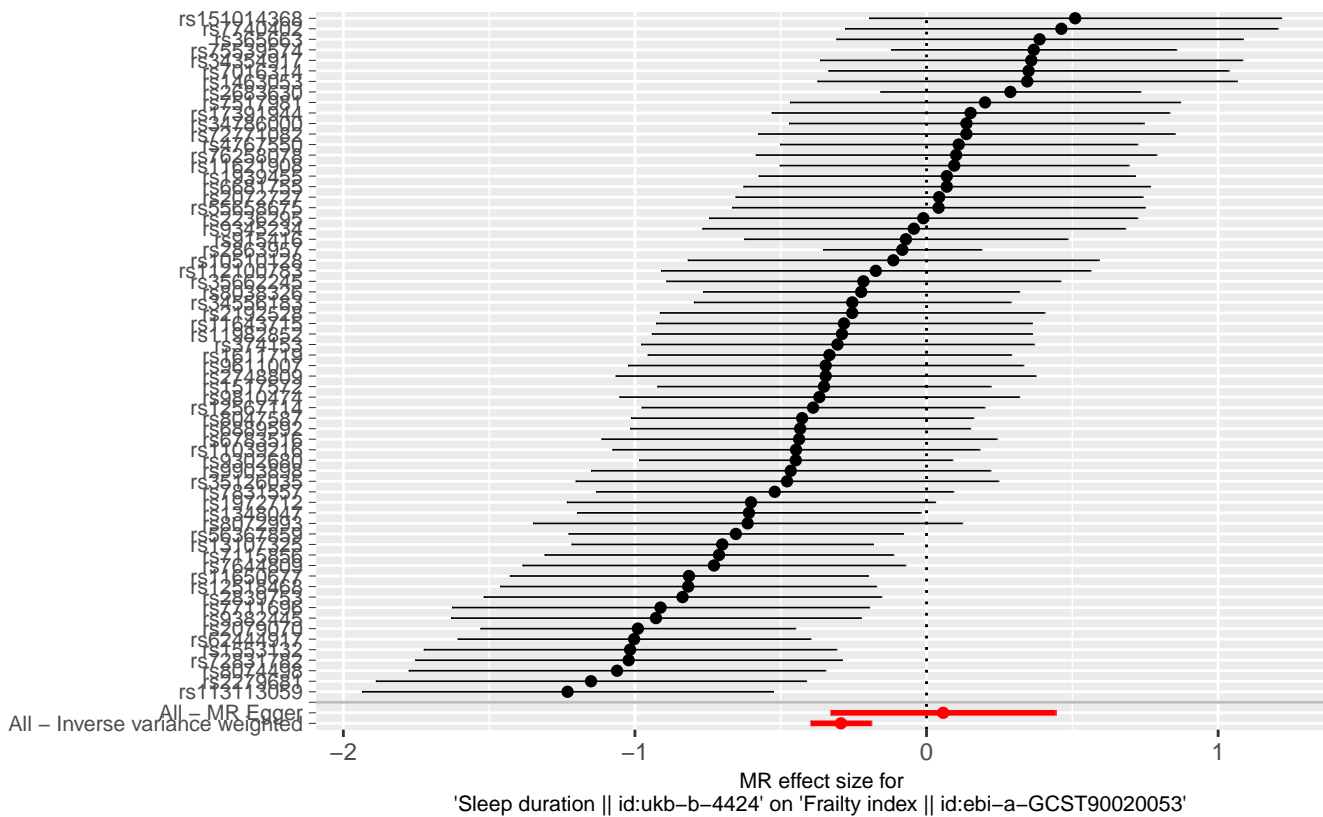

Supplement: Supplementary file 1 [file Data_Sheet_1.ZIP › Supplementary Materials/Sleep duration/forward/Sleep duration forward forest.pdf]

## MR Method

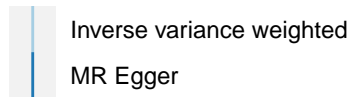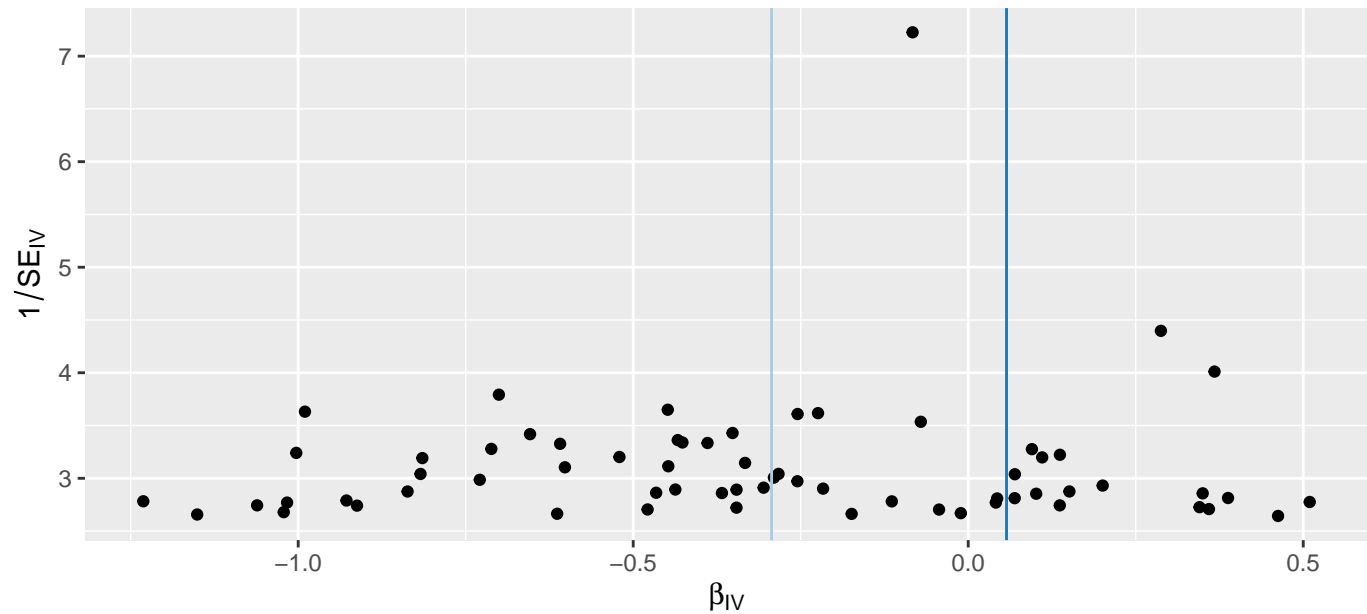

Supplement: Supplementary file 1 [file Data_Sheet_1.ZIP › Supplementary Materials/Sleep duration/forward/Sleep duration forward funnel.pdf]

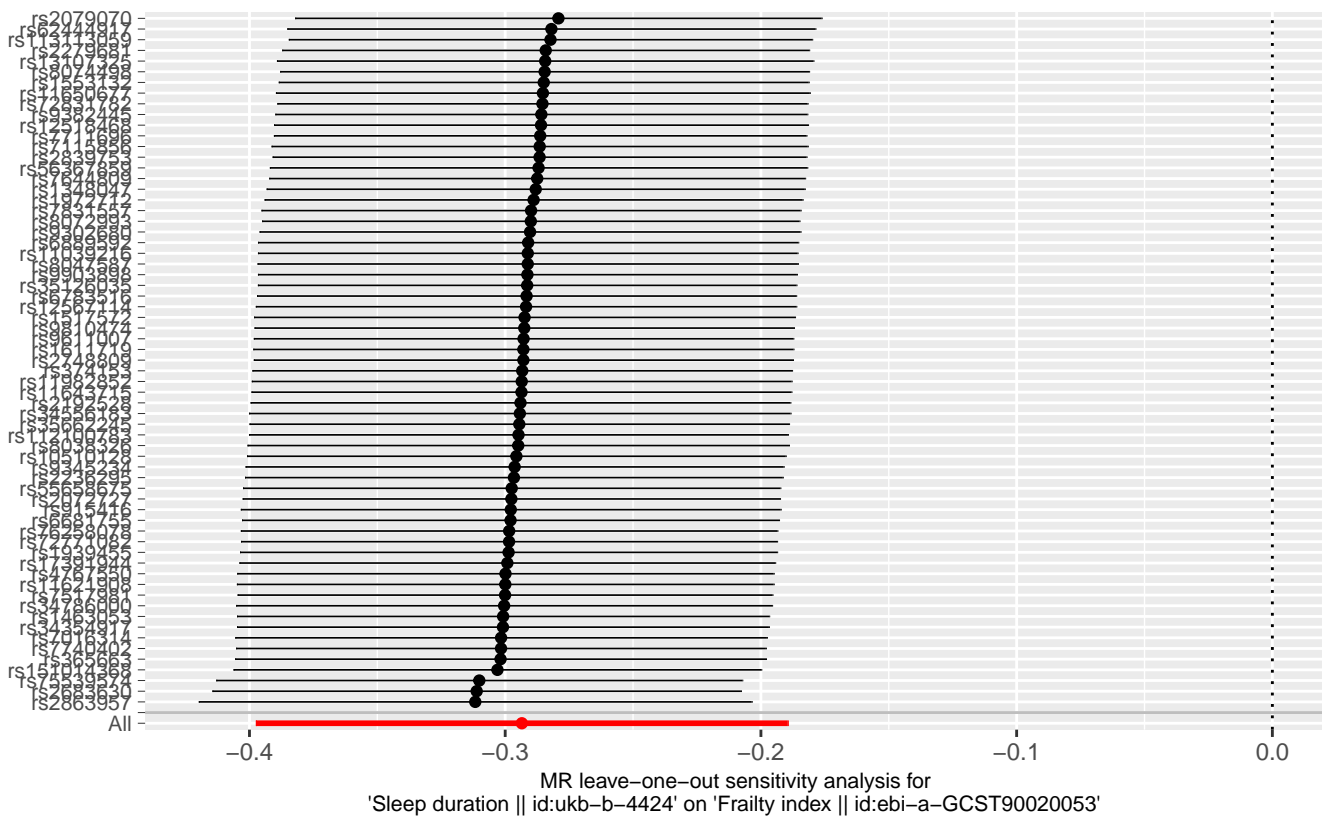

Supplement: Supplementary file 1 [file Data_Sheet_1.ZIP › Supplementary Materials/Sleep duration/forward/Sleep duration forward leave-one-out.pdf]

## MR Test

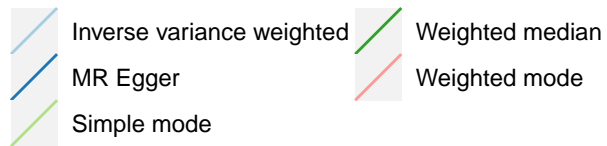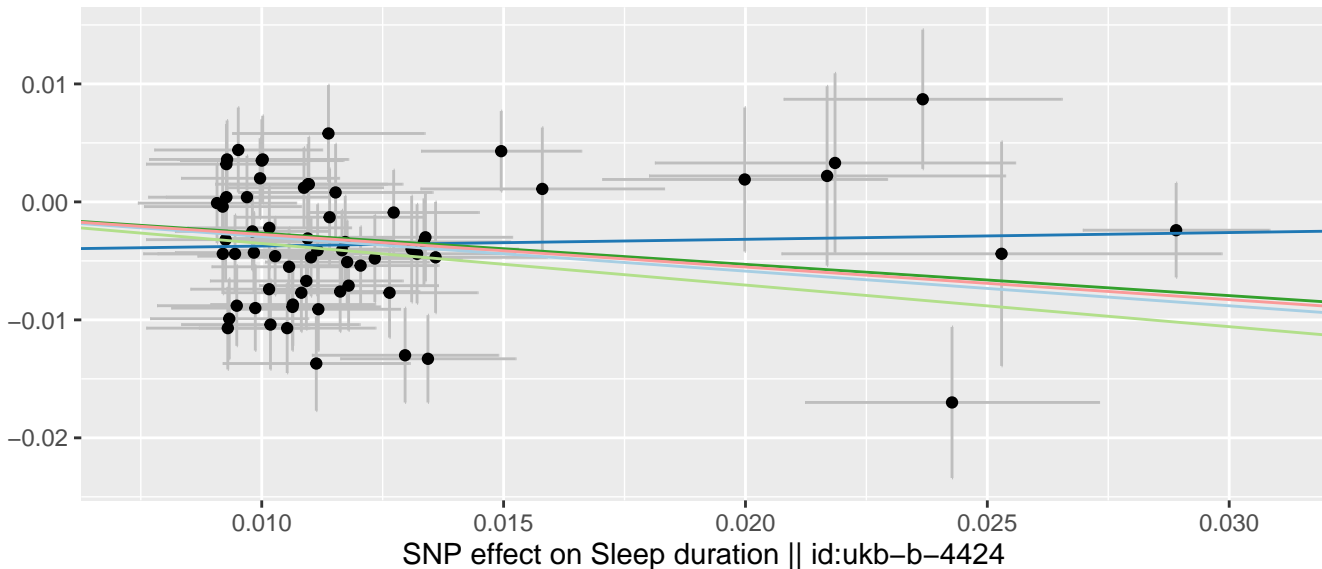

Supplement: Supplementary file 1 [file Data_Sheet_1.ZIP › Supplementary Materials/Sleep duration/forward/Sleep duration forward scatter.pdf]

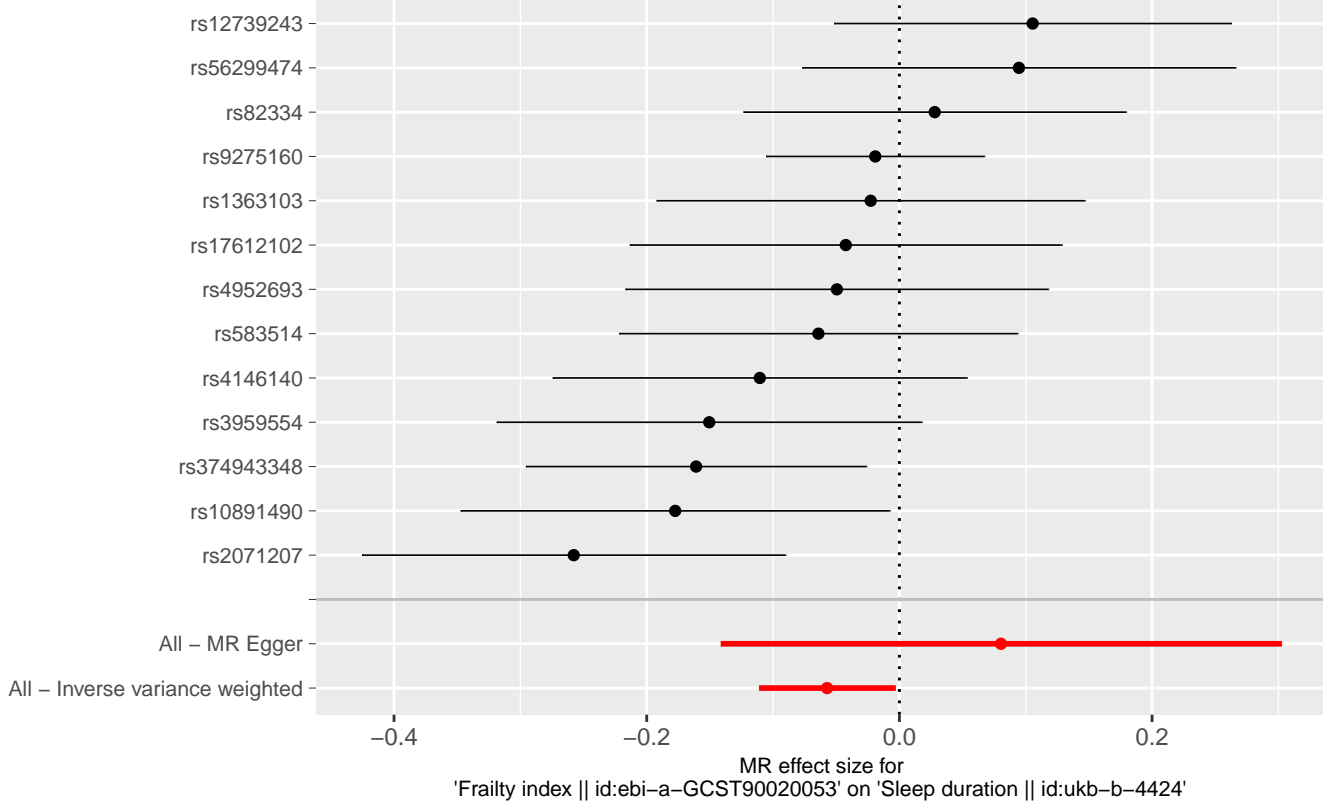

Supplement: Supplementary file 1 [file Data_Sheet_1.ZIP › Supplementary Materials/Sleep duration/reverse/Sleep duration reverse forest.pdf]

# MR Method

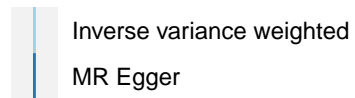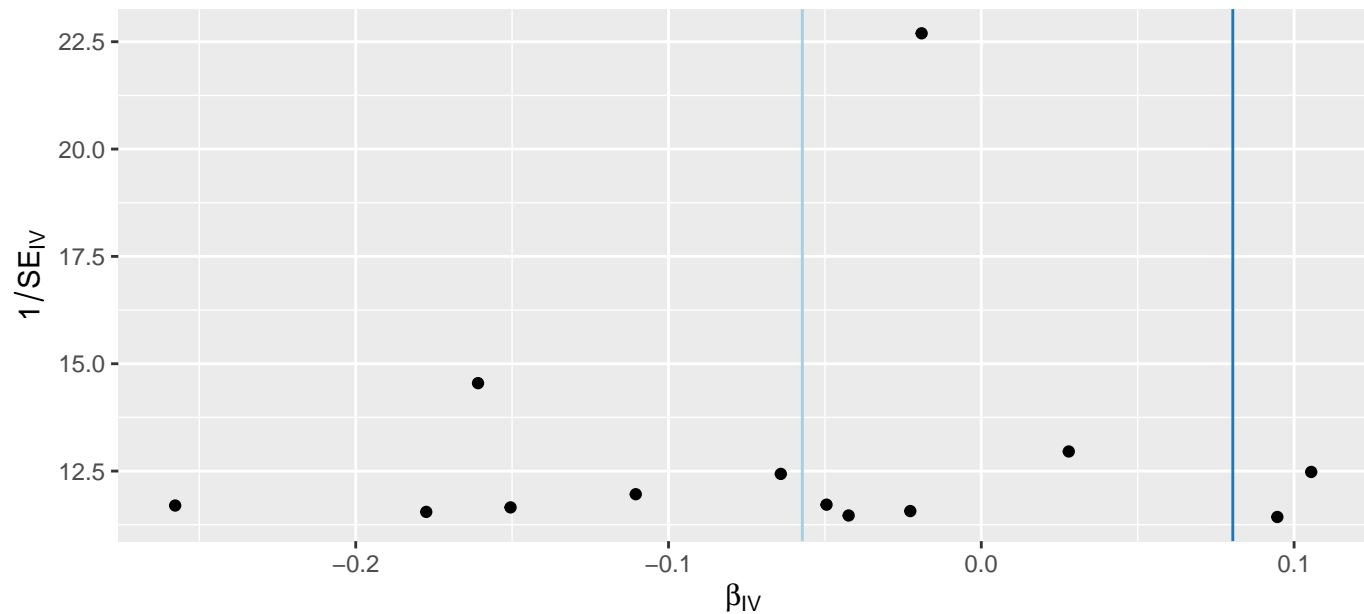

Supplement: Supplementary file 1 [file Data_Sheet_1.ZIP › Supplementary Materials/Sleep duration/reverse/Sleep duration reverse funnel.pdf]

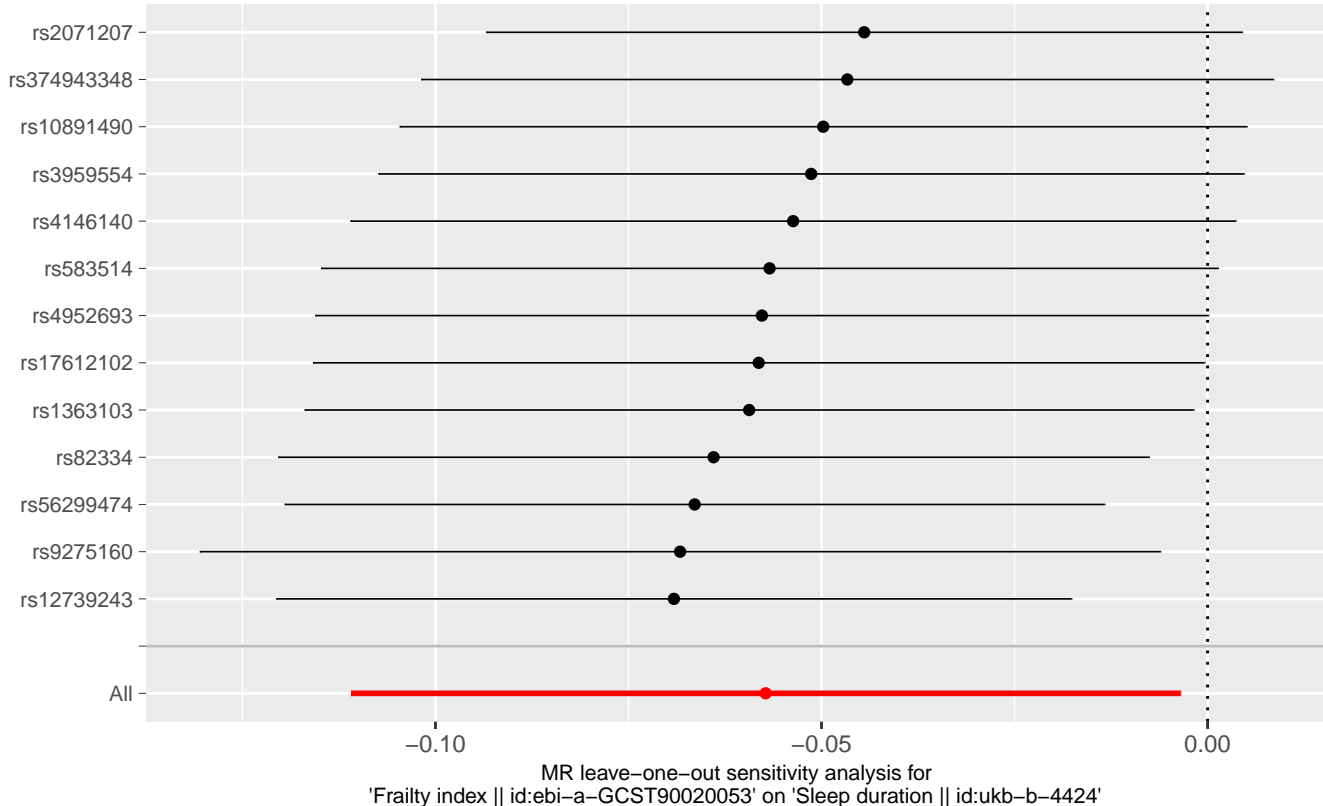

Supplement: Supplementary file 1 [file Data_Sheet_1.ZIP › Supplementary Materials/Sleep duration/reverse/Sleep duration reverse leave-one-out.pdf]

SNP effect on Sleep duration || id:ukb-b-4424

# MR Test

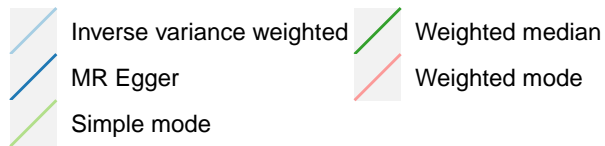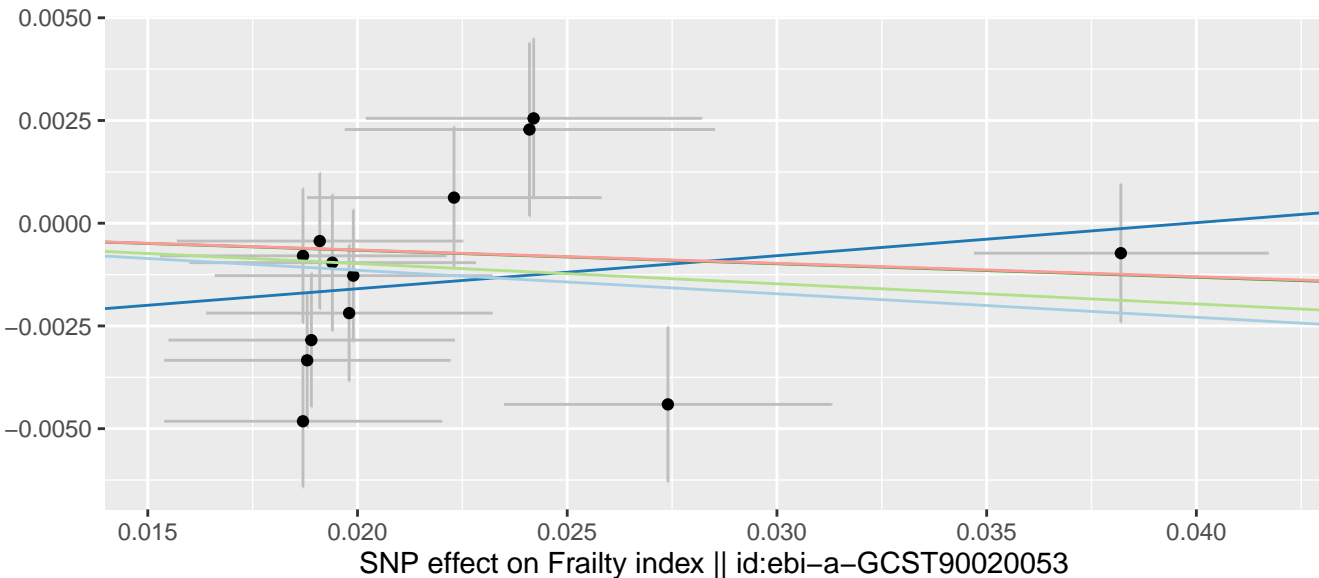

Supplement: Supplementary file 1 [file Data_Sheet_1.ZIP › Supplementary Materials/Sleep duration/reverse/Sleep duration reverse scatter.pdf]

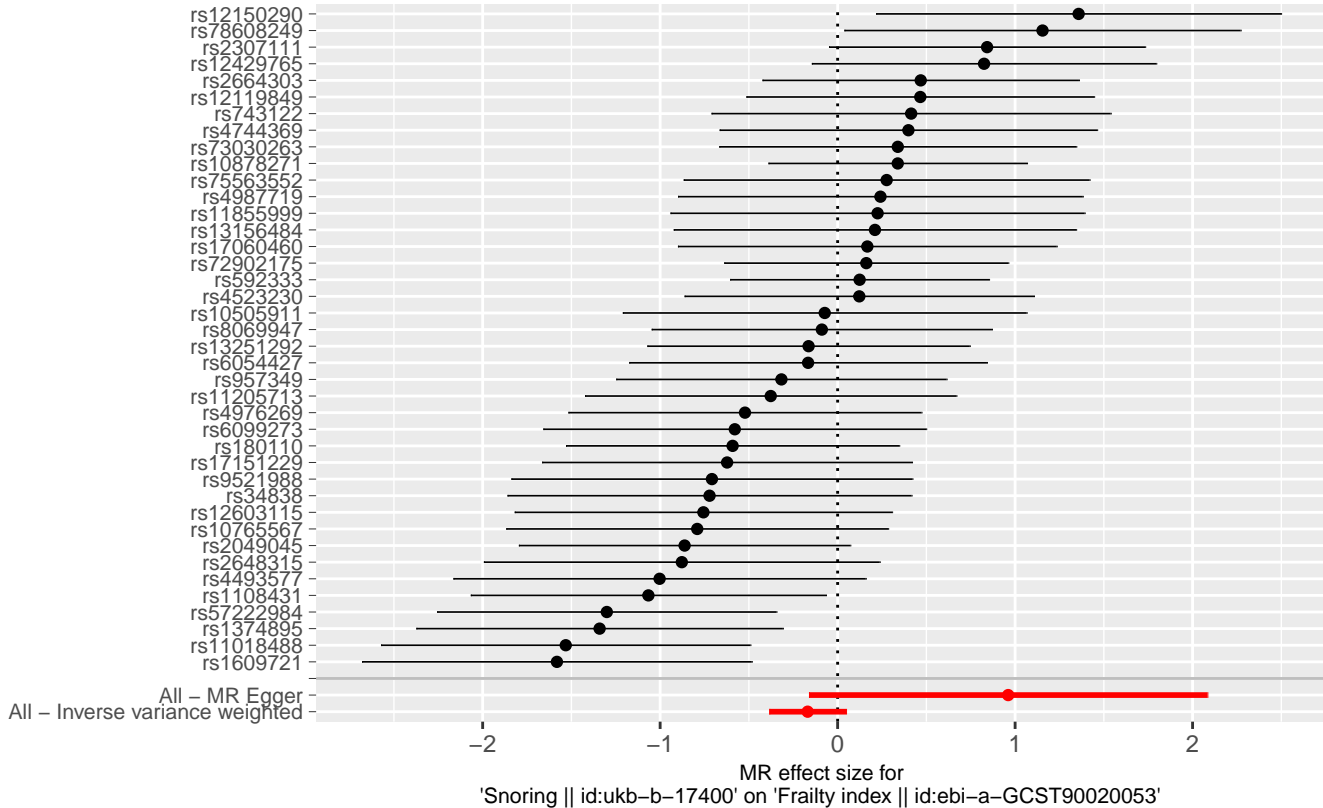

Supplement: Supplementary file 1 [file Data_Sheet_1.ZIP › Supplementary Materials/Snoring/forward/Snoring forward forest.pdf]

# MR Method

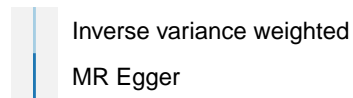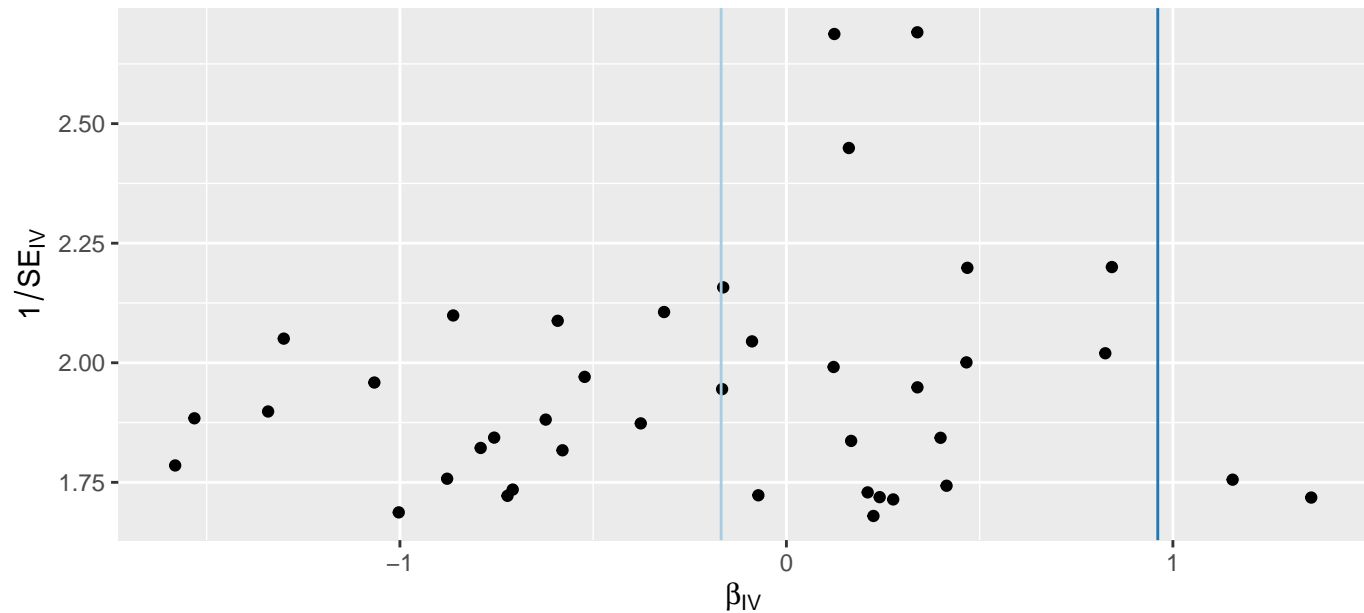

Supplement: Supplementary file 1 [file Data_Sheet_1.ZIP › Supplementary Materials/Snoring/forward/Snoring forward funnel.pdf]

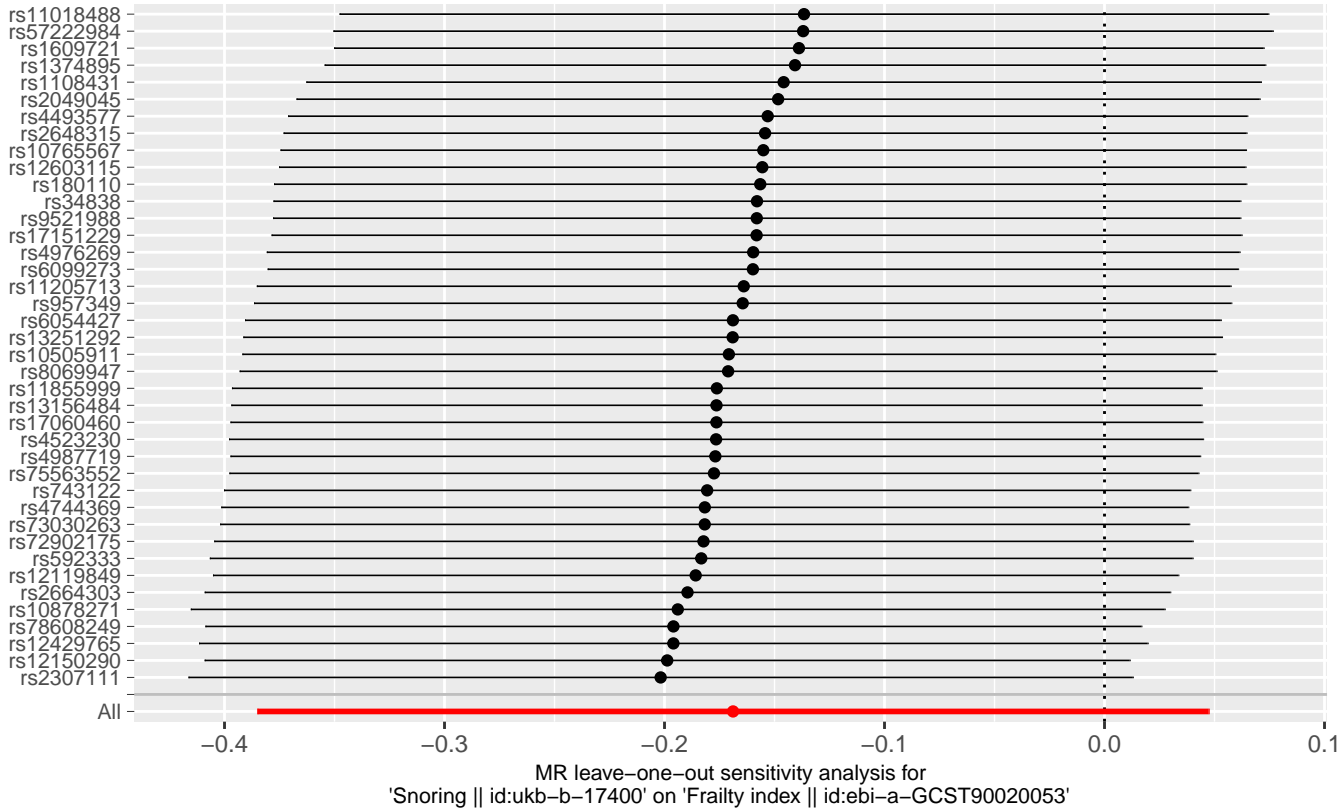

Supplement: Supplementary file 1 [file Data_Sheet_1.ZIP › Supplementary Materials/Snoring/forward/Snoring forward leave-one-out.pdf]

# MR Test

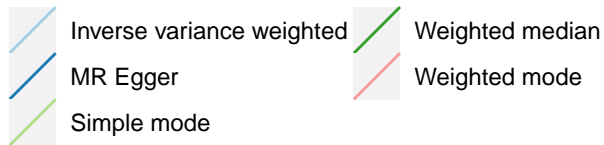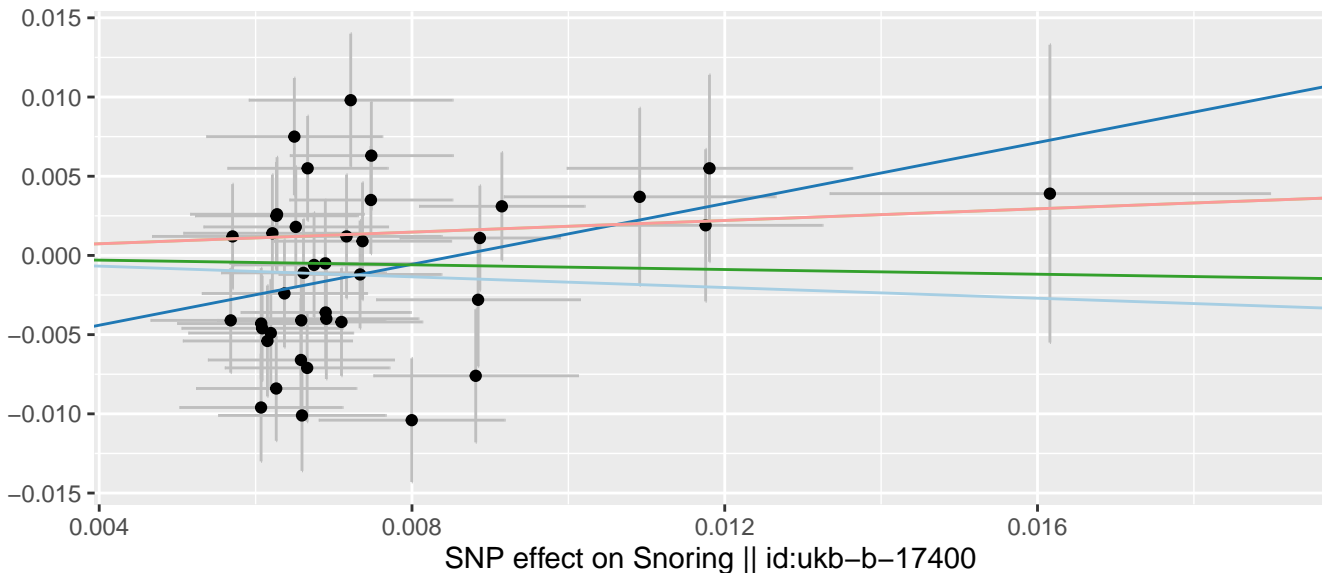

Supplement: Supplementary file 1 [file Data_Sheet_1.ZIP › Supplementary Materials/Snoring/forward/Snoring forward scatter.pdf]

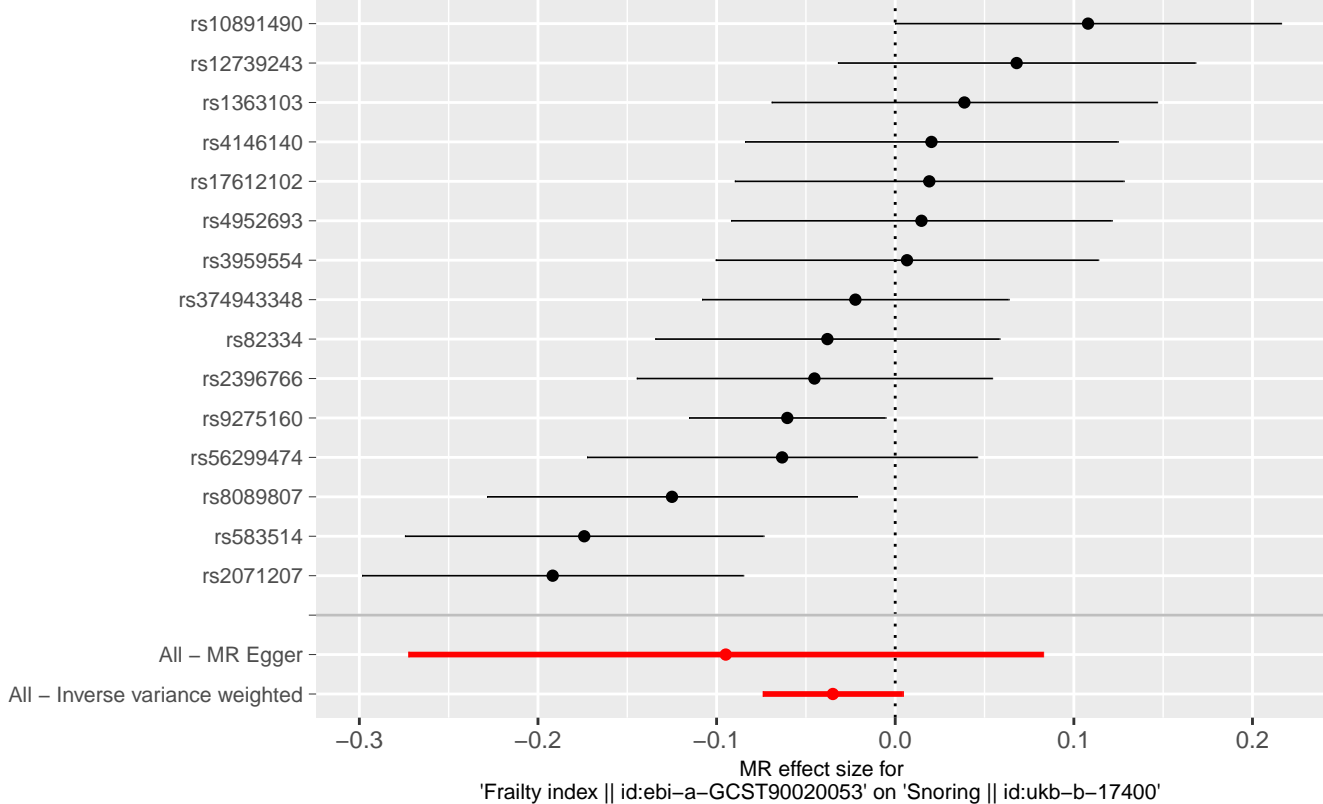

Supplement: Supplementary file 1 [file Data_Sheet_1.ZIP › Supplementary Materials/Snoring/reverse/Snoring reverse forest.pdf]

# MR Method

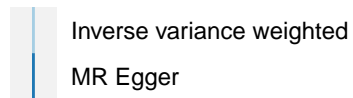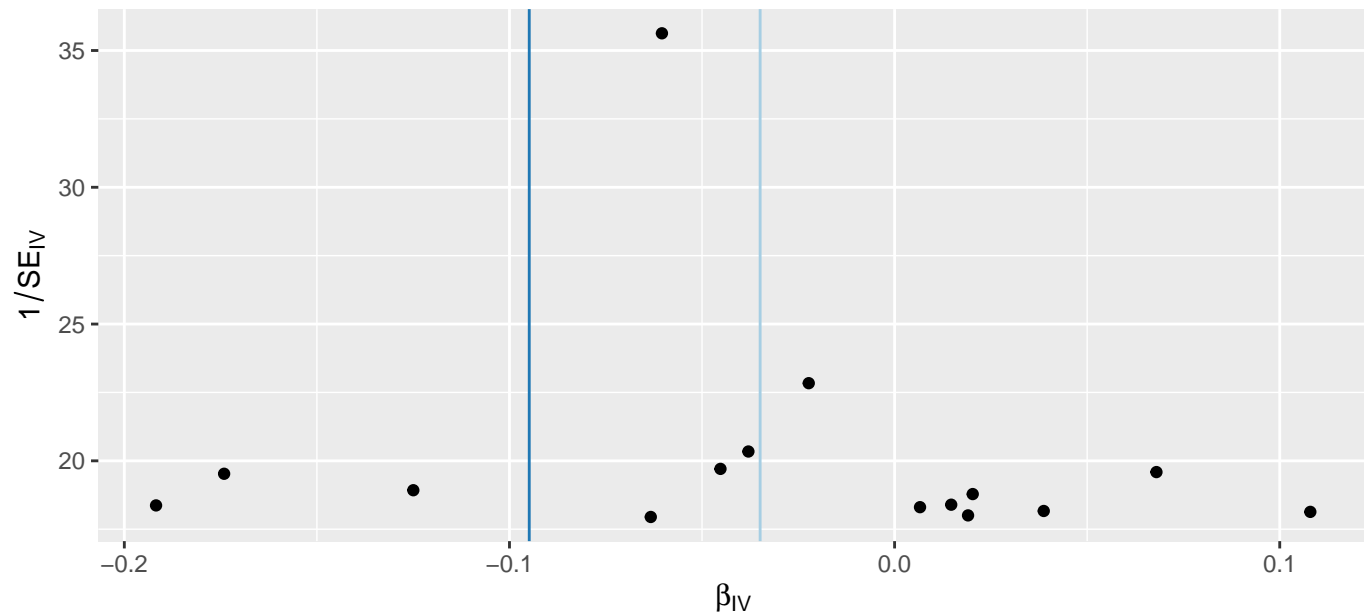

Supplement: Supplementary file 1 [file Data_Sheet_1.ZIP › Supplementary Materials/Snoring/reverse/Snoring reverse funnel.pdf]

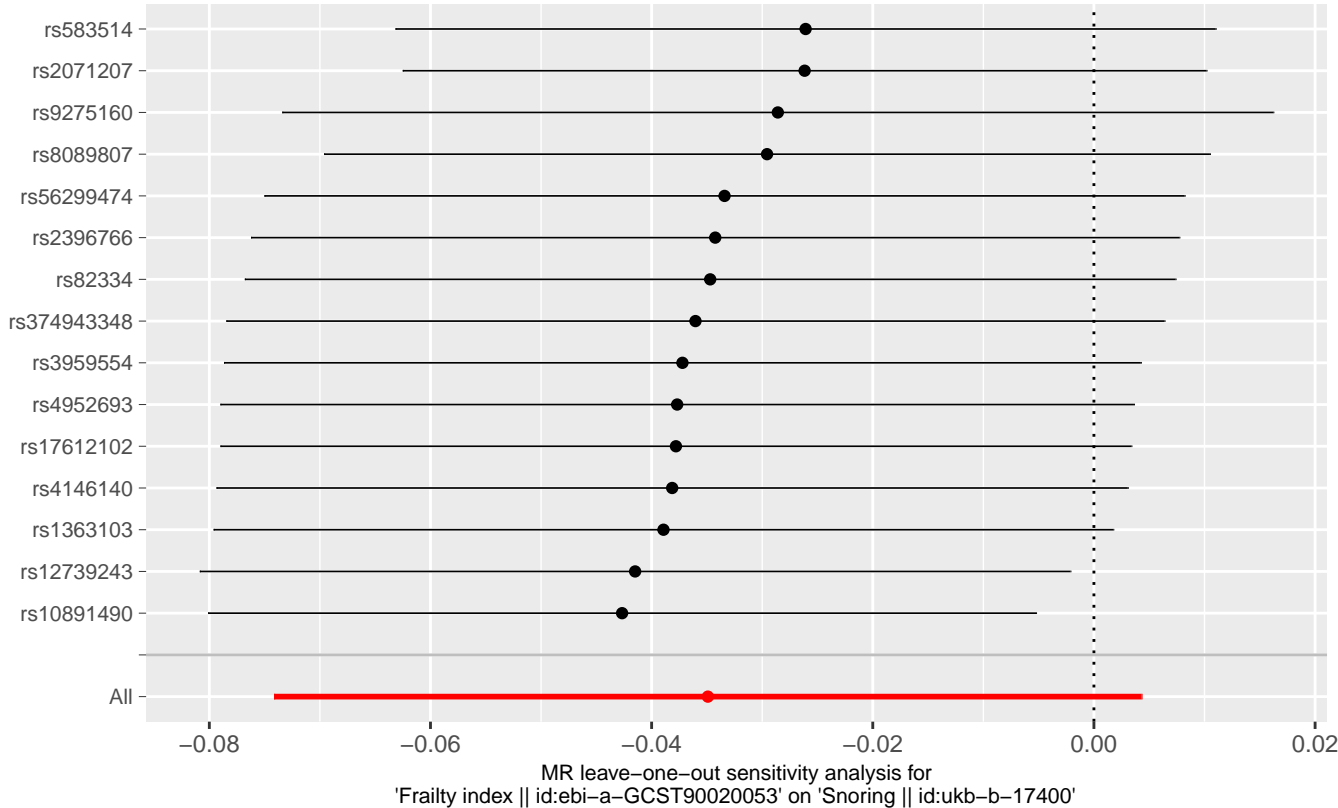

Supplement: Supplementary file 1 [file Data_Sheet_1.ZIP › Supplementary Materials/Snoring/reverse/Snoring reverse leave-one-out.pdf]

## MR Test

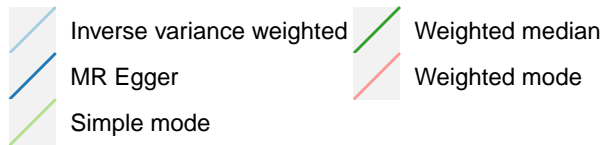

SNP effect on Snoring || id:ukb-b-17400

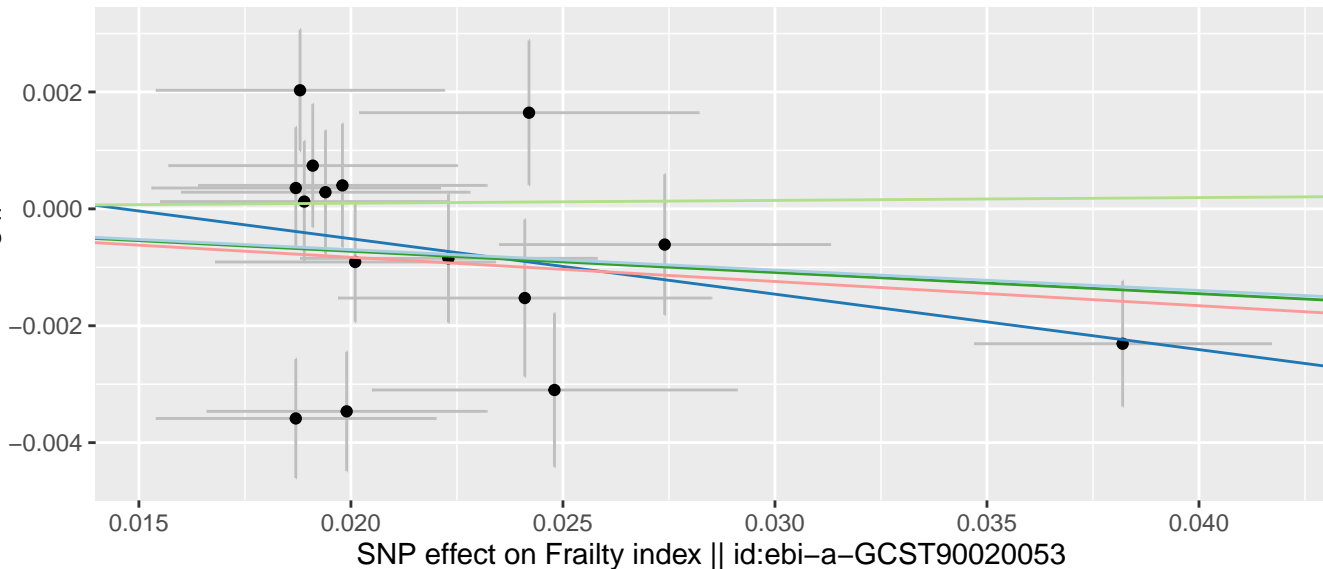

Supplement: Supplementary file 1 [file Data_Sheet_1.ZIP › Supplementary Materials/Snoring/reverse/Snoring reverse scatter.pdf]
